# KasQ an Epimerase Primes the Biosynthesis of Aminoglycoside Antibiotic Kasugamycin and KasF/H Acetyltransferases Inactivate Its Activity

**DOI:** 10.3390/biomedicines10020212

**Published:** 2022-01-19

**Authors:** Rajesh Rattinam, R. Sidick Basha, Yung-Lin Wang, Zhe-Chong Wang, Ning-Shian Hsu, Kuan-Hung Lin, Saeid Malek Zadeh, Kamal Adhikari, Jin-Ping Lin, Tsung-Lin Li

**Affiliations:** 1 Genomics Research Center, Academia Sinica, Taipei 115, Taiwan; rajeshbuibt@gmail.com (R.R.); sidickchem@gmail.com (R.S.B.); hcvns3@hotmail.com (Y.-L.W.); mournermind@gmail.com (Z.-C.W.); kevin320@gate.sinica.edu.tw (N.-S.H.); cardboy19821022@hotmail.com (K.-H.L.); saeid.malekzadeh@gmail.com (S.M.Z.); arsonkamal@yahoo.com (K.A.); jpl0815@gate.sinica.edu.tw (J.-P.L.); 2 Chemical Biology and Molecular Biophysics Program, Taiwan International Graduate Program, Academia Sinica, Taipei 115, Taiwan; 3 Institute of Bioinformatics and Structural Biology, National Tsing Hua University, Hsinchu 300, Taiwan; 4 Biotechnology Center, National Chung Hsing University, Taichung City 402, Taiwan

**Keywords:** kasugamycin, kasugamine, antibiotic biosynthesis, epimerase and acetyltransferase

## Abstract

Kasugamycin (KSM), an aminoglycoside antibiotic, is composed of three chemical moieties: D-*chiro*-inositol, kasugamine and glycine imine. Despite being discovered more than 50 years ago, the biosynthetic pathway of KSM remains an unresolved puzzle. Here we report a structural and functional analysis for an epimerase, KasQ, that primes KSM biosynthesis rather than the previously proposed KasF/H, which instead acts as an acetyltransferase, inactivating KSM. Our biochemical and biophysical analysis determined that KasQ converts UDP-GlcNAc to UDP-ManNAc as the initial step in the biosynthetic pathway. The isotope-feeding study further confirmed that ^13^C, ^15^N-glucosamine/UDP-GlcNH_2_ rather than glucose/UDP-Glc serves as the direct precursor for the formation of KSM. Both KasF and KasH were proposed, respectively, converting UDP-GlcNH_2_ and KSM to UDP-GlcNAc and 2-N’-acetyl KSM. Experimentally, KasF is unable to do so; both KasF and KasH are instead KSM-modifying enzymes, while the latter is more specific and reactive than the former in terms of the extent of resistance. The information gained here lays the foundation for mapping out the complete KSM biosynthetic pathway.

## 1. Introduction

**Figure 1:**
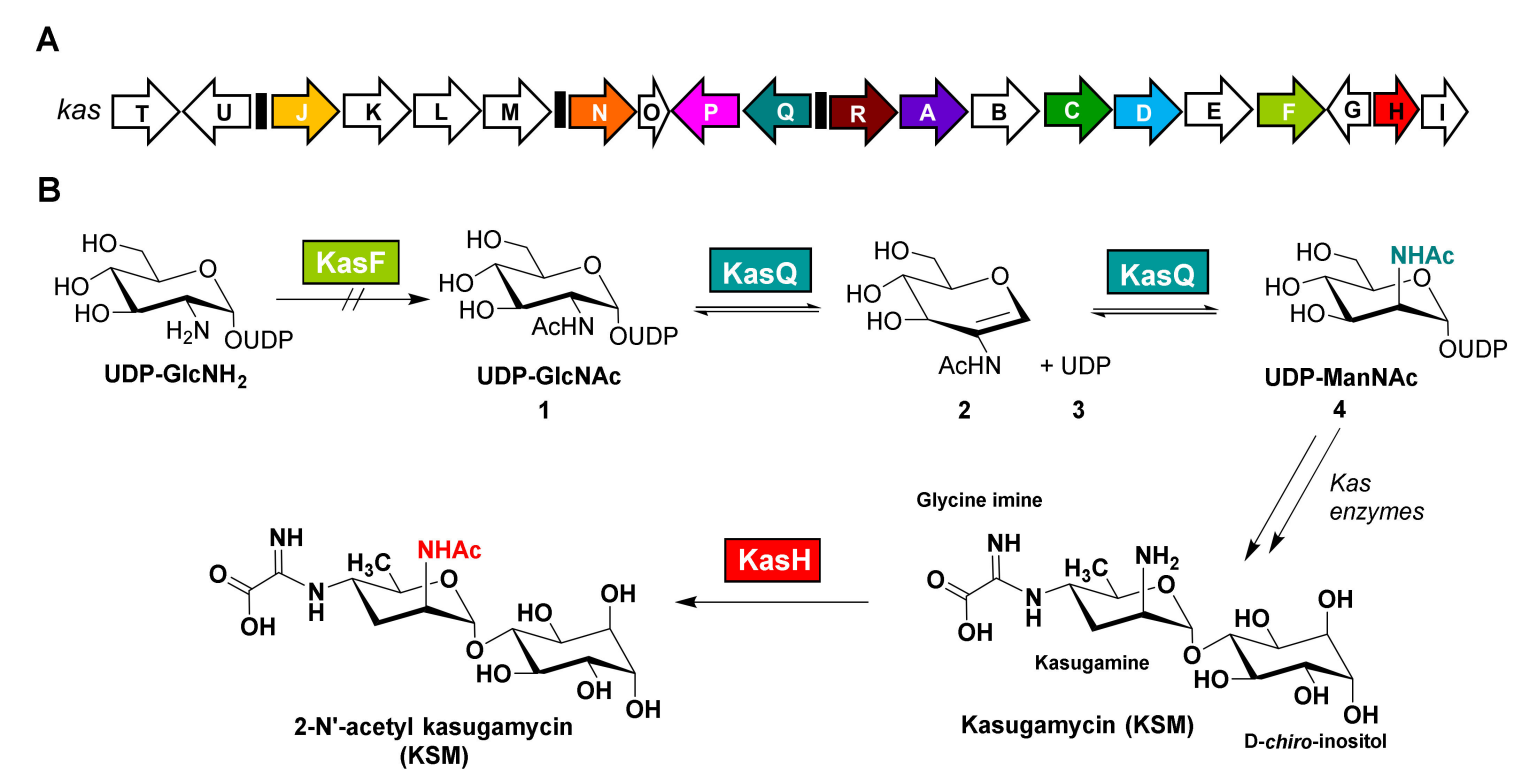
(**A**) The biosynthetic gene cluster (BGC) sequenced and identified from *Streptomyces kasugaensis*. (**B**) The proposed biosynthetic pathway of KSM based on the results revealed in this study. KasF is unable to acetylate UDP-GlcNH_2_ to UDP-GlcNAc. KasQ initiates the KSM biosynthesis by converting UDP-GlcNAc **1** to UDPManNAc **4** through AAG **2** and UDP **3** intermediates. KasH acts as a self-resistance enzyme to acetylate kasugamycin (KSM) to 2-N’-acetyl kasugamycin (Ac-KSM).

Kasugamycin (KSM), an aminoglycoside antibiotic produced by *Streptomyces kasugaensis*, was discovered in 1965. KSM is composed of three structurally distinct sub-components: a glycine imine, an unusual amino-sugar kasugamine and D-*chiro*-inositol (Figure 1) [1,2]. It has long been known that KSM binds specially to the interface between E- and P-site of the 30S ribosomal subunit to control fungal and bacterial protein translation [3]. KSM was used as an agricultural supplement to protect rice from fungus-derived diseases [4]. Beyond that, a number of new findings revealed that KSM possesses several unexpected biological activities effectively countering some recalcitrant human diseases: (i) an in vitro and in vivo study showed that the combination of KSM with rifamycin is in a position to control *Mycobacterium tuberculosis*, [5] (ii) KSM was recently shown to be capable of inhibiting herpes simplex virus-2 (HSV-2), [6] and (iii) KSM has also been shown to have a promising activity against COVID-19 by inhibiting 3a-channel [7] and chitinase 3-like-1 (CHI3L1) proteins [8]. We were attracted by these new findings and thus eager to investigate the biosynthetic pathway of KSM in the hope of generating new and useful KSM analogs in the future.

The KSM biosynthetic pathway was proposed on the basis of the gene sequence similarity of each gene encoded in its biosynthetic gene cluster (BGC) [9,10,11]. However, the gene products responsible for the formation of UDP-N-acetyl-glucosamine (UDP-GlcNAc) and *myo*-inositol, the possible starting units for the formation of the kaugamine moiety, were still unclear (Figure S1). Five enzymes (KasF codes for an acetyltransferase, KasD codes for a NAD-dependent dehydratase, KasR codes for a PLP-dependent dehydratase, KasP codes for an oxidoreductase, and KasC codes for an aminotransferase) were proposed to act on UDP-GlcNAc in sequence to form the UDP-kasugamine precursor. Two downstream gene products—KasN, a glycine oxidase, and KasQ, an epimerase—may drive reactions toward UDP-kasugamine and UDP-carboxyformimidoyl-kasugamine sequentially. KasA, a glycosyltransferase, could couple UDP-carboxyformimidoyl-kasugamine and *myo*-inositol to form a KSM disaccharide precursor, then KasJ, an epimerase, could complete the synthetic pathway by epimerizing the equatorial C1-OH of *myo*-inositol to D-*chiro*-inositol (with an axial C1-OH) to afford KSM (Figure S1) [9,10,11]. KasH was reported as a self-resistance enzyme inactivating KSM by N2′-acetylation [12]. F. Kudo et al. recently came up with a new biosynthetic pathway for KSM [13], whereas no intrinsic in vitro biochemical evidence was provided therein unequivocally delineating the actual roles of the enzymes designated in the biosynthesis of KSM. With the aim of determining the roles of these enzymes, our in silico analysis revealed that KasQ shares a high sequence similarity with many UDP-GlcNAc 2-epimerases, especially RffE (also referred to as WecB, 47% identity) and to SecA (44% identity). RffE converts UDP-GlcNAc to UDP-ManNAc in the early stage of enterobacterial common antigen (ECA) biosynthesis [14]. Likewise, SecA performs the same reaction in the first step of the capsular polysaccharide (CPS) biosynthesis [15]. L. Zhang et al. further examined the substrate specificity of SecA with a group of C2 N-acyl-modified UDP-GlcNAc and UDP-ManNAc derivatives, concluding that the C2 N-acyl group in the UDP-acylhexosamines is critical for molecular recognition [16]. Of these facts, we speculated that KasQ should stand in the early stage of the biosynthetic pathway (converting UDP-GlcNAc **1** to UDP-ManNAc **4**; Figure 1 and Figure S2) instead of the reported pathway where UDP-deoxy aminosugar is epimerized to UDP-kasugamine (Figure S1) [9,10,11]. This viewpoint, however, conflicted with the roles assigned for KasH and KasF; namely, they were proposed to prime and regulate the biosynthesis of KSM. We were thus prompted to resolve this long-standing issue by employing in vitro biochemical and biophysical analyses alongside in vivo isotope feeding assays, whereby we concluded that KasQ is the starter enzyme rather than KasF, and both KasF and KasH are self-resistant enzymes in the biosynthesis of KSM.

## 2. Materials and Methods

### 2.1. Cloning, Expression, and Purification

For the expression of KasQ, a gene was amplified from *Streptomyces kasugaensis* and cloned into expression vector pET28a+ to generate the protein with an N-terminal His_6_ tag. The recombinant plasmid was transformed into *Escherichia coli* BL21 competent cells for protein overexpression. The transformed BL21 cells were used to inoculate LB medium (1000 mL) containing 1 mM kanamycin and the culture was grown at 37 °C. After reaching the OD at A600 nm of 0.6–0.8, the culture was induced by the addition of 1 mM IPTG, followed by incubation at 16 °C overnight. Cells were harvested by centrifugation at 6000 rpm using a JLA8.1 rotor in a Beckman Coulter centrifuge for 25 min at 4 °C. The harvested (1000 mL) cells were resuspended in 20 mL of binding buffer (50 mM Tris pH 7.5, 500 mM NaCl, 10% glycerol, and 10 mM imidazole). Resuspended cells were lysed using a sonicator (time, 4 min, pulse, 3 s on and 2 s off). The resulting lysate was centrifuged at 18,000 rpm (JA20.50 rotor, Beckman Coulter, Brea, CA, USA) for 30 min at 4 °C. The supernatant was added to a Ni-agarose affinity column equilibrated with binding buffer. The unbound proteins were washed off using varied imidazole concentrations (10 mM, 25 mM, and 50 mM). The protein was then eluted with elution buffer (50 mM Tris pH 7.5, 500 mM NaCl, 10% glycerol, and 250 mM imidazole). The eluted protein samples were concentrated and injected into a Superdex S-200 column equilibrated with gel filtration buffer (50 mM HEPES pH 8.0, 100 mM NaCl). The peak fractions were collected and the purity of the KasQ protein was analyzed by 10% SDS-PAGE.

For the expression of KasH, a gene was amplified from *Streptomyces kasugaensis* and cloned into the expression vector pET28a+ to generate the protein with an N-terminal His_6_ tag. The recombinant plasmid was transformed into *E. coli* BL21 competent cells and the protein was overexpressed. The purification steps of the above procedure were followed with the different gel filtration buffer (50 mM MOPS pH 7.5, 100 mM NaCl). The purity of the KasH protein was analyzed by 10% SDS-PAGE and confirmed by mass spectrometry (peptide mass fingerprinting).

For the expression of KasF, the gene was amplified from *Streptomyces kasugaensis* and cloned into the expression vector pET28a+ to generate the protein with an N-terminal His_6_ tag. The recombinant plasmid was transformed into *E. coli* BL21 competent cells and the protein was overexpressed. The purification steps were followed by the above procedure with the different gel filtration buffer (50 mM HEPES pH 7.5, 200 mM NaCl). The purity of the KasF protein was analyzed by 10% SDS-PAGE and confirmed by mass spectrometry (peptide mass fingerprinting).

### 2.2. Crystallization and Data Collection

KasQ wild type (WT) and complex crystals were crystallized using the hanging drop vapor diffusion method at 20 °C. KasQ wild type (WT) protein was crystallized (7 mg mL^−1^) in a buffer (50 mM HEPES pH 8.0, 100 mM NaCl) using a reservoir solution (0.2 M potassium/sodium tartrate, 0.1 M Bis-Tris propane pH 7.5 and 20% PEG 3350). KasQ–WT crystals were formed after 7 days of incubation. The enzyme (10 mg mL^−1^) was co-crystallized with 2 mM UDP or 2 mM UDP-Glc in the presence of 2 mM MgCl_2_ with a reservoir solution containing 0.2 M lithium sulfate, 0.1 M Bis-Tris pH 5.5 and 25% PEG 3350 to obtain the KasQ–UDP or KasQ–UDP-Glc complex crystals. The complex crystals were formed after 15 days. Prior to the data collection, the crystals were transformed into the mother liquor containing 20% glycerol as a cryoprotectant and flash-frozen using liquid nitrogen. All the crystal data were collected in beamline 15A1 and 05A equipped with an ADSC Quantum-315 or MX300HE CCD detector (Rayonix, L.L.C., Evanston, IL, USA) at an operating temperature of 100K in National Synchrotron Radiation Research Center (NSRRC), Taiwan. The raw data were indexed and processed using the HKL2000 package (HKL Research, Inc., Charlottesville, VA, USA) [17]. The KasQ–WT crystal belongs to the C2 space group; the KasQ–UDP and KasQ–UDP-Glc complex crystals belong to the P1 space group. Detailed data collection statistics are shown in Table 1.

### 2.3. Structure Determination and Refinement

The KasQ–WT and its complex crystal structures were determined by molecular replacement (MR) with Phaser-MR from the CCP4 program suit [18]. The *E. coli* UDP-GlcNAc epimerase (PDB entry 1vgv) was used as a search model to solve the initial phase [19]. The protein structures were built and refined using REFMAC [20], COOT [21], and PHENIX [22]. PyMOL as used to construct the protein structures and electron density maps [23].

### 2.4. Site-Directed Mutagenesis

Site-directed mutagenesis was performed using QuikChange (Stratagene), following the manufacturer’s protocol. The sequences of primers used in this study to create KasQ mutants are shown in Table 2. The wild-type KasQ plasmid (BCRC12349) was used as the template for the single mutation. All mutations were confirmed by DNA sequencing. KasQ mutants were expressed and purified with the same protocol as used for the wild-type protein. 

### 2.5. Isothermal Titration Calorimetry (ITC)

ITC analyses were carried out at 25 °C using an ITC200 microcalorimeter (MicroCal Inc., Northampton, MA, USA). The buffer solution (50 mM HEPES pH 8.0, 100 mM NaCl) was used to prepare protein and substrates for ITC analysis. Each titration contained 0.1 mM KasQ in the sample cell (250 μL), and 1 mM substrates was loaded into the syringe (40 μL). The titration experiment contains 18 injections (2 μL for a duration of 4 s) and each injection was separated by 120 s. The distilled water was filled in the reference cell throughout all titrations. The cell stirring speed was 1000 rpm. The collected raw data were processed in Origin 7.0 (provided by MicroCal) by a non-linear least square method to a single-site binding model. KasH ITC analyses were followed using the above procedure with a buffer solution of 50 mM MOPS pH 7.5, 100 mM NaCl.

### 2.6. Synthesis of 2-Acetamidoglucal (AAG)

**Scheme 1:**
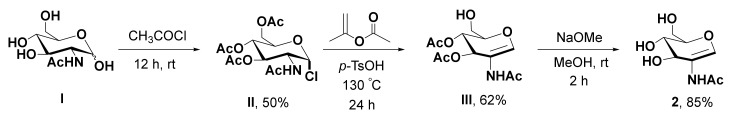
The synthetic steps for AAG **2**.

AAG **2** was synthesized as shown in Scheme 1. GlcNAc **I** reacts with acetyl chloride to yield glycosyl chloride **II**. Further reaction with isopropenyl acetate affords glucal **III**. Deacetylation of **III** using sodium methoxide provides **2**.

#### 2.6.1. General Information

^1^H and ^13^C NMR spectra were recorded on a Bruker 600 spectrometer with tetramethylsilane as the internal reference. ^31^P NMR was performed in Bruker Avance 500 AV NMR. Chemical shifts (δ scale) were reported in parts per million (ppm). ^1^H NMR spectra were reported in the order: multiplicity, number of protons, and coupling constant (J value) in hertz (Hz); signals were characterized as s (singlet), d (doublet), dd (doublet of doublet), and m (multiplet). Saturation transfer difference nuclear magnetic resonance (^1^H-STD NMR) was performed using a Bruker 600 MHz spectrometer, with KasQ and UDP-GlcNAc in a ratio of 1:100 recorded within 0–5 min. A saturation time of 2s was employed. UDP-GlcNAc-relative STD (saturation transfer difference) effects were determined by (*I*_0_ – *I*_sat_)/*I*_0_. (*I*_0_ – *I*_sat_) is the signal intensity in the STD NMR spectrum, whereas *I*_0_ is the signal intensity of the reference spectrum. Column chromatography was performed on silica gel (70–230 mesh). TLC analysis was carried out on Merck Silica gel 60 F254 plates using MOSTAIN/*p*-anisaldehyde sugar staining. Compound **2** was synthesized by following a slight modification of the procedure described in the literature [16].

#### 2.6.2. Procedure for the Synthesis of 2-Acetamidoglucal (AAG)

Acetyl chloride (6 mL), GluNAc **I** (2.00 g, 9.04 mmol) was added and stirred for up to 12 h. After that, CHCl_3_ (20 mL) was added and the mixture was poured into ice-cold water. The organic layer was washed with an aqueous sodium bicarbonate solution (20 mL) and concentrated under reduced pressure. The crude residue was purified by flash column chromatography to obtain the pure glycosyl chloride **II** (1.65 g, 50%). **^1^H NMR** (600 MHz, CDCl_3_) δ 6.18 (d, *J* = 3.7 Hz, 1H), 5.88 (d, *J* = 8.7 Hz, 1H), 5.31 (dd, *J* = 10.8, 9.4 Hz, 1H), 5.20 (dd, *J* = 11.0, 8.6 Hz, 1H), 4.53 (ddd, *J* = 10.7, 8.8, 3.7 Hz, 1H), 4.27 (m, 2H), 4.13 (m, 1H), 2.09 (s, 3H), 2.04 (s, 6H), 1.98 (s, 3H). **^13^C NMR** (150 MHz, CDCl_3_) δ 171.7, 170.8, 170.3, 169.3, 93.8, 71.1, 70.3, 67.1, 61.3, 53.7, 23.3, 20.9, 20.7. The resulting chloride **II** (1.40 g, 3.83 mmol) was treated with isopropenyl acetate (28 mL) and *p*-TsOH (45 mg) and was refluxed at 130 °C for 24 h. Then, the mixture was concentrated under reduced pressure and the crude residue was purified by flash column chromatography to attain the pure glucal product **III** (0.682 g, 62%). **^1^H NMR** (600 MHz, CDCl_3_) δ 6.60 (s, 1H), 5.56 (d, *J* = 4.8 Hz, 1H), 5.25 (m, 1H), 4.51 (m, 1H), 4.47 (m, 1H), 4.19 (dd, *J* = 12.0, 3.0 Hz, 1H), 2.09 (s, 6H), 2.01 (s, 3H). **^13^C NMR** (150 MHz, CDCl_3_) δ 170.7, 170.0, 169.7, 148.0, 113.5, 74.9, 67.4, 67.1, 61.1, 20.9, 20.8. To a glucal **III** (0.600 g, 2.09 mmol), dry methanol (14 mL) was added and dissolved, then sodium methoxide was added (pH ~8.5) and the mixture was stirred for up to 2 h. The mixture was neutralized with Amberlite IR-120 (H+), filtered, and concentrated. The crude residue was purified by flash column chromatography and the pure solid product **2** (0.360 g, 85%) was obtained. **^1^H NMR** (600 MHz, D_2_O) δ 6.61 (s, 1H), 4.18 (d, *J* = 6.6 Hz, 1H), 3.91 (m, 1H), 3.79 (d, *J* = 4.0 Hz, 2H), 3.69 (dd, *J* = 9.2, 6.8 Hz, 1H), 1.97 (s, 3H). **^13^C NMR** (150 MHz, D_2_O) δ 174.5, 141.7, 113.2, 78.5, 68.8, 68.4, 59.9, 21.9.

### 2.7. Construction of pMKBAC08-KAS and pWZC07-PrpsJ-kasT

The *E. coli-Streptomyces* spp. shuttle BAC plasmid, pMKBAC08, was designed by amplifying the DNA segments containing the origin of transfer region (*oriT*) and the integrase and apramycin resistance gene, *aacIII(IV)*, within the primer set P325 and P326 from plasmid pGUSRolRPA3 [24], and inserted into the BAC plasmid pBeloBAC11 (New England Biolabs, NEB).

pMKBAC08-KAS-Int, which carries an intact kasugamycin BGC, was constructed via sequentially cloning partial segments from the kasugamycin BGC into the pMKBAC08 plasmid as reported in previous studies [11]. Briefly, the DNA fragments containing the *kasT* gene, *kasU-kasM*, *kasN-kasQ*, *kasR-kasB*, and *kasC-kasI* were amplified with the primer sets P115/P116, P117/P118, P119/120, P121/P122m and P123/P124 (Table 3). After digesting the amplicons by different restriction enzymes and individually introducing them into the pMKBAC08 vector using T4 DNA ligase, the BAC-containing kasugamycin BGC, pMKBAC08-KAS, was obtained.

The *E. coli-Streptomyces* spp. shuttle plasmid, pWZC07, was constructed by replacing the apramycin resistant gene, *aacIII(IV)*, in the plasmid pGUSRolRPA3 [24] with the neomycin resistant gene, *neo (Tn5)*. To activate the expression of kasugamycin, the pWZC07-P*rpsJ-kasT* plasmid was constructed by cloning the *rpsJ* promoter fused with the *kasT* activator gene into the pWZC07 vector as reported previously [11].

### 2.8. In Vivo Isotope Incorporation Assay

For investigating the incorporation of the isotope-labeled compounds, *Streptomyces lividans* TK64 was introduced with pMKBAC08-KAS and pWZC07-P*rpsJ-kasT*. After being grown on an MS agar plate at 28 °C for 7 days [25], the grown spores were inoculated into flasks (50 mL volume) containing 10 mL of KPMb medium (maltose monohydrate, 15 g/L; bacto-yeast extract, 4.0 g/L; phytone-peptone, 15 g/L; MgSO_4_∙7H_2_O, 0.5 g/L; KH_2_PO_4_, 1.0 g/L; and NaCl, 3.0 g/L.) and incubated at 28 °C for 5 days [11]. After 24 h of incubation, isotope-labeled precursors (^13^C1-glucose; ^13^C1-glucosamine; ^13^C1-^15^N2-glucosamine; ^15^N-glycine; ^15^N-aspartic acid; and ^15^N-glutamic acid) were prepared and added into the culture individually every 24 h until 72 h (17 mg/mL). After shaking for 96 h, the mycelium was removed from the culture broth and the filtrate was adjusted to pH 4.0 with oxalic acid. The treated samples were incubated at 70 °C for 20 min and then the supernatant was collected and analyzed after removing the debris by centrifuge. Supernatants were injected into the HPLC-TQ-MS and separated by the method described below.

HPLC method A:

The supernatant was subjected to HPLC-TQ-MS with a Bridge BEH amide column (250 mm × 4.6 mm, 5 µm). The mobile phase was set up with a linear gradient, 30% H_2_O + 0.1 formic acid (FA) and 70% acetonitrile, at a flow rate of 1.0 mL min^−1^ over 30 min.

### 2.9. Disc Diffusion Assay

KSM susceptibility and production were confirmed by disc diffusion assay. An *E. coli* culture was grown at 37 °C overnight and spread on an LB agar plate (pH 7.0). About 5 µL of extracted samples from *Streptomyces lividans* TK64 (from days 2–5) were loaded onto the paper disc. The disc was placed onto the surface of an LB agar plate and incubated at 37 °C overnight to visualize the zone of inhibition (ZOI).

For the KSM resistant assay, BL21 cells with/without the *kasH* or *kasF* gene were individually expressed and seeded on LB agar plates (pH 7.0). About 5 µL of different aminoglycoside antibiotics (amikacin, gentamicin, sisomicin, streptothricin-F, or tobramycin) were loaded onto each paper disc and placed on the surface of the LB plates. The plates were incubated 37 °C overnight to visualize the appearance of the zone of inhibition (ZOI). Note: this is a standard protocol for evaluating the effectiveness of common antibiotics, including aminoglycosides; there is no apparent impact concerning the structural integrity or biological deterioration of the selected aminoglycosides at pH 7.0 during the examining course.

### 2.10. HPLC Activity Assay

The epimerization reaction was carried out in a 100 µL reaction mixture containing 0.1 mg freshly purified KasQ, 1 mM UDP-GlcNAc, and 1 mM MgCl_2_ in 25 mM Tris buffer, pH 8.0. The reactions were incubated at 37 °C for 12 h and quenched by adding equal volumes of chloroform. The precipitated protein was removed from the reaction mixture by centrifugation at 15,000 rpm for 20 min. For the screening of different NDP-sugar substrates, the enzymatic reactions were carried out in a 100 µL reaction mixture containing 0.1 mg KasQ, 1 mM NDP-Sugars (UDP-GalNAc, UDP-Glc, TDP-Glc, GDP-Glc, and UDP-Gal) in the presence of 1 mM MgCl_2_ in 25 mM Tris buffer, pH 8.0. The reactions were incubated at 37 °C for 12 h and quenched by adding equal volumes of chloroform. The precipitated protein was removed from the reaction mixture by centrifugation at 15,000 rpm for 20 min. Supernatants were injected into the HPLC-LTQ-MS and separated by the method described below.

HPLC method B:

The supernatant was subjected into HPLC-LTQ-MS with a Hypercarb Porous Graphitic Carbon Column (100 mm × 4.6 mm, 5 µm, Thermo Fisher Scientific, USA). The solvents used in the mobile phases were: solvent A: 50 mM ammonium formate; solvent B: 100% acetonitrile. A gradient started at 0–5 min (solvent A: 95%; solvent B: 5%) and progressed to 18 min (solvent A: 60%; solvent B: 40%) and 24–26 min (solvent A: 2%; solvent B: 98%) and ended at 28–32 min (solvent A: 95%; solvent B: 5%) at a flow rate of 1.0 mL min^−1^.

The acetylation reactions were carried out in a 100 µL reaction mixture containing 0.1 mg KasH or KasF, 1 mM KSM or UDP-GlcNH_2_, and 0.5 mM AcCoA in 25 mM Tris buffer, pH 8.0. The reactions were incubated 12 h at 37 °C and quenched by adding equal volumes of chloroform. Supernatants were injected into the HPLC-LTQ-MS and separated by the method described below.

HPLC method C:

The supernatant was subjected into HPLC-LTQ-MS with a Phenomenex Prodigy C-18 column (250 mm × 4.6 mm, 5 µm). The solvents used in the mobile phases were: solvent A: H_2_O + 0.1% trifluoroacetic acid (TFA); solvent B: acetonitrile (ACN) + 0.1% TFA. A gradient started at 0–5 min (solvent A: 98%; solvent B: 2%) and progressed to 18 min (solvent A: 60%; solvent B: 40%) and 24–32 min (solvent A: 2%; solvent B: 98%) and ended at 35–41 min (solvent A: 98%; solvent B: 2%) at a flow rate of 1.0 mL min^−1^.

### 2.11. Thin Layer Chromatography (TLC) Analysis

About 1–2 µL of AAG **2** and reaction mixture of KasQ with UDP-GlcNAc and MgCl_2_ were spotted onto a TLC plate (Merck Silica gel 60 F254 plates). For aminoglucal detection, TLC was soaked with *p*-anisaldehyde stain solution and, upon gentle heating on a hot plate, the red color spot was visualized.

### 2.12. HPLC Kinetic Analysis

Kinetic analysis was performed in a 100 µL reaction mixture containing 0.1 mg freshly purified KasQ, six different concentrations of UDP-GlcNAc (0.25, 0. 50, 0.75, 1.00, 1.25, and 1.50 mM) and 1 mM MgCl_2_ in 25 mM Tris buffer, pH 8.0. The reaction mixture was mixed by vortex and incubated at 37 °C. Reactions were stopped at six different time points (10, 30, 60, 120, 180, and 240 min) by adding equal volumes of chloroform. Precipitation was removed by centrifugation and analyzed by HPLC-LTQ-MS using HPLC method B as described above. Substrate (UDP-GlcNAc **1**) peaks were eluted at 10.69 min and products were (UDP-ManNAc **4**) eluted at 9.90 min, whereas the intermediates UDP **3** and AAG **2** were eluted at 9.51 min and 8.99 min, respectively. The Michaelis–Menten curve of the KasQ activity was derived from the integrated peak area of each reaction.

### 2.13. NMR Activity Assay

Enzymatic reactions were performed in 25 mM Tris pH 8.0 with 1 mg freshly purified KasQ, 15 mM UDP-GlcNAc, and 5 mM MgCl_2_ in a total reaction volume of 600 µL, incubated at 37 °C for 4 h. After incubation, the reaction was quenched by adding an equal volume of chloroform, and the supernatant was collected by centrifugation. The supernatant was filtered through a 3 kDa cut-off filter membrane (Millipore) and freeze-dried using a lyophilizer. The lyophilized product was dissolved in D_2_O (500 µL) and analyzed by NMR. For standards, a pure UDP-GlcNAc **1**, AAG **2**, and UDP **3** were dissolved in D_2_O (500 µL) and analyzed by NMR.

Similarly, time course NMR samples were prepared. The enzymatic reactions were stopped at six different time points (10 min, 30 min, 60 min, 120 min, 180 min, and 12 h) by adding an equal volume of chloroform, and supernatants were collected by centrifugation. The supernatants were filtered through a 3 kDa cut-off filter membrane (Millipore) and freeze-dried using a lyophilizer. The lyophilized product was dissolved in D_2_O (500 µL) and analyzed by ^1^H and ^31^P NMR spectroscopy.

### 2.14. Deuterium Incorporation Assay

Each enzymatic reaction was performed in a 100 µL reaction mixture containing 1 mM UDP-GlcNAc and 1 mM MgCl_2_, incubated with or without KasQ in D_2_O. Deuterium incorporation was stopped at nine different time points (0 min, 2 min, 5 min, 10 min, 15 min, 20 min, 25 min, 30 min, and 12 h) by adding equal volumes of chloroform. Supernatants were injected into the HPLC-LTQ-MS and separated by the method described below.

HPLC method D:

The supernatant was subjected to HPLC-LTQ-MS with a Phenomenex Prodigy C-18 column (250 mm × 4.6 mm, 5 µm). The solvents used in the mobile phase were: solvent A: 50 mM ammonium formate; solvent B: 100% acetonitrile. A gradient started at 0–6 min (solvent A: 92%; solvent B: 8%) and progressed to 7–10 min (solvent A: 70–50%; solvent B: 30–50%) and 11–21 min (solvent A: 98%; solvent B: 2%) at a flow rate 0.3 mL min^−1^.

### 2.15. Computational Analysis

For sequence alignment, KasQ homologue protein sequences were retrieved from the National Center for Biotechnology Information (NCBI). Multiple sequence alignment was performed with Clustal Omega and the *aln* format file was uploaded into EsPript to construct the final image (Figure S21) [26]. KasQ homolog selected active sites were uploaded to the Weblogo server to build the KasQ active site sequence logo [27].

## 3. Results and Discussion

### 3.1. Biochemical Investigation of an Epimerase

**Figure 2:**
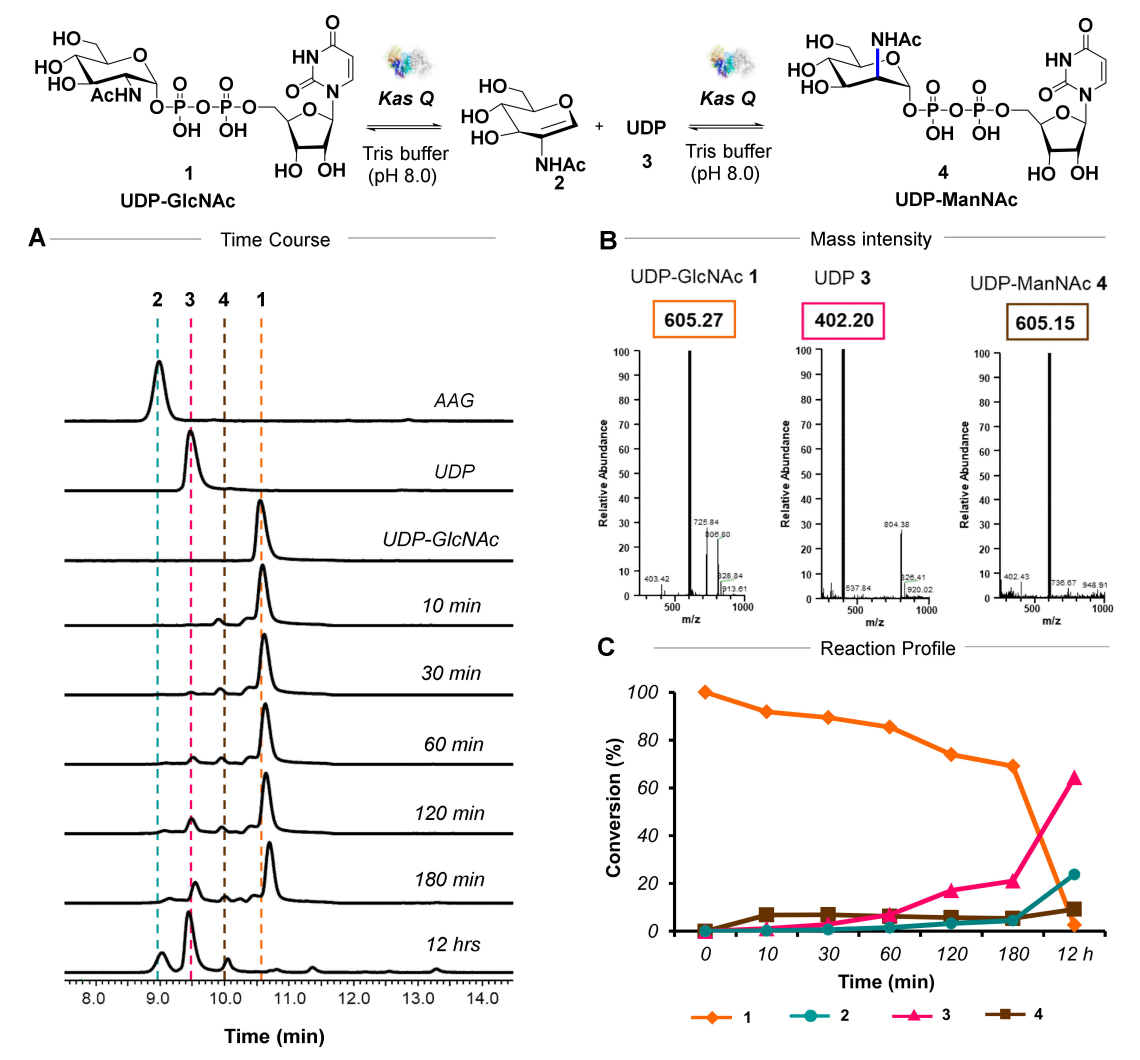
Biochemical investigation of KasQ by HPLC-LTQ-MS. (**A**) Time course assay for the KasQ-mediated reaction in the presence of UDP-GlcNAc. The assay scheme is shown at the top: **1**. UDP-GlcNAc, **2**. AAG, **3**. UDP, and **4**. UDP-ManNAc. (**B**) The *m*/*z* of UDP-GlcNAc, UDP-ManNAc, and UDP for the reaction carried out by KasQ in the presence of UDP-GlcNAc and MgCl_2_ for 180 min. (**C**) The conversion rate of UDP-GlcNAc (orange) to UDP-ManNAc (brown), UDP (pink), and AAG (cyan) over 12 h time course.

In an attempt to validate the biochemical function of KasQ, N-terminally His-tagged KasQ was purified, which exists in solution as dimeric proteins (Figure S3). At first, potential substrates/intermediates UDP-GlcNAc **1**, 2-acetamidoglucal **2** (AAG), and UDP **3** were analyzed by HPLC-MS. Next, enzymatic reactions of KasQ incubated with UDP-GlcNAc and MgCl_2_ were examined at various time points (Figure 2A,B and Figure S4). At 10 min, the expected product, UDP-ManNAc **4** (*m*/*z* 605.15 [M-H]), appeared at the retention time of 9.90 min. On an elevated timeline, AAG **2** and UDP **3** (*m*/*z* 402.20 [M-H]) emerged, respectively, at 8.99 and 9.51 min, and then increased over time (Figure S5). The generation of UDP-ManNAc **4** stagnated after 10 min and slightly increased at 12 h. The optimum temperature for KasQ epimerization activity was 37–42 °C. (Figure S6). Next, the efficiency of the epimerase reaction was investigated. UDP-ManNAc formation was 6.8% at 10 min and marginally increased to 9.2% at 12 h. On the other hand, at 10 min AAG and UDP were formed at 0.2% and 1.1%, respectively. At 12 h, the conversion of AAG was 23.8% and that of UDP was 64.4%. UDP-ManNAc concentration was 2.6-fold lower than that of AAG and seven-fold lower than that of UDP, which indirectly suggested that the intermediates formed are thermodynamically stable, consistent with previous studies (Figure 2C and Figure S7) [14].

**Figure 3:**
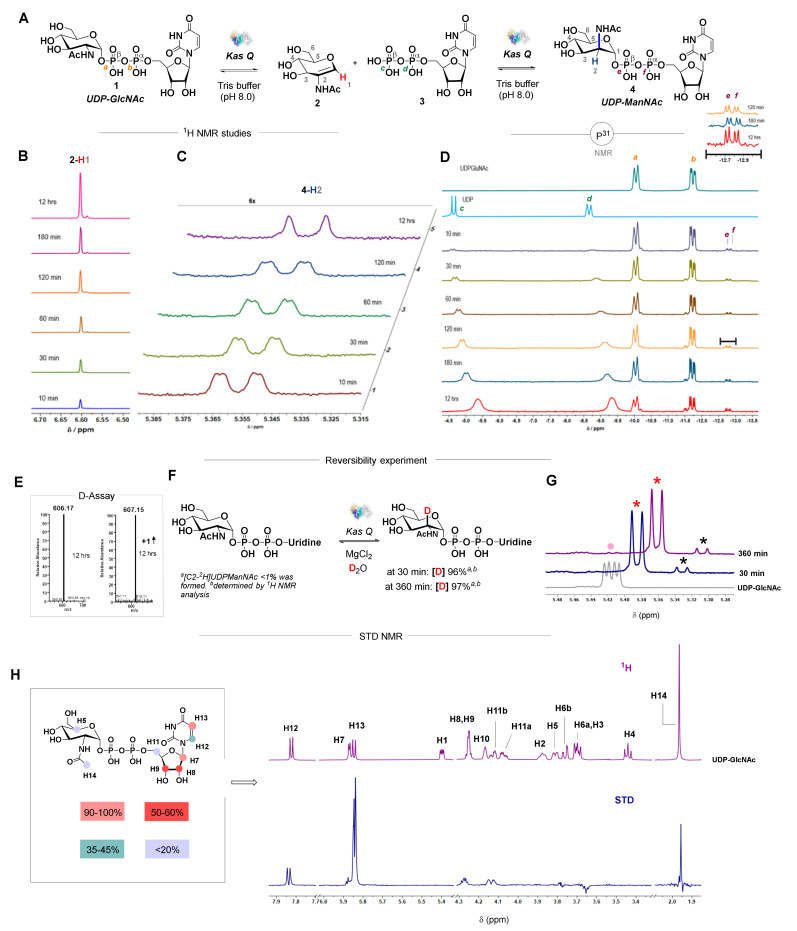
Biochemical investigation of KasQ by NMR and mass spectrometry. (**A**) Scheme of the KasQ-mediated epimerization reaction. Proton and phosphorous labels are shown in structural formulas. (**B**) The ^1^H NMR stack plot of AAG evolution. (**C**) Time course ^1^H NMR spectra during UDP-ManNAc formation. (**D**) The ^31^P NMR stack plot of varied time intervals. (**E**) The deuterium incorporation assay with or without KasQ was incubated with UDP-GlcNAc and MgCl_2_ in D_2_O. (**F**,**G**) Reversibility experiment. The pink circle denotes anomeric protons of UDP-GlcNAc; red and black asterisks are the C2 deuterated UDP-GlcNAc and UDP-ManNAc anomeric protons. (**H**) ^1^H-STD NMR analysis of UDP-GlcNAc in the presence of KasQ and the relative STD effects are shown in appropriate colors. The highest intensity of the STD signal signifies 100%, according to which the other signals were respectively calculated.

The KasQ epimerase reaction was parallelly probed by NMR spectrometry. The reaction of UDP-GlcNAc in the presence of KasQ and MgCl_2_ emanated the peak of a singlet at 6.60 ppm corresponding to an alkenyl proton of AAG and an appreciable amount of UDP-ManNAc was determined by its anomeric proton at 5.35 ppm. In contrast, a lesser number of UDP-GlcNAc anomeric protons at 5.41 ppm was observed (Figure S8). Extensive studies at various time intervals of the enzymatic reaction were executed (Figure 3). AAG formed at 10 min and increased consistently with respect to time (Figure 3B), whereas UDP-ManNAc formation from 10–120 min remained almost the same and slightly increased at 12 h (Figure 3C). The equilibrated existence of phosphorus in the epimerase reaction was tracked at various time periods by P^31^ NMR spectrometry. At 10 min, UDP extrusion was observed with P_α_ at −8.7 ppm and P_β_ at −4.5 ppm, along with UDP-GlcNAc P_α_ at −11.7 ppm and P_β_ at −10.0 ppm. At a wider timespan, the UDP-GlcNAc peak was reduced and the UDP peak was intensified, whereas UDP-ManNAc P_α_ and P_β_ at −12.8 and −12.7 ppm moderately increased with respect to time (Figure 3D). Enzymatic isotope labeling studies of UDP-GlcNAc were scantly documented [14,28]. To gain further insight into the KasQ epimerization reaction, a reversibility experiment was performed. UDP-GlcNAc with or without KasQ in the presence of MgCl_2_ was incubated in D_2_O at different time intervals and the reaction mixtures were subjected to HPLC-MS analysis (Figure S9). No deuterium scrambling was observed when UDP-GlcNAc was incubated in D_2_O without KasQ up to 12 h (*m*/*z* 605.98 [M-H]) (Figure 3E and Figure S9A). Moreover, in the presence of KasQ with UDP-GlcNAc and MgCl_2_ in D_2_O, deuterium was labeled from 2 min to 12 h (*m*/*z* 606.96 [M-H]) (Figure 3E and Figure S9B). To validate the position of the deuterium incorporation, NMR studies were performed (Figure 3F,G). The C2 position of the UDP-GlcNAc was deuterated (97%) and appeared as a doublet at 5.36 ppm with minor traces of C2-deuterated UDP-ManNAc (<1%). Heavily incorporated deuterium at the C2 position of the UDP-GlcNAc clearly suggests that the abstraction of protons at the C2 position is highly reversible with the surrounding active-site residues of KasQ. It also postulates that first step of the reaction mechanism is highly reversible. A control experiment was performed to recognize the ability of thermodynamically stable AAG with UDP to generate UDP-ManNAc or UDP-GlcNAc. KasQ incubated with AAG and UDP in the presence of MgCl_2_ was monitored by ^1^H NMR (Figure S12). The absence of anomeric proton signals of UDP-ManNAc or UDP-GlcNAc concluded that the enzymatic reaction was ultimately futile. A similar finding was also noticed with SacA [16]. An ITC experiment for AAG with KasQ showed unfavored binding in the medium (Table 4). This implies that, thermodynamically, there is no binding affinity with KasQ for AAG. Enzyme binding interactions of UDP-GlcNAc were determined by saturation transfer difference (STD) NMR (Figure 3H). The proton binding epitope of UDP-GlcNAc with KasQ suggested that the ribose framework accumulated a major zone of interactions, whereas the GlcNAc and uridine moieties occupied a minor zone with KasQ. Both uridine H13 (the alkenyl group) and ribose H7 bind closely with KasQ, acquiring major STD effects. Moderate binding was attained on H8 and H9 of the ribose ring. GlcNAc H5, H14, and ribose H11 formed weak binding interactions with KasQ. Based on these binding studies of STD NMR and ITC analyses, we propose that the KasQ epimerase may proceed through a kinetically favorable or an enzyme-bound reaction mechanism. The proposed pathway for this epimerization reaction is from UDP-GlcNAc which undergoes an *anti*-elimination to generate AAG and UDP. Then, it undergoes subsequent *syn*-addition to afford an epimer UDP-ManNAc **4**. Overall, our biochemical investigations indicate that KasQ converts UDP-GlcNAc **1** to UDP-ManNAc **4** in a shorter reaction time, whereas in a prolonged reaction it releases a higher amount of AAG **2** and UDP **3**.

### 3.2. Biochemical Characterization of Acetyltransferases

**Figure 4:**
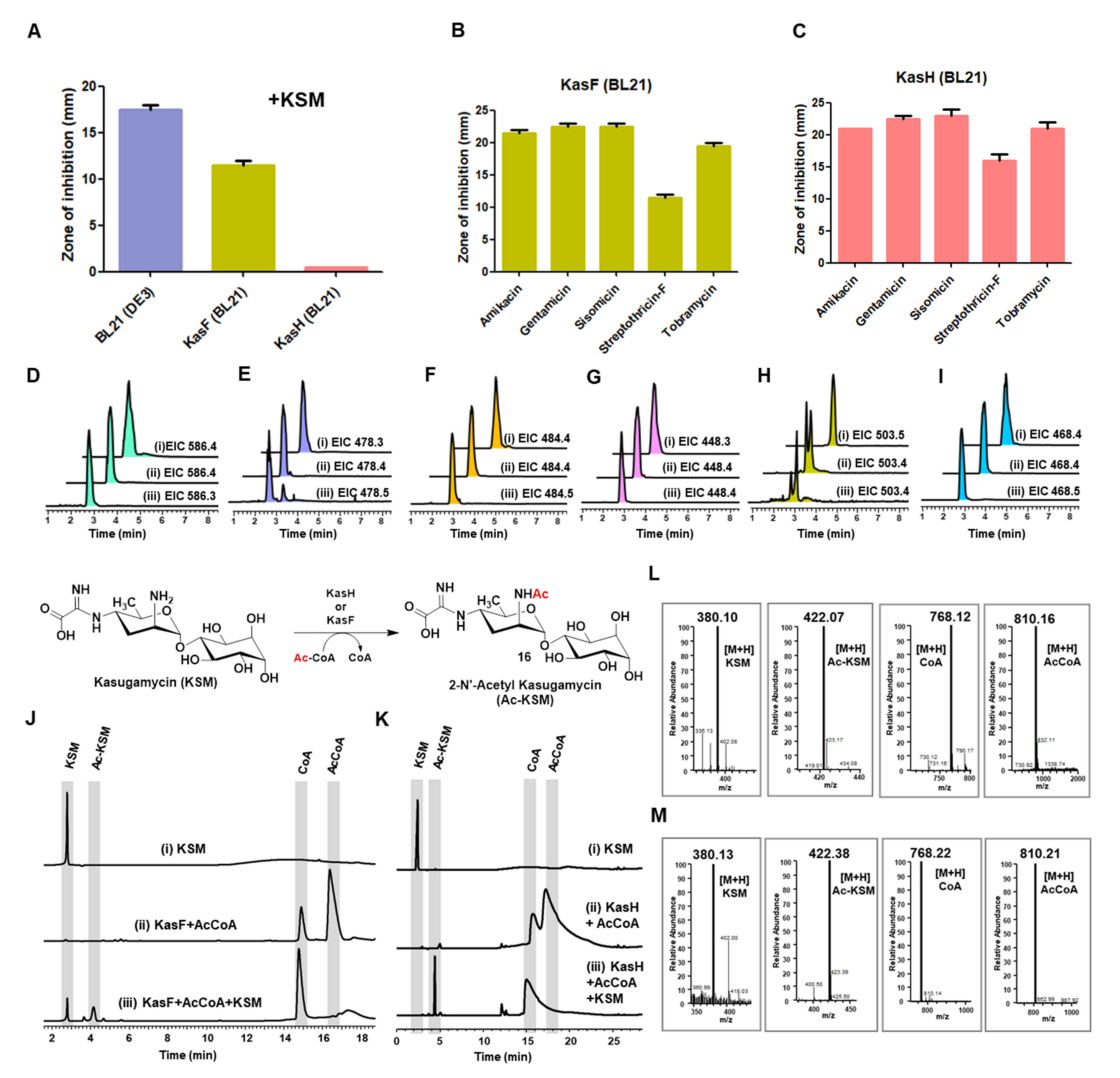
Biochemical characterization of acetyltransferases KasF and KasH. (**A**) Disc diffusion assay with or without KSM. The *E. coli* BL21 (DE3) strain was used as a control. (**B**,**C**) The in vivo resistance activity of KasF and KasH was assayed against amikacin, gentamicin, sisomicin, streptothricin-F, and tobramycin. (**D**–**I**) The acetylation reactions of KasF and KasH were examined in the presence of AcCoA and different aminoglycoside antibiotics, (**D**) amikacin, (**E**) gentamicin, (**F**) kanamycin B, (**G**) sisomicin, (**H**) streptothricin-F, and (**I**) tobramycin. The EICs of (i) the antibiotic alone, (ii) KasF with the selected antibiotic and AcCoA, (iii) KasH with the selected antibiotic and AcCoA are displayed. (**J**,**K**) The acetylation reactions of KasF and KasH were examined in the presence of KSM and AcCoA, indicating that both KasF and KasH are specific to KSM. (**L**,**M**) MS spectra for the substrates and products (in *m*/*z*) of the KasF- and KasH-mediated reactions.

The primary sequence analysis revealed that both KasF and KasH belong to enzymes of the acetyltransferase superfamily, while both share very low sequence identity (<10%) (Figure S13). Purified KasF forms monomers but KasH forms dimers in solution (Figure S3). The in vivo acetylation (resistance) activity of KasF and KasH versus KSM was evaluated with a disc diffusion assay (Figure S14). Individually, we introduced the pET28a+ plasmid carrying the *kas*F or *kas*H gene into the BL21 (DE3) *E. coli* cells. The *E. coli* cells (carrying the empty pET28a+ plasmid without the given acetyltransferase gene) were used as a control. We observed a clear zone of inhibition (ZOI) in the *E. coli* cells (not carrying the acetyltransferase gene) representing the cells’ inability to grow in the presence of KSM (the disc contained ~5 μL of 7.6 mg/mL of KSM). The growth of *E. coli* that carried the *kas*H gene was not affected in the presence KSM, whereas moderate growth of *E. coli* was observed in the cells carrying the *kas*F gene (Figure 4A and Figure S14). This phenomenon indicated that KasH serves as a resistance gene specific to KSM. Whether KasF is a resistance gene against other aminoglycoside antibiotics was further examined (Figure 4B,C and Figure S15). The activities of these two acetyltransferases against a number of commonly used aminoglycoside antibiotics (amikacin, gentamicin, kanamycin B, sisomicin, streptothricin-F, and tobramycin) were examined. The appearance of ZOI in all conditions strongly suggested that neither KasH nor KasF are active towards these aminoglycoside antibiotics (Figure 4B,C and Figure S15). Based on this in vivo assay we concluded that KasH serves as a self-resistance gene, while KasF partially acts as a self-resistance gene to KSM. To support this finding, an in vitro assay was performed. KasF or KasH were incubated with 1 mM KSM and 0.5 mM AcCoA at 37 °C for 12 h. KasH completely acetylated KSM (*m*/*z* 380.16 [M+H]) to produce 2′N-acetyl-kasugamycin (Ac-KSM; *m*/*z* 422.38 [M+H]). Consistent with the in vivo analysis, KasF partially acetylated KSM (*m*/*z* 380.10 [M+H]) to form Ac-KSM (*m*/*z* 422.07 [M+H]) (Figure 4J–M). As a result, KasH is the main self-resistance enzyme in the biosynthesis of KSM, but *KasF* may play a supportive role in the overall resistance system. Additionally, both KasF and KasH that are unable to acetylate the generally used aminoglycoside antibiotics (Figure 4D–I) suggested that the two enzymes are a pair of resistance enzymes specific to KSM, the latter stronger than the former in terms of antimicrobial effectiveness. The specificity of KasF and KasH is due likely to their regioselectivity on KSM because it is an atypical aminoglycoside antibiotic, thereby incapable of acetylating other aminoglycosides. Isothermal titration calorimetry (ITC) was used to quantify the binding affinity of KasH versus CoA (*K*_d_ = 96.1 µM), AcCoA (*K*_d_ = 160.7 µM), and KSM (*K*_d_ = 56.1 µM). Among these binding affinity parameters, KasH and KSM concentrations are 2.9- and 1.7-fold higher than AcCoA and CoA, respectively (Figure S17 and Table S1). Although KasF is capable of partially acetylating KSM, it may not be a direct substrate. In the proposed biosynthetic pathway, KasF catalyzes the first step of reactions by converting UDP-GlcNH_2_ to UDP-GlcNAc. To check this proposition, KasF was incubated with 1 mM UDP-GlcNH_2_ and 0.5 mM AcCoA at 37 °C for 12 h. We could not observe the formation of UDP-GlcNAc, thus ruling out the possibility that KasF primes the KSM biosynthesis (Figure S18). The origin of UDP-GlcNH_2_ or UDP-GlcNAc may be directly from the first metabolism, as they are fundamental metabolites. To test whether KasF carries out the deacetylation reaction in the KSM biosynthesis pathway (given UDP-GlcNAc the precursor, a deacetylase is required but not found in the KSM BGCs), KasF was incubated with 1 mM UDP-GlcNAc and 0.1 mM divalent ion (Zn^2+^). We found that neither KasF nor KasH was able to catalyze the deacetylation reaction (Figure S19).

**Figure 5:**
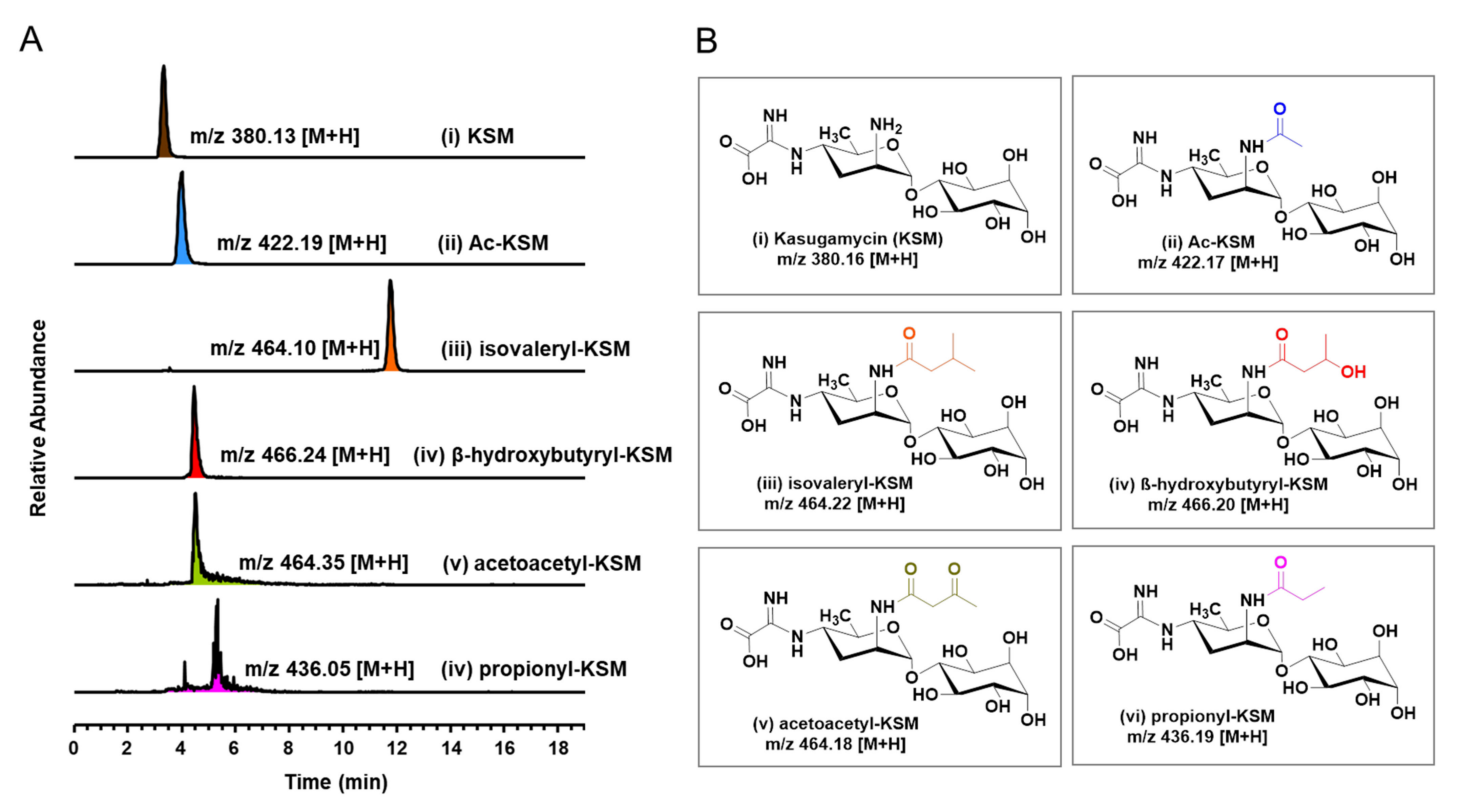
HPLC-LTQ-MS analysis for 2-N’-acyl substituted KSM analogs generated using KasH. (**A**) EIC spectra of (i) KSM at *m*/*z* 380.13 [M+H], (ii) Ac-KSM at *m*/*z* 422.19 [M+H], (iii) isovaleryl-KSM at *m*/*z* 464.10 [M+H], (iv) β-hydroxybutyryl-KSM at *m*/*z* 466.24 [M+H], (v) acetoacetyl-KSM at *m*/*z* 464.35 [M+H], (vi) propionyl-KSM at *m*/*z* 436.05 [M+H]. (**B**) Chemical structures of 2-N’-acyl substituted KSM analogs with calculated *m*/*z* [M+H].

A number of studies revealed that modified aminoglycoside antibiotics carried encouraging antimicrobial activity to inhibit some human pathogens [29,30,31,32]. For example, the enzymatically modified isepamicin (ISP), 6′-N-acetylated ISP analogs, manifested stronger antimicrobial activity but less cytotoxicity than ISP against ISP-resistant Gram-negative pathogens [33]. This fact prompted us to examine KasH for its ability to generate 2-N’-acylated KSM analogs. KasH was examined in the presence of AcCoA, isovaleryl-CoA, β-hydroxybutyryl-CoA, acetoacetyl-CoA, and propionyl-CoA alongside KSM, whereby new 2-N’-acyl substituted KSM analogs were generated (Figure 5). The binding affinity of KasH versus isovaleryl-CoA (*K*_d_ = 199.2 µM), acetoacetyl-CoA (*K*_d_ = 98.0 µM), and propionyl-CoA (*K*_d_ = 35.0 µM) was further determined (Figure S17 and Table S1), indicating that KasH has considerable substrate promiscuity when it comes to forming new analogs.

### 3.3. Crystal Structure of KasQ in Complex with UDP and UDP-Glc

**Figure 6:**
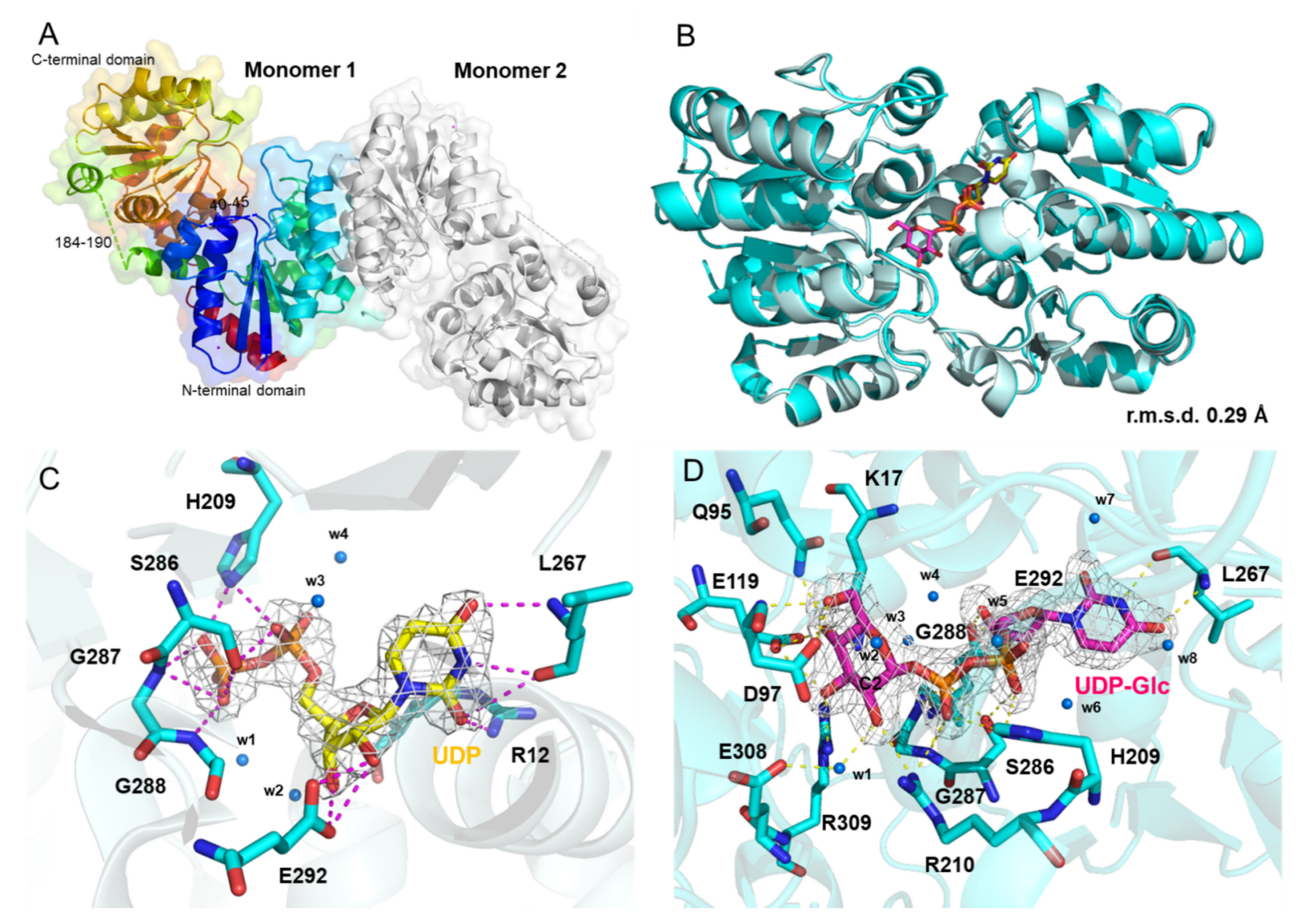
Crystal structures of KasQ in complex with UDP or UDP-Glc. (**A**) KasQ–WT structure. Monomer 1 is displayed in rainbow colors (N-terminal blue/C-terminal domain in red) and monomer 2 is displayed in gray. The missing residues 40–45 and 184–190 are designated by dashed lines. (**B**) Superimposition of the KasQ–UDP monomer (pale cyan) with the KasQ–UDP-Glc monomer (cyan) gives an RMSD of 0.29 Å. UDP and UDP-GlcNAc are displayed as yellow and pink sticks, respectively. (**C**) The UDP model fits well into its 2F_o_-F_c_ electron density map (contoured at 1 σ). (**D**) The UDP-Glc model fits well in its 2F_o_-F_c_ electron density map (contoured at 1 σ).

To better understand the catalytic mechanism of KasQ, we solved crystal structures of KasQ for the wild type (WT) and its complexes with UDP or UDP-Glc in resolutions of 2.44 Å, 2.58 Å, and 2.09 Å, respectively. Concerning the structure in complex with UDP-GlcNAc—the structure that was most wanted—it was not obtainable from all our co-crystallization conditions. Despite this, the structures, not least the one with UDP-Glc, are still in a position to provide a structural basis to help elucidate the mechanism of the epimerization reaction mediated by KasQ. Figure 6A and Figure S20, respectively show the ribbon diagram of the tertiary structure and the topology diagram of the secondary structure of KasQ-WT. In consistence with homodimers in solution, an asymmetric unit contains two monomers colored in rainbow (N-terminal blue, C-terminal red) and gray. Superimposition of these two monomers with an RMSD of 0.86 Å for 317 Cα atoms indicated no significant conformational differences. In brief, each monomer consists of two domains (N-terminal and C-terminal domains), in which each subdomain is made of a typical sandwich (αβα) architecture of a Rossmann fold (Figure 6A). Two fragments marked in dashed lines (residues 40–45 in the N-terminal domain and residues 184–190 in the C-terminal domain) are missing from the model due to lack of electron density, likely a consequence of structural disorder/flexibility in loop regions. The N-terminal domain is constituted by residues 1–170 that fold into a central seven-strand β-sheet (β1–β7) sandwiched by α-helices (α1–α7) on each side along with a stand-alone α16 helix (residues 357–370). The C-terminal domain is formed by residues 201–359 composed of a central six-strand β-sheet (β8–β13) sandwiched α-helices (α9–α16) on each side. The N-terminal and C-terminal domains are connected by two helices, α7–α8 (residues 173–199). The cleft between these two domains provides the substrate binding pocket.

As revealed above, UDP or UDP-Glc is bound to its binding site at the interface of the two domains. The superimposition of KasQ–UDP and KasQ–UDP-Glc with an RMSD of 0.29 Å for 332 Cα atoms suggested no apparent conformational differences as a result of binding from different ligands (Figure 6B). The electron density of UDP or UDP-Glc is well defined and consistent in the substrate binding site (Figure 6C,D). The UDP binding site is highly conserved among all non-hydrolyzing UDP-GlcNAc 2-epimerases (Figure S21): the guanidino side chain of Arg12 and the backbone of Leu267 are in close contact with the uracil O2 atom, likely due to hydrogen bonding (3.1 Å and 3.4 Å, respectively). The backbone of Leu267 is also in a hydrogen-bonding distance to the O4 atom (3.1 Å) and the N3 atom (2.7 Å) of the uracil base. The two hydroxyl groups of the ribose moiety are associated with Glu292 (2.8 Å and 2.6 Å) by hydrogen bonds. His209 may play a role in stabilizing UDP by interacting with its α-phosphate (3.1 Å) and β-phosphate (2.6 Å). Ser286, Gly287, and Gly289 are three highly conserved residues for molecular recognition, in which Ser286 interacts with α/β phosphate (2.7 Å/3.2 Å, respectively), Gly287 is in contact with the O1/O2 atom of β-phosphate (3.3 Å/3.1 Å, respectively), and Gly289 is associated with the O1 atom of β-phosphate (2.9 Å) (Figure 6C).

In view of the KasQ–UDP-Glc complex, the UDP portion is well defined as it is surrounded by an array of highly conserved residues, His209, Leu267, Ser286, Gly287, and Gly289. The hexose moiety is held in place by a group of highly conserved residues for its recognition and epimerization, except for Arg12, which differs from the corresponding residues in other homologs pointing away from the uracil O2 atom (4.6 Å); the guanidino group of Arg309, which is associated with Glu291 (2.9 Å), is in close proximity to the C2 hydroxyl group (4.1 Å), and the terminal carboxyl group of Asp97 is in close proximity to the C2 atom (3.8 Å). These residues may act as a set of the interlinking acids/bases triggering the elimination/addition reaction for the epimerization of UDP-GlcNAc to UDP-ManNAc. The C3 hydroxyl group of the hexose moiety is also within hydrogen-bonding distances to both the guanidino group of Arg309 (2.7 Å/2.9 Å) and the carboxyl group of Glu119 (3.3 Å). The C4 hydroxyl group is interacting with both Lys17 and Gln95 through hydrogen bonding (3.5 Å and 3.1 Å, respectively). The C6 hydroxyl group is likewise interacting with the carboxyl and amino groups of Asp97 through hydrogen bonding (2.5 Å and 2.8 Å, respectively). Importantly, the carboxyl group of Asp97 that is located on top of the sugar plane relative to the C2 atom (3.8 Å) likely serves as the general base to deprotonate C2 in the first half of the reaction, yielding AAG and UDP; the Arg309/Glu291 dyad, on the other hand, acts as the general acid, likely protonating AAG to afford UDP-ManNAc, thus completing the epimerization reaction (Figure 6D). Samuel et al. created five mutants of catalysis-related residues K15A, D95Q, E117Q, E131Q, and H213N to evaluate their biochemical importance [34]: they observed that all the mutants can release the AAG intermediate. The activity of mutants D95Q and E131Q was 100-fold slower than that of the wild type. Although non-hydrolyzing UDP-GlcNAc 2-epimerases have been extensively studied, the catalytic residues that engage in the *anti*-elimination and *syn*-addition to form the product remain unclear. On the basis of the KasQ structural complexes, we highlighted the importance of two residues, Q95 and E308, which were mentioned less frequently in all UDP-GlcNAc 2-epimerases reported thus far (Figure S21). To confirm their importance, we prepared four mutants, Q95A, Q95E, E308A and E308Q, and performed biochemical assays by incubating them with UDP-GlcNAc and MgCl_2_ at 37 °C for 12 h. For mutants E308A and E308Q, both HPLC and TLC analyses did not show the appearance of the intermediates, thereby confirming their importance in the catalysis (Figure S22). Moreover, given their special and geographic position relative to the AAG intermediate, we propose that Asp97 acts as the active site general base to trigger the elimination reaction, resulting in the formation of UDP and AAG. In contrast, both mutants Q95A and Q95E performed equally well, as did the wild type in terms of biochemical reactions, thus negating their direct participation in the epimerization reactions. To this end, obtaining the UDP-Glc or UDP bound complexes along with the mutagenesis studies allowed us to summarize the catalytic mechanism of KasQ: Asp97 may serve as the first base to abstract the C2 proton from GlcNAc, resulting in the formation of AAG and the neglect of UDP that is stabilized by nearby Arg210. Since the *anti*-elimination and *syn*-addition follow different electron-configuration courses, an oxocarbenium intermediate may be transiently present for buffering the transition state as well as helping change the reaction path. The positively charged oxocarbenium intermediate may be stabilized by the negatively charged UDP nearby [35]. In the UDP-addition course, the Arg309/Glu291 dyad opposite to Asp97 may serve as the general acid to protonate AAG so that UDP-ManNAc is derived in a *syn*-addition manner, completing the epimerization reaction.

### 3.4. Structural Comparison

**Figure 7:**
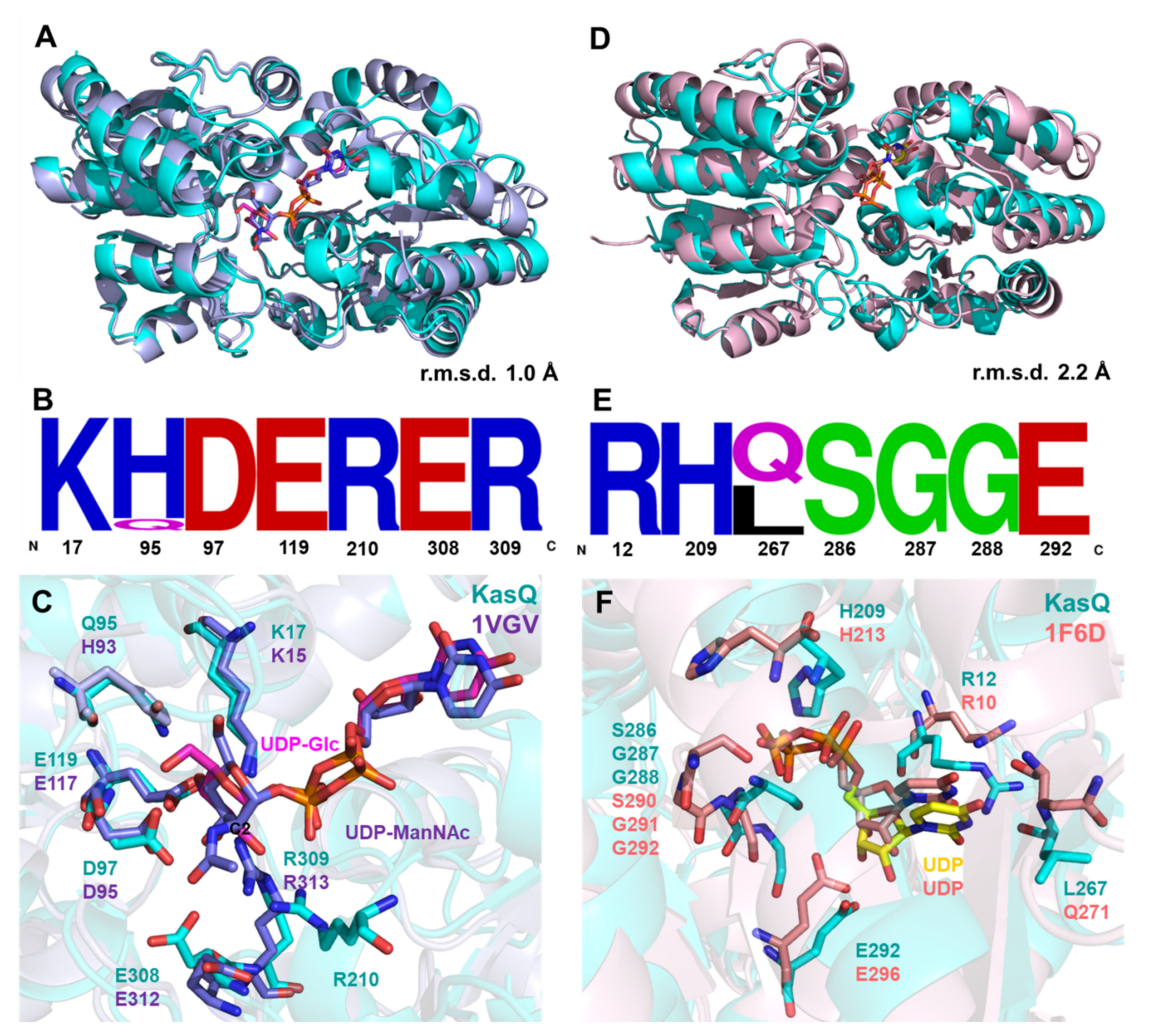
Sequence and structural comparison of KasQ with reported UDP-GlcNAc 2-epimerases. (**A**) Superimposition of KasQ–UDP-Glc monomer (cyan) with UDP-GlcNAc 2-epimerases of *E. coli* bound with UDP-ManNAc colored blue (PDB entry 1vgv, crystal structure modeled with UDP-ManNAc, mistakenly named UDP-GlcNAc) gives an RMSD of 1.0 Å. UDP-Glc and UDP-GlcNAc are colored as pink and blue sticks, respectively. (**B**) Sequence logo of sugar binding-site residues amid KasQ homologs. (**C**) The active site of KasQ–UDP-Glc superimposed with that of *E. coli* bound with UDP-ManNAc. (**D**) Superimposition of the KasQ–UDP monomer (cyan) with the *E. coli* counterpart bound with UDP (PDB entry 16fd) gives an RMSD of 2.2 Å. (**E**) Sequence logo of UDP binding sites amid KasQ homologs. (**F**) Active sites of KasQ–UDP superimposed with the *E. coli* counterpart bound with UDP.

The primary sequence and tertial structural analysis revealed that KasQ is a homolog to many non-hydrolyzing UDP-GlcNAc 2-epimerases in bacteria (Figure S21), for example, 2-epimerase from *E. coli* (PDB entry 1vgv; 47% identity [19]), *Neisseria meningitidis* (PDB entry 6vlc; 44% identity [36]), *Burkholderia vietnamiensis* (PDB entry 5dld; 44% identity), and *Bacillus subtilis* (PDB entry 4fkz; 42% identity). Despite the fact that KasQ exists as dimers in solution as well as asymmetric units, only one UDP-Glc molecule is bound to one protomer of the dimer. The superimposition of the KasQ–UDP-Glc complex with that from *E. coli* (PDB entry 1vgv) shows high structural similarity (RMSD of 1.0 Å for 286 Cα), UDP-glucose and UDP-ManNAc well overlapping each other in the active site, suggesting that both substrate-binding environments are comparable (Figure 7A). In light of the residue sequence of the substrate/active site of KasQ, it is highly conserved except for Q95 when aligned against other homologs (Figure 7B). Q95 was found interacting with the C4-OH of UDP-Glc (Figure 6D) in contrast to the histidine (H) counterpart in homologous 2-epimerases, which is at a distance from C4-OH of 4.5 Å. The close-up view out of these active sites suggests that there is adequate space for KasQ to accommodate UDP-GlcNAc or UDP-ManNAc (Figure S23). Residue R214 of that in *E. coli* is missing due to a lack of electron density (Figure 7C), while both structures fit well (*E. coli* epimerase bound with UDP, PDB entry 1f6d; 47% identity [37]) with an RMSD as low as 2.0 Å for 298 Cα (Figure 7D). The UDP binding sites among all the selected epimerases are conserved (Figure 7E) where residues SGG are highly conserved, likely to stabilize UDP by interacting with its α/β-phosphates through hydrogen bonds (Figure 7F).

A sodium ion was observed in coordination with the carbonyl backbone of N-terminal residues Leu25, Asp26, Asp28, and Phe31 (Figure S24). In the KasQ–UDP complex, the carboxyl group of Glu32 makes an additional interaction with the Na^+^ ion. Moreover, we found that the C-terminal residues Pro298, Ser250, and Ala352 of a KasQ homolog (PDB entry 1f6d) are associated with a Na^+^ ion (Figure S24D). These metal-ion binding sites, however, are ~25 Å away from the substrate binding region (Figure S24E), suggesting that they have little or no influences on the substrate-binding and enzymatic activity (Figure S24F).

### 3.5. KasQ Substrate Specificity

To investigate the substrate specificity, KasQ was assayed against an array of NDP-sugars. In brief, KasQ was incubated with NDP-sugars (UDP-GlcNAc, UDP-GalNAc, UDP-Glc, TDP-Glc, GDP-Glc, and UDP-Gal) in the presence of MgCl_2_ at 37 °C for 12 h and then subjected to HPLC analysis. In comparison with standards (both retention time and molecular weight, *m*/*z*), we concluded that KasQ can only convert UDP-GlcNAc **1** to UDP-ManNAc **4**. The consumption of a considerable amount of UDP-GlcNAc and release of AAG **2** and UDP **3** in solution were observed in a 12 h reaction. The fact is that none of the other NDP-sugars can be hydrolyzed by KasQ, suggesting that UDP-GlcNAc is the only valid substrate (Figure S25). The kinetic parameters for KasQ in the presence of UDP-GlcNAc and MgCl_2_ were determined: *k*_cat_ 1.33 s^−1^, *K*_M_ 40.28 mM, and *k*_cat_/*K*_M_ = 0.032 s^−1^mM^−1^ (Figure S26 and Table S2). In addition, the dissociation constants (*K*_d_) of KasQ versus nucleosides, (mono/di/tri) phosphates, NDP-sugars, and monosaccharides were examined using ITC, as summarized in Table 4 and Figures S27–S29. Neither UDP-GlcNH_2_ that was enzymatically synthesized using the published procedure [38] nor AMP, ADP, ATP, GMP, GDP, or GTP shows detectable binding affinity with KasQ. We nevertheless observed some binding affinity in a micromolar range when KasQ was titrated with UMP (*K*_d_ = 123.9 µM), UDP (*K*_d_ = 32.2 µM), UTP (*K*_d_ = 71.4 µM), and TTP (*K*_d_ = 174.5 µM) (Table 4 and Figure S27). Of these, the binding affinity of UDP versus KasQ was shown to be 2–5-fold higher than that of the others, suggesting that UDP-sugar is most likely the physiological substrate for KasQ. In contrast, UDP-Glc showed the highest binding affinity (*K*_d_ = 9.5 µM) in contrast to UDP-GlcNAc, which, for some reason, could not be determined (Table 4 and Figure S28). We speculated that divalent ions (Mg^2+^) may influence the binding but cannot conclude this from the ITC assay. Interestingly, both mutants Q95A and Q95E showed high binding affinity versus UDP-GlcNAc (*K*_d_ = 45.2 µM and *K*_d_ = 14.2 µM, respectively) comparable to that of WT versus UDP-Glc (Figure S30 and Table S3). In addition, we performed time course assays for KasQ in the presence of UDP-Glc and MgCl_2_, while we could not observe the formation of 2-hydroxyglucal and UDP-mannose, thereby concluding that UDP-Glc is not a substrate (Figure S31). Given these facts, we summarized that Q95 interacts with the C4 hydroxyl group of UDP-Glc (Figure 6D), while mutants Q95A and Q95E are not able to bind with UDP-Gal (C4-axial hydroxyl group). We propose that Q95 may play a critical role in the control of the reaction rate, directionality, and product release. Our mutagenic study may have shed new light on this subtle regulation in KSM biosynthesis while the actuality may depend on future detailed kinetic and biochemical/biophysical examinations.

### 3.6. In Vivo Isotope Incorporation Analysis

**Figure 8:**
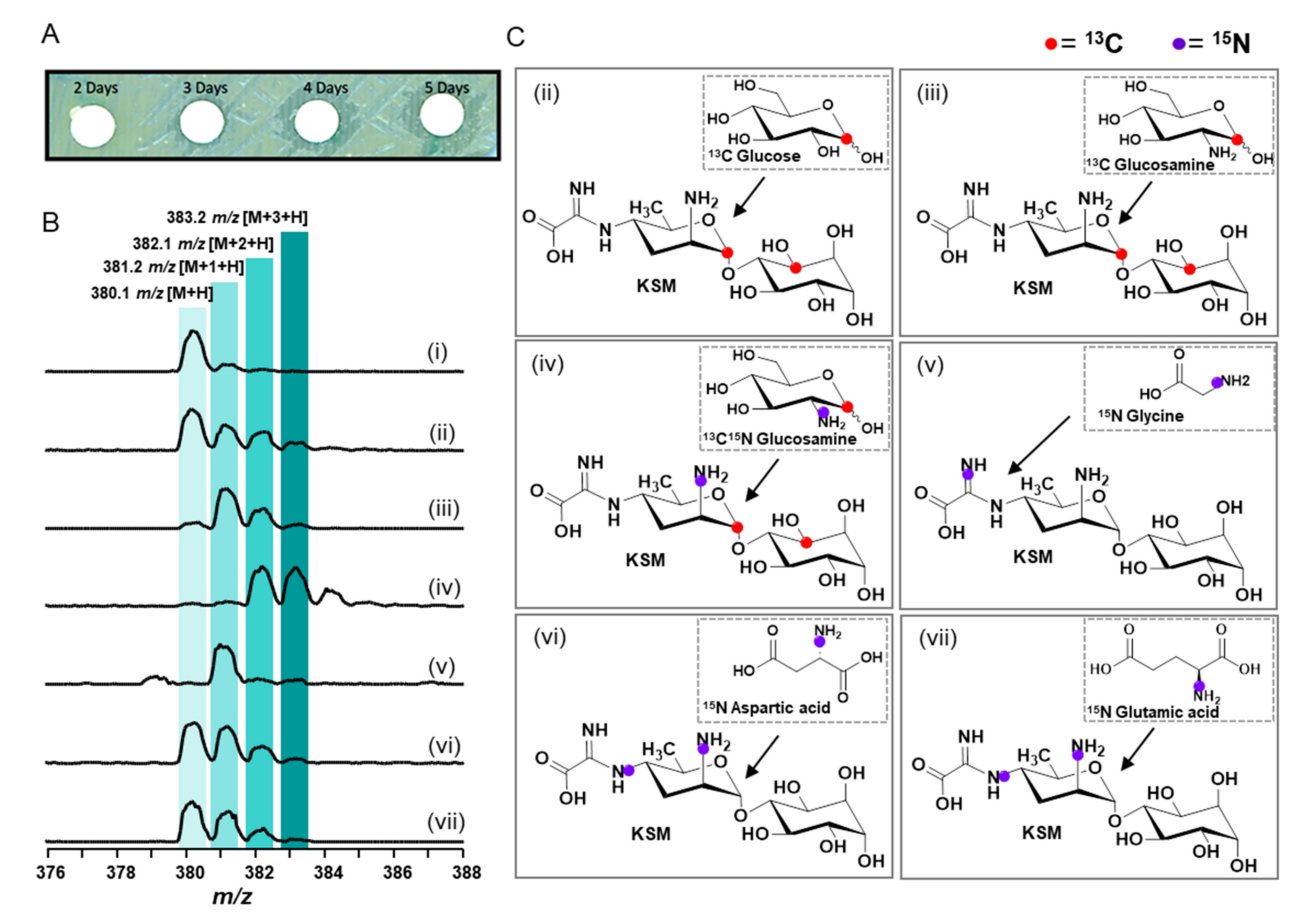
Disc diffusion and in vivo isotope incorporation assay. (**A**) Disc diffusion assay shows the zone of inhibition (ZOI) determining the optimal course of kasugamycin production against *E. coli*. (**B**) Isotope incorporation analysis: (i) KSM isolated from the producing strain fed without isotopes, (ii) KSM isolated from the producing strain fed with ^13^C1-glucose, (iii) KSM isolated from the producing strain fed with ^13^C1-glucosamine, (iv) KSM isolated from the producing strain fed with ^13^C1^15^N2-glucosamine, (v) KSM isolated from the producing strain fed with ^15^N-glycine, (vi) KSM isolated from the producing strain fed with ^15^N-aspartic acid, and (vii) KSM isolated from the producing strain fed with ^15^N-glutamic acid. Increments of *m*/*z* are colored in light to dark green. (**C**) Schematic diagram of isotope incorporation in KSM.

The origin of the main chemical components of KSM has previously been examined using isotopically labeled sugars, glycine, or ammonia. These results as a whole revealed that glucose or mannose is the precursor of kasugamine, that the D-inositol moiety is derived from *myo*-inositol, and that the glycine imine side chain comes directly from glycine [39,40,41,42,43]. This information, however, is insufficient to shed light on the real roles of KasF, H, and Q in the biosynthesis of KSM. To address the insufficiency, we used *Streptomyces lividans* TK64 harboring pMKBAC08-KAS as a model system to produce KSM, for which the susceptibility of *E. coli* to KSM was gauged by disc diffusion assay to profile its production. The inhibition zone appeared in agar plates on the third day, suggesting that KSM had been produced to a considerable quantity by manifesting a significant inhibition zone against *E. coli* (Figure 8A and Figure S33). Samples collected from the culture medium were subjected to HPLC-TQ-MS (positive mode), whereby the molecular weight of KSM was determined with *m*/*z* at 380.1 [M+H] as a reference. To see if KSM directly derived from glucose or glucosamine, we fed three precursors, ^13^C1-D-glucose, ^13^C1-D-glucosamine, and ^13^C1,^15^N2-D-glucosamine, to the culture of *Streptomyces lividans* TK64. We found that only ^13^C1-D-glucose is partially incorporated into KSM, as shown by the isotopic profile of KSM with *m*/*z* at 380.1 [M+H], 381.2 [M+1+H], and 382.1 [M+2+H], in which the latter two displayed a ratio close to unit, likely as a result of D-glucose that serves as the precursor for both kasugamine and inositol in the KSM biosynthetic pathway (Figure 8B, Figures S1 and S32). Given ^13^C1-D-glucosamine, the isotopic profile is dissimilar to ^13^C1-D-glucose, where it showed a dominant *m*/*z* at 381.2 [M+1+H] and a second dominant *m*/*z* at 382 [M+2+H] with a ratio close to 2 in contrast to the almost vanished native KSM (*m*/*z* at 380.1 [M+H]), suggesting that glucosamine instead of glucose is the main or direct precursor of kasugamine and is readily convertible back to glucose at a high rate, an alternative source to *myo*-inositol (Figure 8B and Figure S32). Given ^13^C1,^15^N2-D-glucosamine, the isotopic profile showed the dominant *m*/*z* at 382.2 [M+2+H] and 383 [M+3+H] with a ratio close to unit confirming the above reasoning. D-glucose is an indirect substrate, while glucosamine is the “direct” substrate in terms of utilization efficiency towards incorporation into KSM. On the other hand, given ^15^N-glycine, the molecular weight of KSM increased by one mass unit (*m*/*z* 381.2 [M+1+H]), indicating that glycine is the key precursor for the carboxyformidoyl group at C4-NH_2_ of KSM. Although UDP-N-Glc derivatives are the key precursors for KSM, the origin of the C4 amino group was parallelly investigated using ^15^N-aspartic acid or ^15^N-glutamic acid. An equal isotopic increment at M+H and M+1+H and a relatively minor extent of M+2+H for KSM were observed, suggesting that both ^15^N-aspartic acid and ^15^N-aspartic acid are the C4-NH_2_ donor of KSM and a minor C2-NH_2_ donor of glucosamine (Figure 8B,C). Based on our bioinformatics analysis, we put forward that KasC, the committed transaminase (28% similarity to PDB entry 5ghg, ω-Transaminase), may transfer the amino group given by either of these two amine donors to the precursor of KSM [44]; KasN, the committed glycine oxidase (42% similarity to fms14), may oxidatively add the glycine to the kasugamine precursor, forming carboxyformimidoyl-kasugamine [45].

## 4. Conclusions

In summary, KSM is produced by *Streptomyces kasugaensis*, isolated in 1965 [2]. It has been widely used in agriculture because of good activities against a plethora of plant bacterial diseases, for example, to control rice bacterial grain and seedling rot [4]. Beyond this, a number of new findings have highlighted that KSM is a promising drug lead, as it effectively counters some recalcitrant human diseases [5,6,7,8]. Unlike other aminoglycosides, KSM is composed of two unusual sugars, a D-*chiro*-inositol and a kasugamine with a glycine imine appendage. Whereas KSM has been discovered more than 50 years, its biosynthetic pathway remains still elusive due to lack of biochemical evidence. Based on the previous hypothesis, KasF was proposed as a starter enzyme involved in the formation of UDP-GlcNAc to initiate the biosynthesis of KSM, KasH as a self-resistance enzyme to regulate its biosynthesis, and KasQ as an enzyme standing in the middle of the pathway for the formation of kasugamine. In this study, we took advantage of in vitro biochemical and biophysical analyses alongside in vivo isotope feeding assays to solve these outstanding issues. First, we biochemically characterized the function of KasQ, which indeed is an epimerase converting UDP-GlcNAc to UDP-ManNAc, likely serving as the first step in the KSM biosynthesis. Mechanistically, we made use of NMR, stable isotope labelling, and synthetic AAG to ascertain that KasQ belongs to the un-hydrolyzing 2-epimerase protein family, in which the 2-epimerization reaction is carried out following the *anti*-elimination and *syn*-addition dual mechanisms. Structurally, we determined three crystal structures of KasQ for the wild type and its complex with UDP or UDP-Glc, thus allowing insights into the molecular recognition and catalytic mechanism at the molecular level. KasF was previously proposed as an enzyme that primes the biosynthesis of KSM; our in vivo and in vitro biochemical assays, however, ruled out this possibility. The fact is that UDP-GlcNH_2_ cannot be converted to UDP-GlcNAc in the presence of KasF alongside AcCoA. KasF, nevertheless, shares the KSM-inactivation activity to some extent. By contrast, KasH is literally the aminoglycoside-modifying enzyme transferring the acetyl group from AcCoA specific to KSM to form 2-N’-acetyl KSM. Finally, the isotope-feeding study confirmed that ^13^C,^15^N-glucosamine/UDP-GlcNH_2_ rather than glucose/UDP-Glc is the direct precursor in the formation of KSM. To this end, we have addressed several long-standing unresolved issues, which should be conducive toward mapping out the correct KSM biosynthetic pathway. The information garnered should also pave the way for developing novel KSM-based medicines using chemoenzymatic and synthetic biology approaches.

## Figures and Tables

**Table 1:** Summary of crystal data-collection statistics.

	KasQ–WT			KasQ–UDP			KasQ–UDP-Glc		
**PDB code**	7VYY			7VZA			7VZ6		
**Wavelength (Å)**	1.0			1.0			1.0		
**Space group**	C2			P1			P1		
**Cell dimensions**									
***a*, *b*, *c* (Å)**	112.4	106.1	94.3	81.8	104.7	106.3	82.8	104.9	106.8
***α*, *β*, *γ* (°)**	90	124.9	90	74.5	73.0	68.4	74.9	72.9	68.1
**Resolution (Å)**	25.45–2.44 (2.55–2.46)			19.89–2.60 (2.68–2.60)			21.56–2.10 (2.18–2.10)		
***R*_merge_**	0.031 (0.387)			0.023 (0.365)			0.035 (0.296)		
***I*/*I***	26.6 (2.4)			23.8 (1.9)			19.7 (2.7)		
**Completeness (%)**	85.9 (86)			93.9 (94.1)			95.2 (95.1)		
**Redundancy**	3.6 (3.9)			2.4 (2.5)			2.2 (2.1)		
**Refinement**									
**Resolution (Å)**	25.45–2.44 (2.55–2.44)			19.9–2.58 (2.68–2.58)			21.5–2.09 (2.14–2.09)		
**No. reflections**	28,957			91,395			176,474		
***R*_work_/*R*_free_**	0.22/0.27			0.21/0.24			0.20/0.22		
**R.m.s deviations**									
**Bond lengths (Å)**	0.66			0.66			0.70		
**Bond angles (°)**	0.77			0.79			0.84		

**Table 2:** The sequences of primers used in this study to create KasQ mutants.

Mutants	Primers
Q95A	Forward 5′-TGGTGTCTCCCGCTACCACGGCGACGTCC-3′ Reverse 5′-GGACGTCGCCGTGGTAGCGGGAGACACCA-3′
Q95E	Forward 5′-TGTCTCCCTCTACCACGGCGACGTCCA-3′ Reverse 5′-TGGACGTCGCCGTGGTAGAGGGAGACA-3′
E308A	Forward 5′-CCTCGGGGCGCGCCGTGCGGTCC-3′ Reverse 5′-GGACCGCACGGCGCGCCCCGAGG-3′
E308Q	Forward 5′-CTCGGGGCGCTGCGTGCGGTCCC-3′ Reverse 5′-GGGACCGCACGCAGCGCCCCGAG-3′

**Table 3:** The sequences of primers used to construct pMKBAC08-KAS and pWZC07-PrpsJ-kasT.

Primers	Sequence
P303 P304 P115 P116 P117 P118	TAACCGTTTAAACTTAATTAAGAAGATCCTTTGATCTTTTCTACGG AAGCCCTGGATCCAATTCCCCAATGTCAAGCACTT ATTATTATTCATATGCGCGTCATCGACGGGATGCACCGGCT ACCTGTGTTCAACCAAGGAATTTCCTGGGTTGAACACCGC AAGATCAGATCCCCGCGGTGACGTAGTACGAGAT ACAGCGTCATGACGTGAAGTTCAGCGACCCGT
P119 P120 P121 P122 P123 P124	ATTATTATTCAATTGCACTACAAGTTCCGCTGGGTGTACCTGAATTT ATTATTATTTTAATTAAGCTTATTCGCAGCAGTCCTCGATGACGAAGACA CAGGAAATTCGAGGCGGAATTCGCCGAATCCTT TGCCTTGTCGTCGTAGGTGTGGAGGCTGTT TTCGACATCGGCATGATCACCCACGGTCTGCTCT ATTATTATTAAGCTTAACAACGTGACGGGTTCAGCCAGTTACCGCTTCATGCTT

**Table 4:** Thermodynamic parameters of KasQ versus different substrates.

S.No	KasQ with	n	K_d_ (µM)	∆G (kcal mol^−1^)	∆H (kcal mol^−1^)	∆S (cal mol^−1^ K^−1^)
1	AMP	NBD	NBD	NBD	NBD	NBD
2	ADP	NBD	NBD	NBD	NBD	NBD
3	ATP	NBD	NBD	NBD	NBD	NBD
4	GMP	NBD	NBD	NBD	NBD	NBD
5	GDP	NBD	NBD	NBD	NBD	NBD
6	GTP	NBD	NBD	NBD	NBD	NBD
7	TTP	1.1 ± 0.1	174.5 ± 20.2	−5.128 ± 1.221	−7.612 ± 1.221	−8.33
8	UMP	1.0 ± 0.0	123.9 ± 70.9	−13.19 ± 1.245	−9.254 ± 1.245	13.2
9	UDP **3**	1.0 ± 0.0	32.2 ± 6.8	−6.128 ± 0.5523	−4.130 ± 0.5523	6.70
10	UTP	1.0 ± 0.0	71.4 ± 5.7	−5.645 ± 1.340	−9.104 ± 1.340	−11.6
11	UDP-GlcNAc **1**	NBD	NBD	NBD	NBD	NBD
12	UDP-GlcNAc/Mg^2+^	NBD	NBD	NBD	NBD	NBD
13	UDP-GalNAc	1.8 ± 0.4	334.4 ± 133. 7	−4.742 ± 1.700	−3.418 ± 1.700	4.44
14	UDP-Glc	0.5 ± 0.0	9.5 ± 0.3	−6.834 ± 0.0375	−0.3944 ± 0.0375	21.6
15	TDP-Glc	0.3 ± 0.0	25.1 ± 0.2	−6.273 ± 0.2773	−4.568 ± 0.2773	5.72
16	GDP-Glc	NBD	NBD	NBD	NBD	NBD
17	UDP-Gal	1.0 ± 0.2	273.3 ± 54.6	−4.862 ± 1.926	−6.636 ± 1.926	−5.95
18	UDP-GlcA	1.5 ± 0.2	76.3 ± 15.2	−5.61 ± 0.5751	−1.376 ± 0.5751	14.2
19	GDP-Man	NBD	NBD	NBD	NBD	NBD
20	UDP-GlcNH_2_	NBD	NBD	NBD	NBD	NBD
21	GlcNAc	NBD	NBD	NBD	NBD	NBD
22	GalNAc	NBD	NBD	NBD	NBD	NBD
23	GlcNH_2_	NBD	NBD	NBD	NBD	NBD
24	GlcA	NBD	NBD	NBD	NBD	NBD
25	GalA	NBD	NBD	NBD	NBD	NBD
26	D-Glucose	NBD	NBD	NBD	NBD	NBD
27	D-Mannose	NBD	NBD	NBD	NBD	NBD
28	D-Galactose	NBD	NBD	NBD	NBD	NBD
29	AAG **2**	NBD	NBD	NBD	NBD	NBD

NBD: No binding detected.

## Data Availability

All the data described in this article can be found in the text and supplementary information; protein data bank (PDB) ID: 7VYY, 7VZA and 7VZ6.

## Supplementary Materials

The following supporting information can be downloaded at: https://www.mdpi.com/article/10.3390/biomedicines10020212/s1, Figure S1: Previous biosynthetic proposals for kasugamycin (KSM), Figure S2: The proposed biosynthetic pathway of KSM from this study, Figure S3: Protein overexpression and purification for KasQ, KasF and KasH, Figure S4: The mass spectra (in m/z) of UDP-GlcNAc 1, UDP-ManNAc 4 and UDP 3 for the reactions carried out by KasQ in the presence of UDP-GlcNAc and MgCl_2_, Figure S5: The determination of the AAG 2 intermediate in time-course enzymatic reactions, Figure S6: Effects of different temperatures on the enzymatic activity of KasQ, Figure S7: The conversion rate of substrates to products, Figure S8: Trapping the intermediate and product formation by ^1^H NMR spectroscopy, Figure S9: Determination of deuterated water incorporated into UDP-GlcNAc, Figure S10: The mass spectra of UDP-GlcNAc incubated with D_2_O at different time points, Figure S11: The mass spectra of KasQ incubated with UDP-GlcNAc and MgCl_2_ in D_2_O at different time points, Figure S12: ^1^H NMR spectra, Figure S13: The sequence alignment of KasH and KasF, Figure S14: Disc diffusion assay of KasF and KasH with or without KSM, Figure S15: Disc diffusion assay of KasF and KasH with some commonly used aminoglycoside antibiotics, Figure S16: The mass spectra of the substrates and products (in m/z) of the KasF, Figure S17: Isothermal Titration Calorimetry (ITC) analysis, Figure S18: Acetylation assay of KasF with UDP-GlcNH_2_ and AcCoA, Figure S19: Deacetylation assay for KasH or KasF with UDP-GlcNAc and Zn^2+^, confirming that both enzymes are lack of the deacetylation activity, Figure S20: Topology diagram of the protein fold of the KasQ protomer, Figure S21: Sequence alignment of KasQ with different UDP-GlcNAc 2-epimerases, Figure S22: Biochemical assay of KasQ mutants E308A and E308Q, Figure S23: The active site and the binding pocket of KasQ, Figure S24: The metal-ion binding site of KasQ, Figure S25: Enzymatic reactions of KasQ with different NDP sugars, Figure S26: The kinetic curve of KasQ for the epimerization reactions, Figure S27: ITC analyses of KasQ with different nucleosides (mono/di/tri) phosphates, Figure S28: ITC analyses of KasQ with different NDP sugars, Figure S29: ITC analyses of KasQ with different monosaccharides, Figure S30: ITC analyses of KasQ mutants with different NDP sugars, Figure S31: ^1^H NMR spectra of KasQ in time course reactions, Figure S32: Relative intensity of isotopes in mass spectra from different labeled sugar donors in order to probe the correct kasugamycin biosynthetic pathway, Figure S33: Disc diffusion test and mass spectrum of KSM, Figure S34: ^1^H and ^13^C NMR spectra, Figure S35: ^1^H and ^13^C NMR spectra, Figure S36: ^1^H and ^13^C NMR spectra, Table S1: Thermodynamic parameters of KasH for its binding affinity with testing ligands, Table S2: Kinetics of KasQ_WT in reaction with UDP-GlcNAc and MgCl_2_, Table S3: Thermodynamic parameters of KasQ mutants in the binding affinity assay.
