# Supplementary material for: KasQ an Epimerase Primes the Biosynthesis of Aminoglycoside Antibiotic Kasugamycin and KasF/H Acetyltransferases Inactivate Its Activity
Source: Biomedicines. 2022 Jan 19;10(2):212. doi: 10.3390/biomedicines10020212 (PMC8869731; doi:10.3390/biomedicines10020212)
Supplement: Supplementary file 1 [file biomedicines-10-00212-s001.zip › biomedicines-1494370-supplementary.pdf]

Supporting information for

KasQ an Epimerase Primes the Biosynthesis of Aminoglycoside Antibiotic Kasugamycin and KasF/H Acetyltransferases Inactivate Its Activity

Rajesh Rattinam,[1,2,3] R. Sidick Basha,[1] Yung-Lin Wang,[1] Zhe-Chong Wang,[1] Ning-Shian Hsu,[1] Kuan-Hung Lin,[1] Saeid Malek Zadeh,[1,2,3] Kamal Adhikari,[1] Jin-Pin Lin,[1] Tsung-Lin Li[1,2,4]*

[1] Genomics Research Center, Academia Sinica, Taipei 115, Taiwan

[2] Chemical Biology and Molecular Biophysics Program, Taiwan International Graduate Program, Academia Sinica, Taipei 115, Taiwan

[3] Institute of Bioinformatics and Structural Biology, National Tsing Hua University, Hsinchu 300, Taiwan

[4] Biotechnology Center, National Chung Hsing University, Taichung City 402, Taiwan

[*] Correspondence: tlli@gate.sinica.edu.tw

This file includes

Figures S1to S36

Tables S1 to S3

Figures

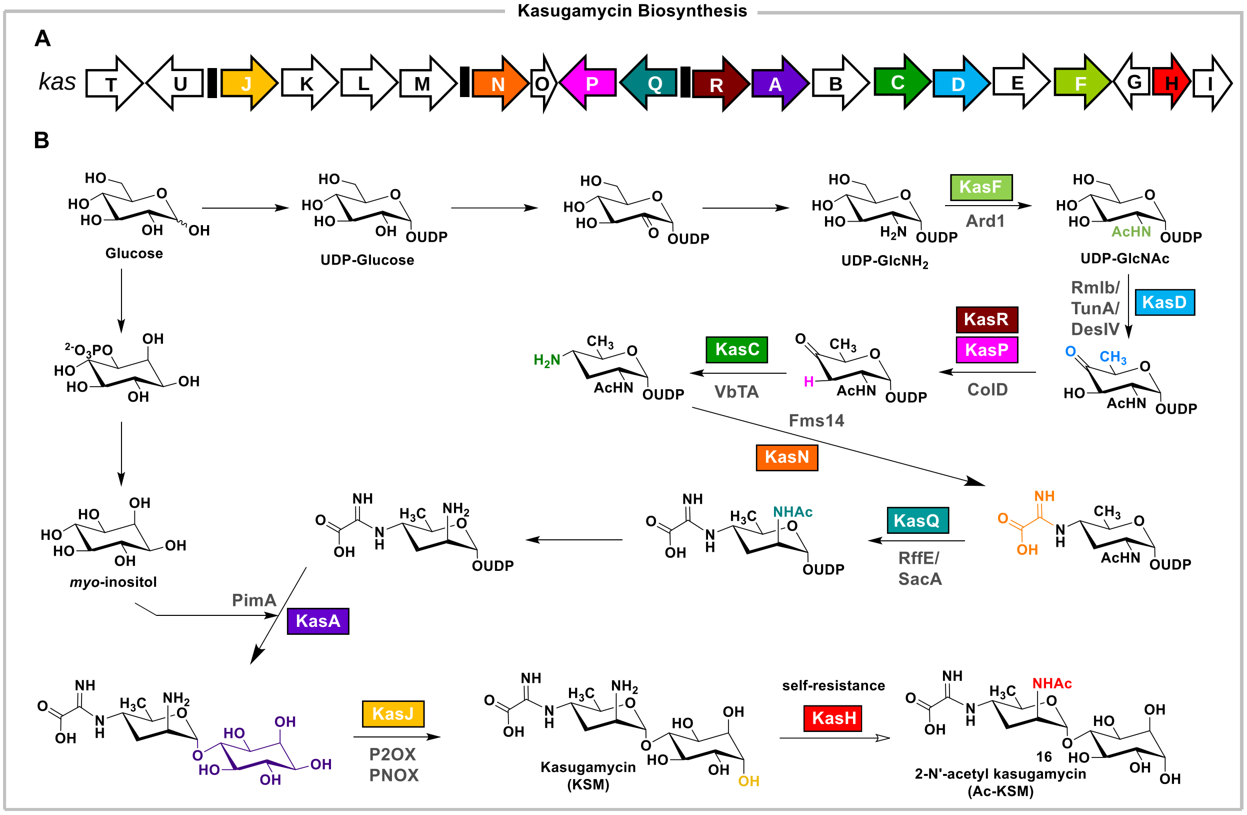

Figure S1. Previous biosynthetic proposals for kasugamycin (KSM). (A) The biosynthetic gene cluster identified from *Streptomyces kasugensis*. (B) Nine enzymes responsible for the KSM formation KSM. The self-resistance enzyme KasH that acetylates KSM to Ac-KSM is labeled in red color.

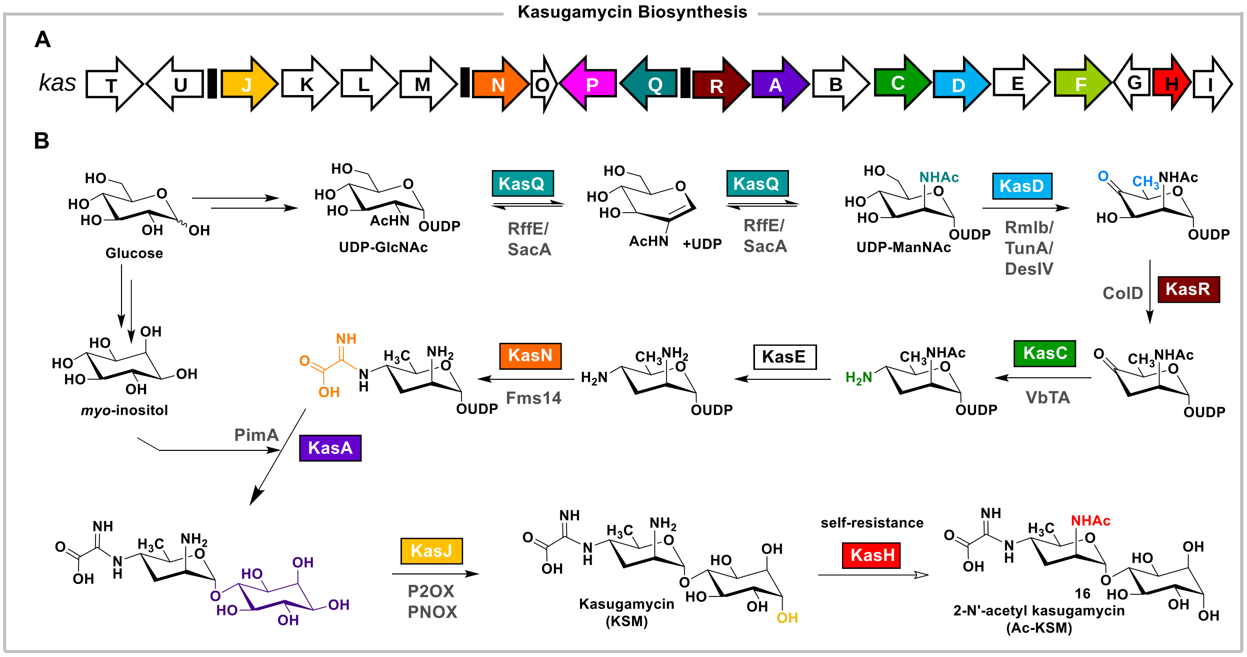

Figure S2. The proposed biosynthetic pathway of KSM from this study. (A) The biosynthetic gene cluster identified from *Streptomyces kasugensis*. (B) Based on biochemical assays, KasQ primes the KSM biosynthesis by converting UDP-GlcNAc to UDP-ManNAc. Seven downstream enzymes (KasD, KasR, KasC, KasE, KasN, KasA and KasJ) work sequentially transforming UDP-ManNAc all the way to the final product KSM.

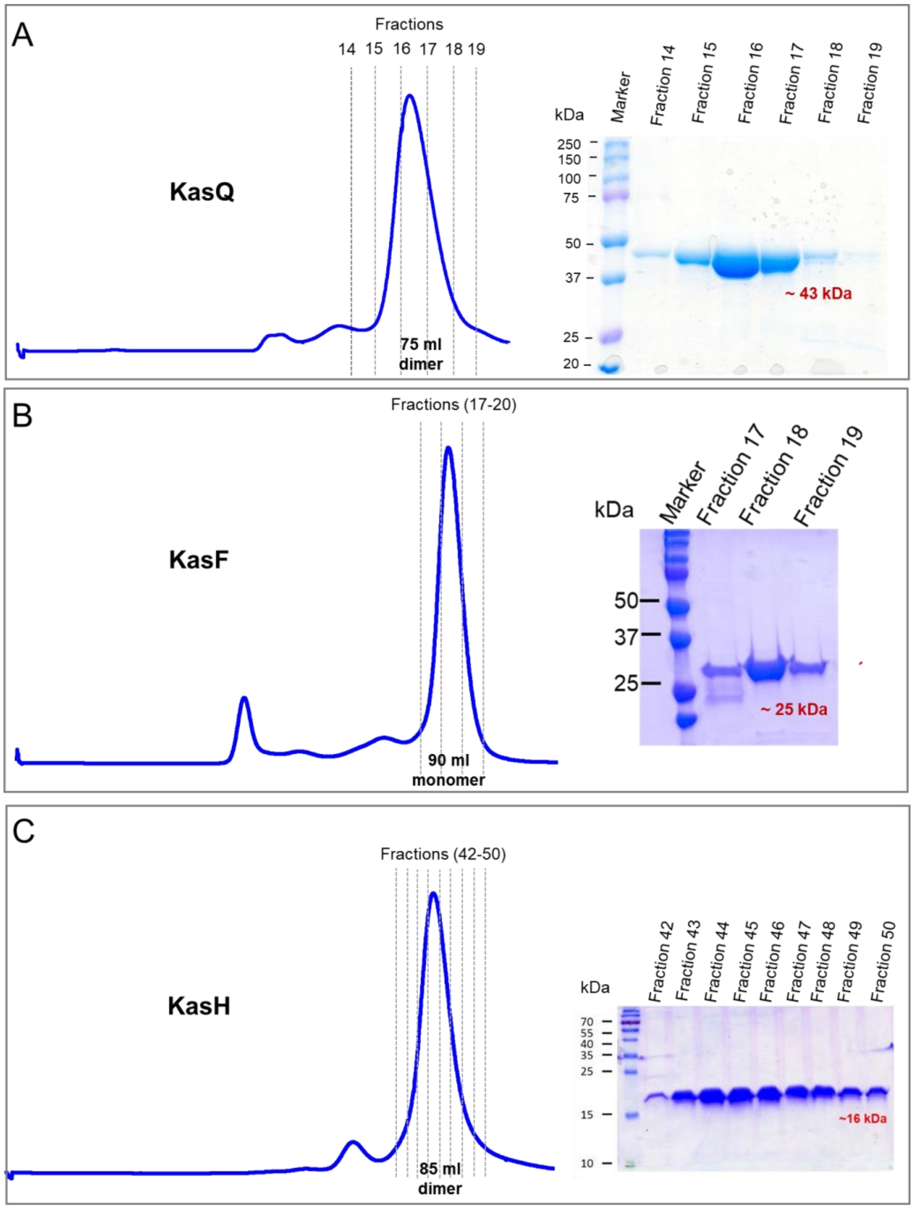

Figure S3. Protein overexpression and purification for KasQ, KasF and KasH. (A-C) Profiles from gel filtration chromatography (left) and SDS-PAGE (right).

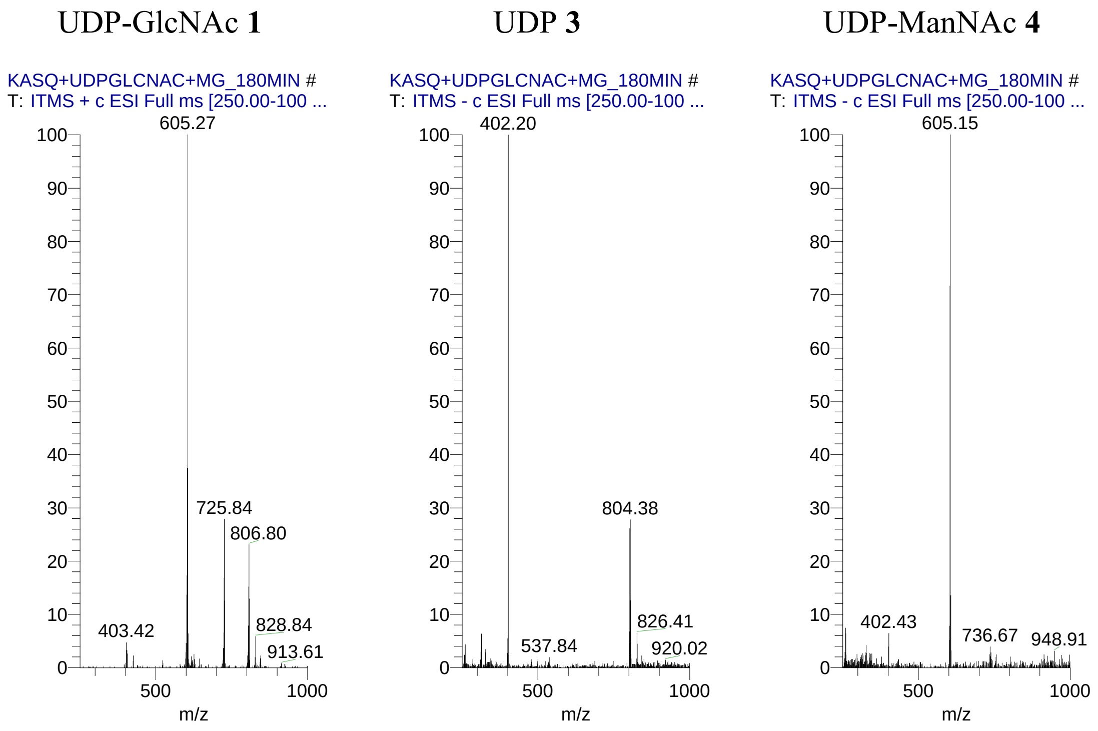

Figure S4. The mass spectra (in m/z) of UDP-GlcNAc **1**, UDP-ManNAc **4** and UDP **3** for the reactions carried out by KasQ in the presence of UDP-GlcNAc and $MgCl_2$.

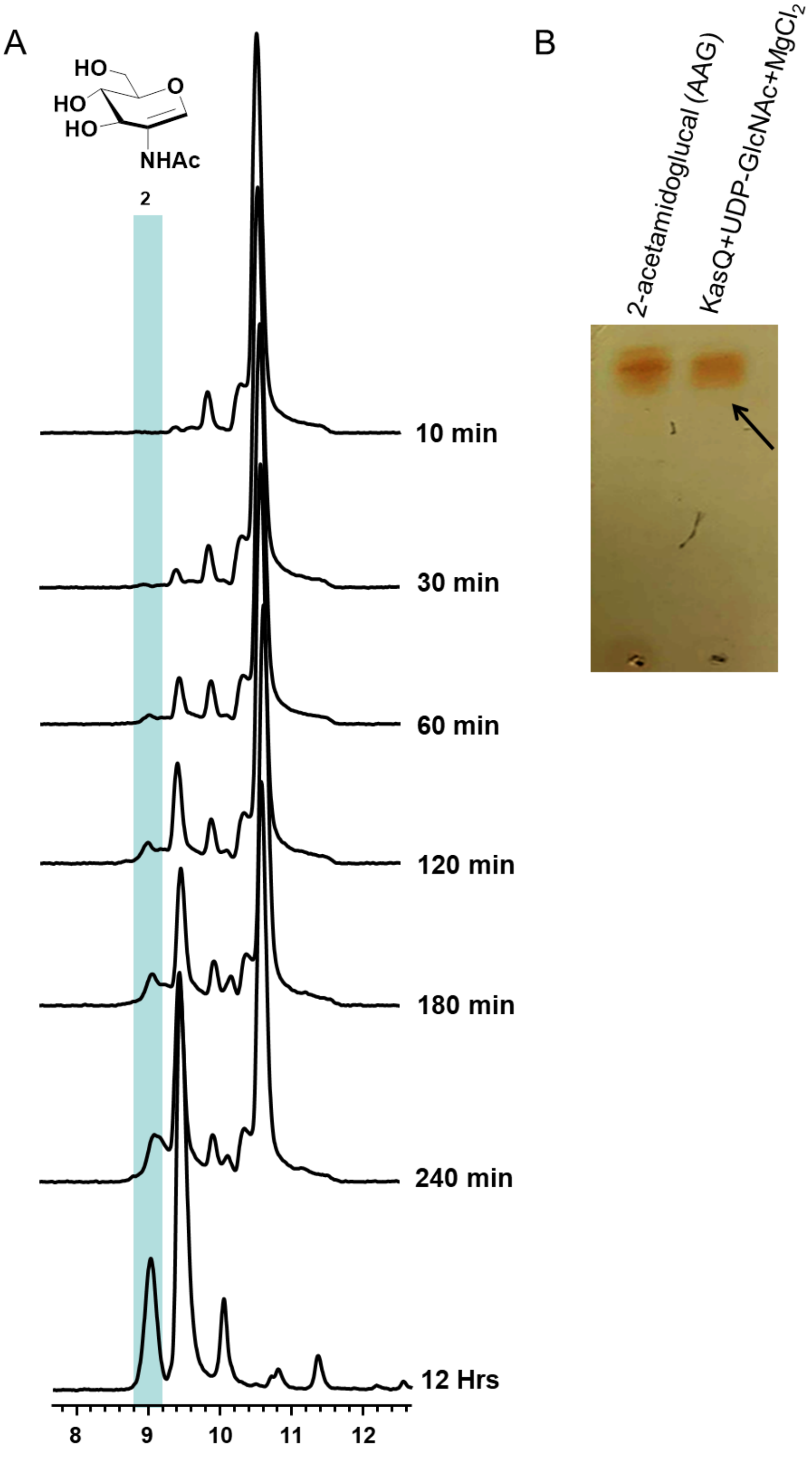

Figure S5. (A) The determination of the AAG **2** intermediate in time-course enzymatic reactions. (B) TLC analysis of synthetic 2-acetamidoglucal (AAG **2**) and the reaction mixture of KasQ with UDP-GlcNAc and $MgCl_2$. The arrow indicates that the AAG **2** intermediate is produced in the KasQ reactions.

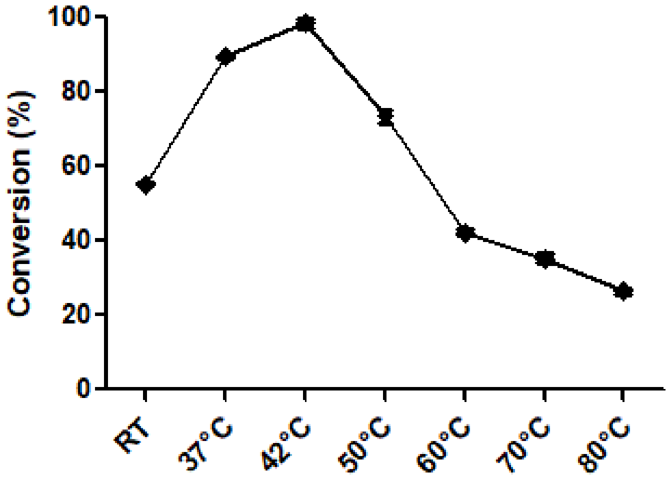

Figure S6. Effects of different temperatures on the enzymatic activity of KasQ.

A

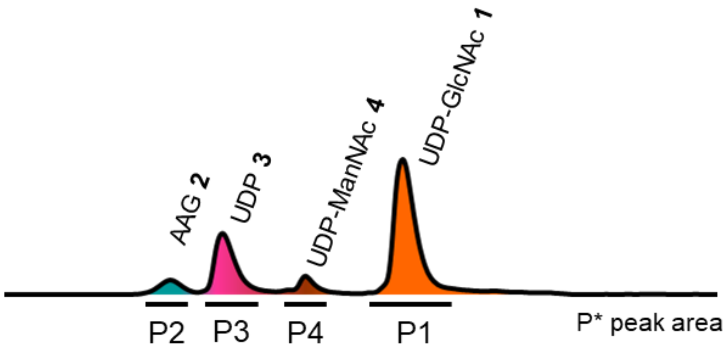

$$\text{Conversion \% of UDP-GlcNAc to UDP-ManNAc} = \frac{P4}{P1+P2+P3+P4} * 100$$

$$\text{Conversion \% of UDP-GlcNAc to UDP} = \frac{P3}{P1+P2+P3+P4} * 100$$

$$\text{Conversion \% of UDP-GlcNAc to AAG} = \frac{P2}{P1+P2+P3+P4} * 100$$

$$\text{Conversion \% of UDP-GlcNAc to product/intermediates} = \frac{P1}{P1+P2+P3+P4} * 100$$

B

Time (min)	UDP-GlcNAc 1 (%)	UDP-ManNAc 4 (%)	UDP 3 (%)	AAG 2 (%)
1. 10 min	91.8	6.8	1.1	0.2
2. 30 min	89.5	6.9	2.9	0.7
3. 60 min	85.4	6.2	6.9	1.5
4. 120 min	73.9	5.7	17.0	3.3
5. 180 min	69.1	5.3	21.0	4.5
6. 12 hrs	2.6	9.2	64.4	23.8

Figure S7. The conversion rate of substrates to products. (A) The equation for determining the conversion rate of UDP-GlcNAc **1** to UDP-ManNAc **4**, UDP **3** and AAG **2**. (B) The conversion rate of substrates to products/intermediates from 10 min to 12 hrs at different time points.

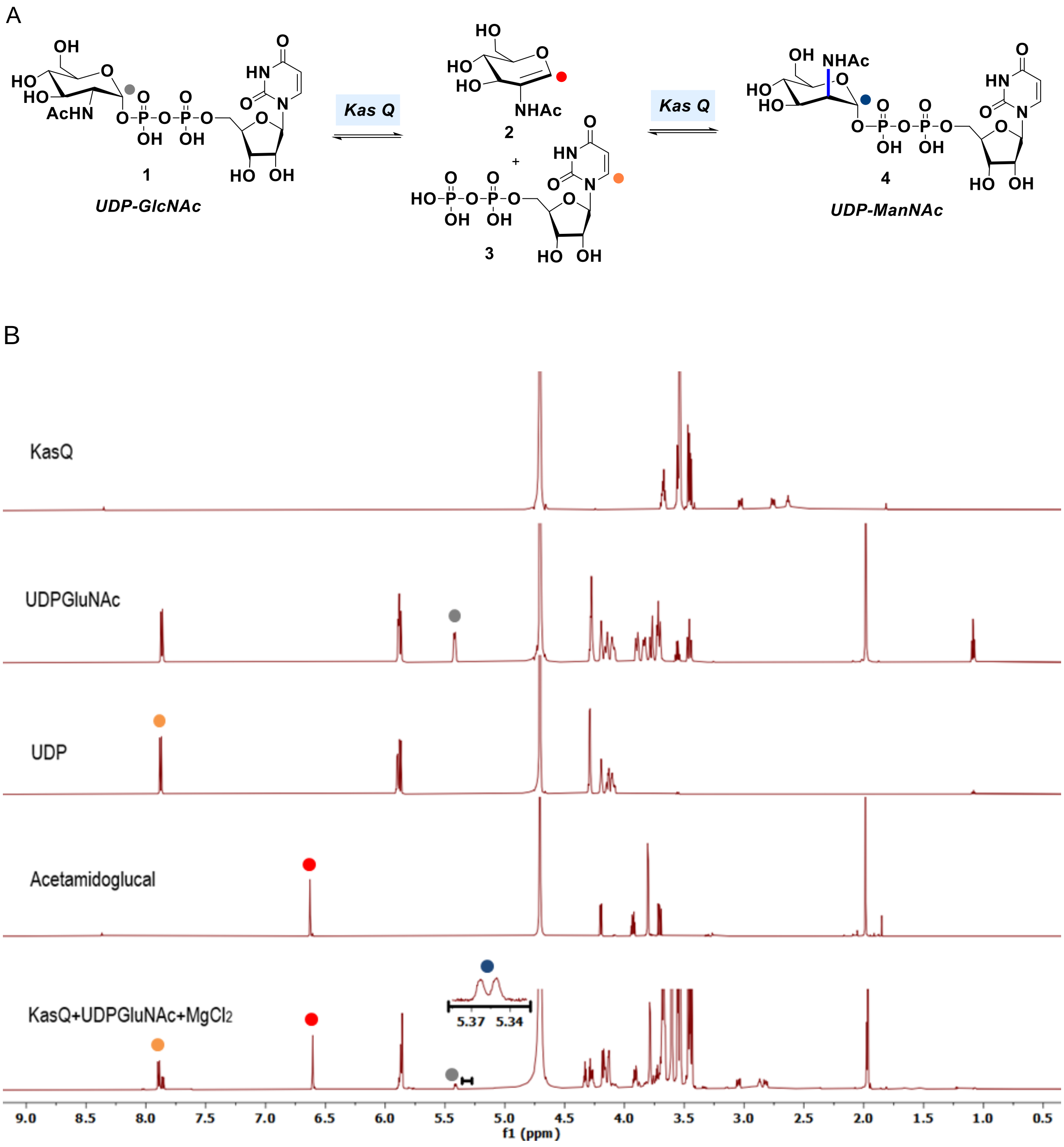

Figure S8. Trapping the intermediate and product formation by ^{1}H NMR spectroscopy. (A) Scheme of KasQ activity assay. The anomeric protons of UDP-GlcNAc **1** and UDP-ManNAc **4** were shown in gray and blue dots, respectively. For the intermediates, 2-acetamidoglucal H-1 proton was shown in red dot, and the proton corresponding to UDP **3** was shown in orange dot. (B) Stacking ^{1}H NMR spectrum of different standards and reaction mixtures.

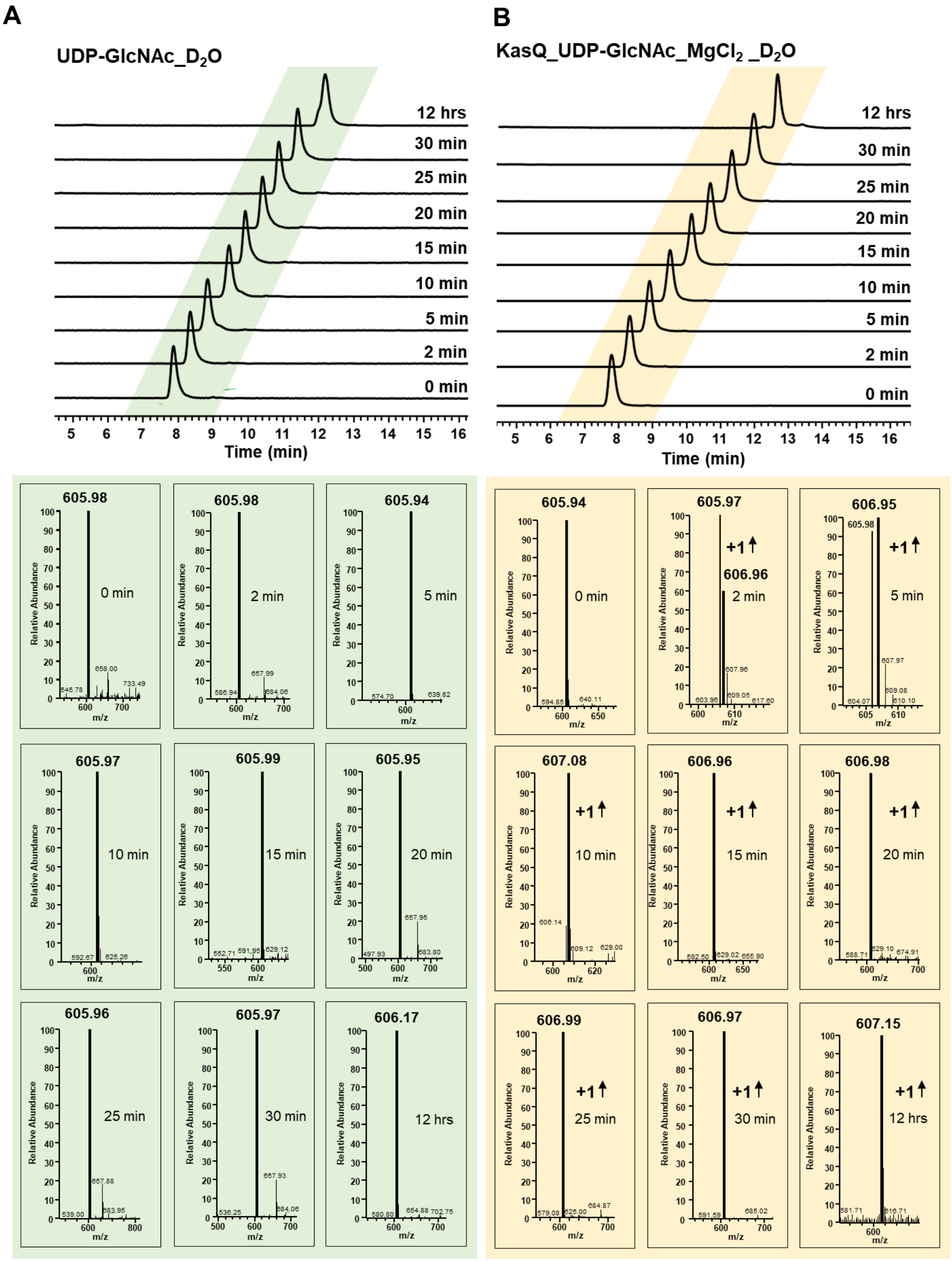

Figure S9. Determination of deuterated water incorporated into UDP-GlcNAc. (A) HPLC traces of incubated UDP-GlcNAc and $MgCl_2$ in D_2O at different time points. The corresponding molecular weight is shown on the bottom. (B) HPLC traces of KasQ incubated with UDP-GlcNAc and $MgCl_2$ in D_2O at different time points.

UDP-GlcNAc D_2O

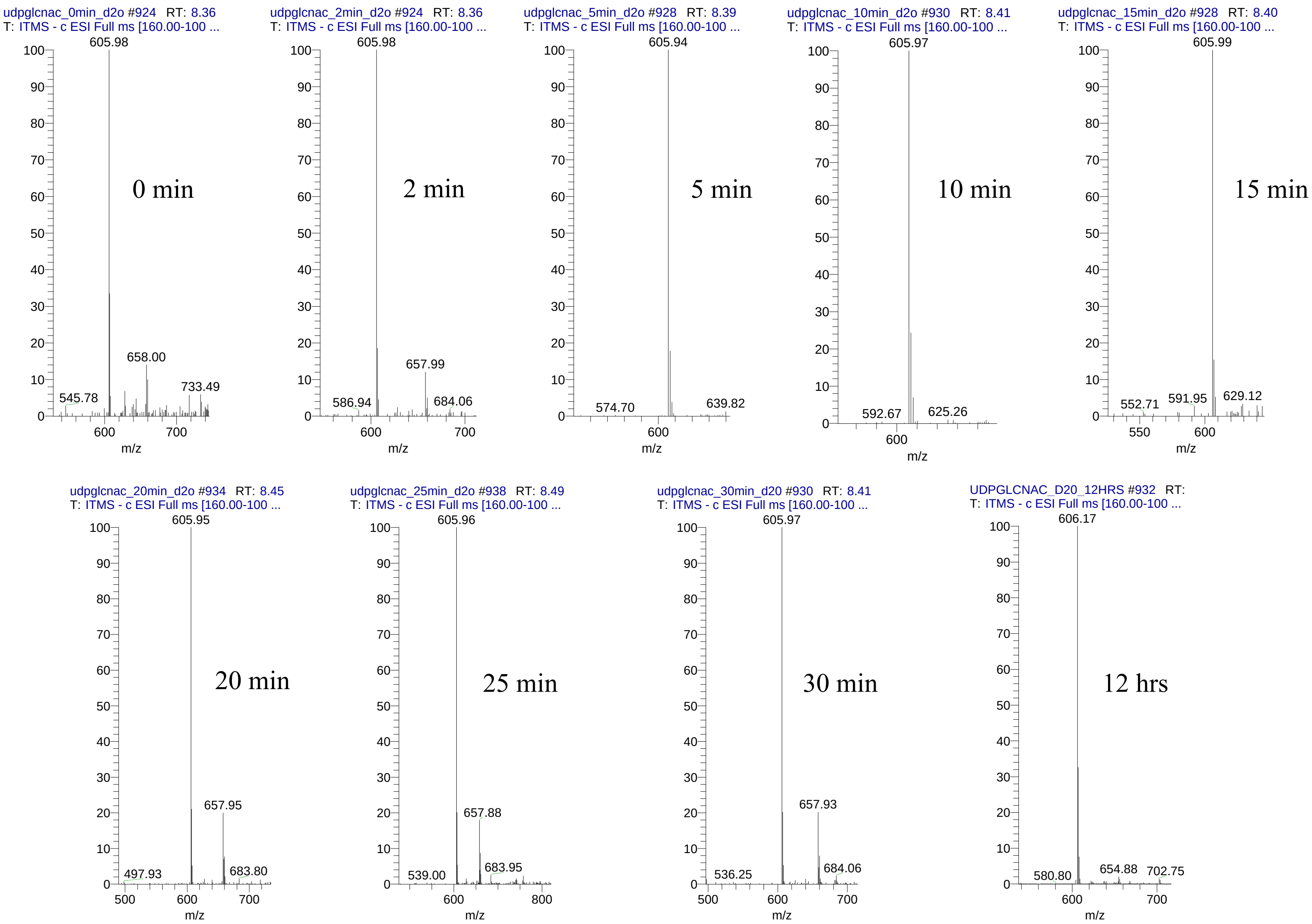

Figure S10. The mass spectra of UDP-GlcNAc incubated with D_2O at different time points.

KasQ UDP-GlcNAc $MgCl_2$ D_2O

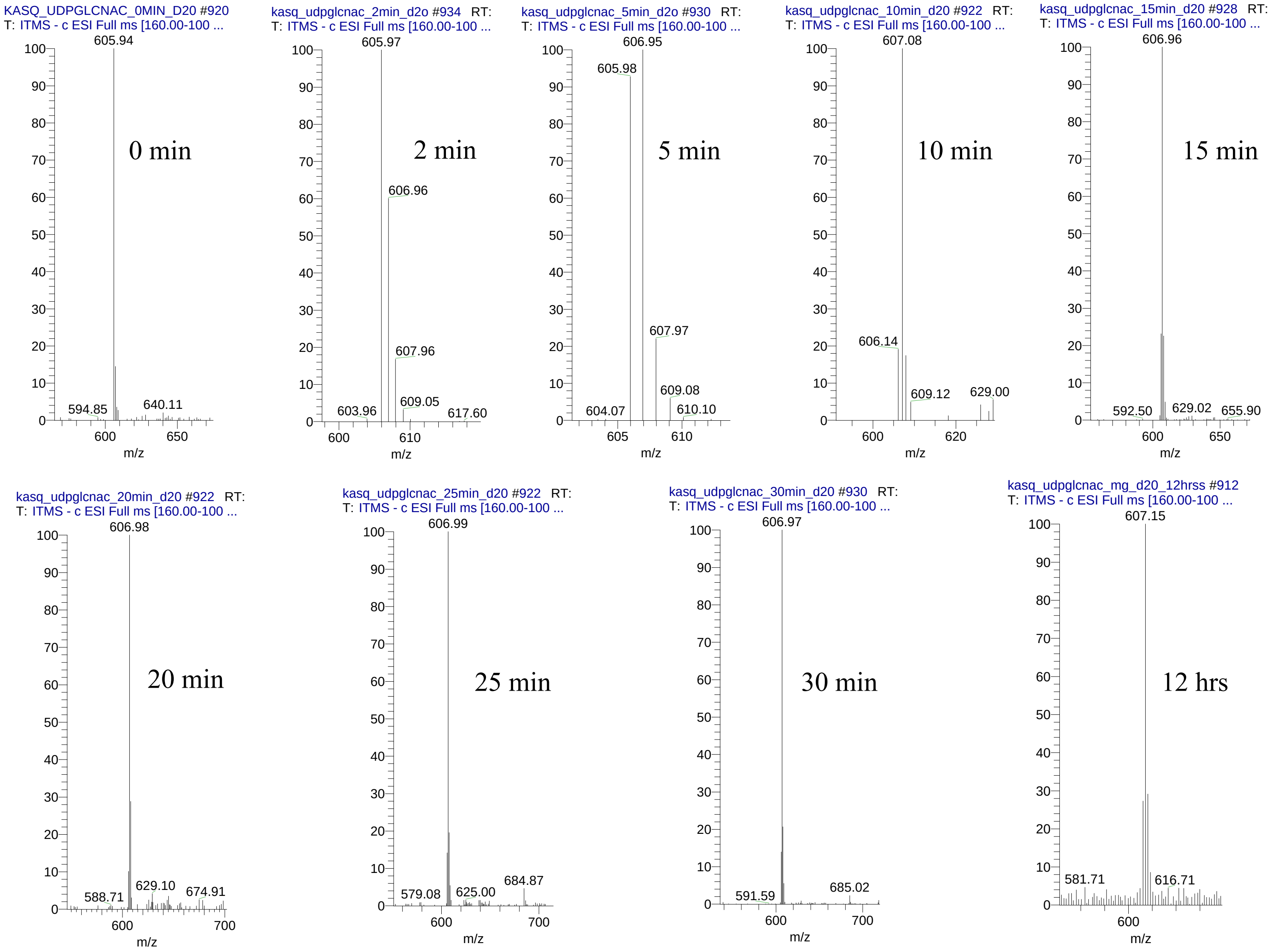

Figure S11. The mass spectra of KasQ incubated with UDP-GlcNAc and $MgCl_2$ in D_2O at different time points.

A

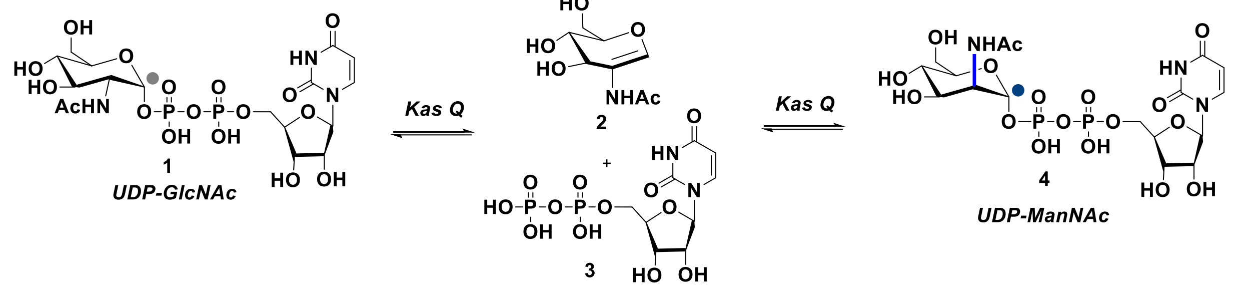

B

^{1}H NMR spectrum

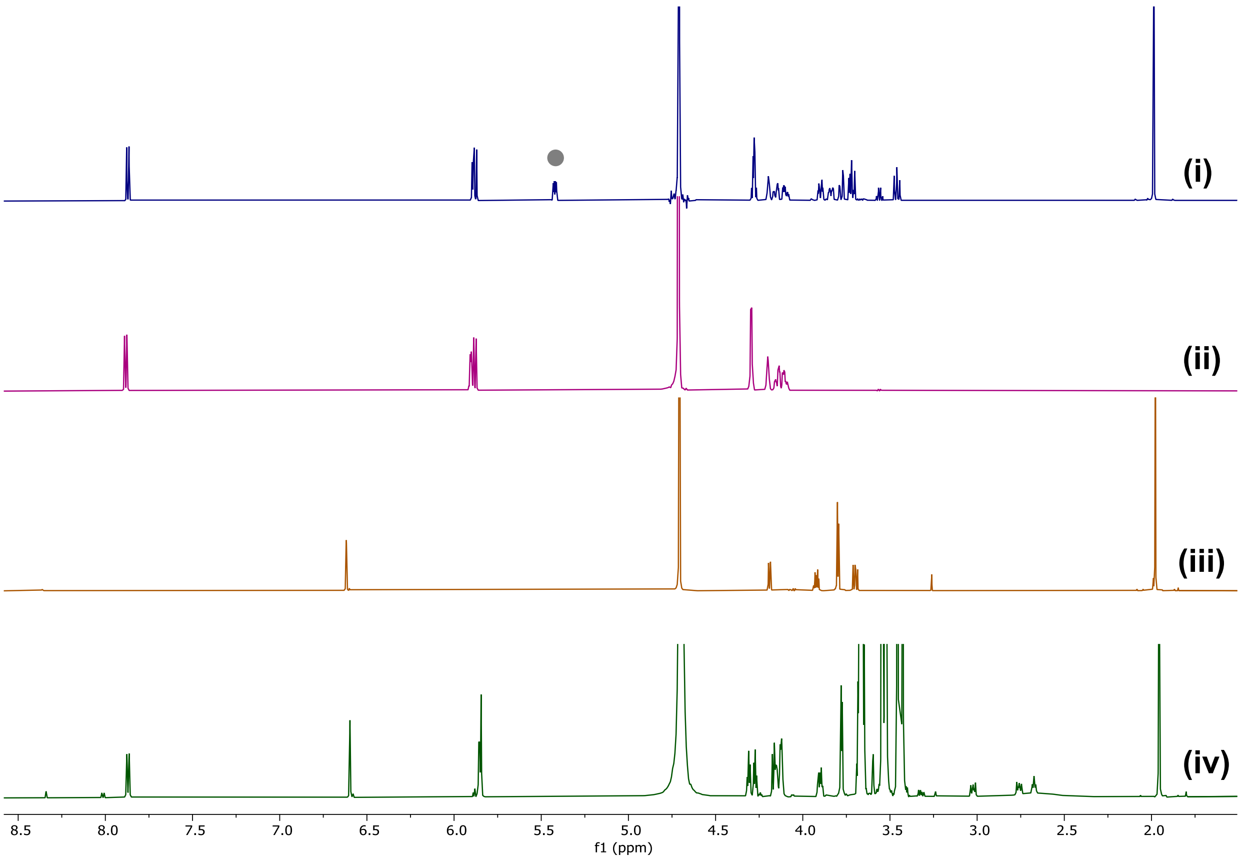

Figure S12. ^{1}H NMR spectra of (i) UDP-GlcNAc **1** standard, (ii) UDP **3** standard, (iii) AAG **2** standard and (iv) the reaction mixture of KasQ incubated with AAG **2**, UDP **3** and $MgCl_2$ at 37°C for 12 hrs. Anomeric proton of UDP-GlcNAc **1** was shown in gray dots.

```
                                                                            1
KasH  .................................................................MPRWAG
KasF  MTAAAASPEDLAPIQARTLFVMRDGRLTAINDVGRPAPPRCFLSYTATAVTAWTSTAVPADLTDELHRWAA

         10                                     20        30        40
KasH  TYDGGV..........................TITYEWRGGFDNAALNALHADGFGPPIAQTDWRTR....
KasF  DQAPAPLTEVVSPPAGALRLLARHAPVTESFVGPAYAVPDSAGTPAPPGLTLRECGPSE.SSRFRETFPDV

                    50         60        70        80        90       100
KasH  .............LERH.SLGWVCAWEDGLIGFVNVVWDGGAHAFILDTVVARHCRSRGVGAALVAKAADE
KasF  ADTLAERQPTVAAFDGHRAVAVCCAARDG............GEAYEAGTETLPSHRGRGLASALVTRWARL

               110            120       130       140
KasH  ARAA......NCEWLHVDFEE.....HLRAFYFDACGFKETTAGLIAL
KasF  VRARGARPLYSTEWDNHSSLSVAKRLAMELYAVNVSLY..........
```

Figure S13. The sequence alignment of KasH and KasF. The identical residues are framed in red boxes, and the similar residues were shown in red letters.

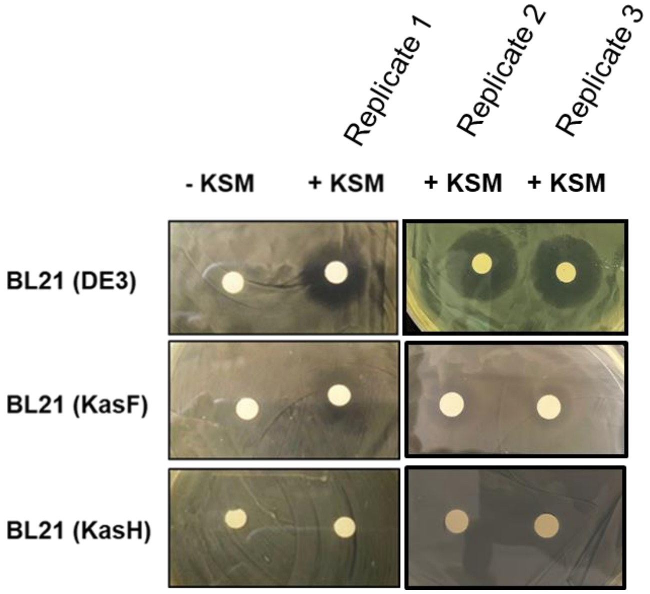

Figure S14. Disc diffusion assay of KasF and KasH with or without KSM. The BL21 (DE3) was the control.

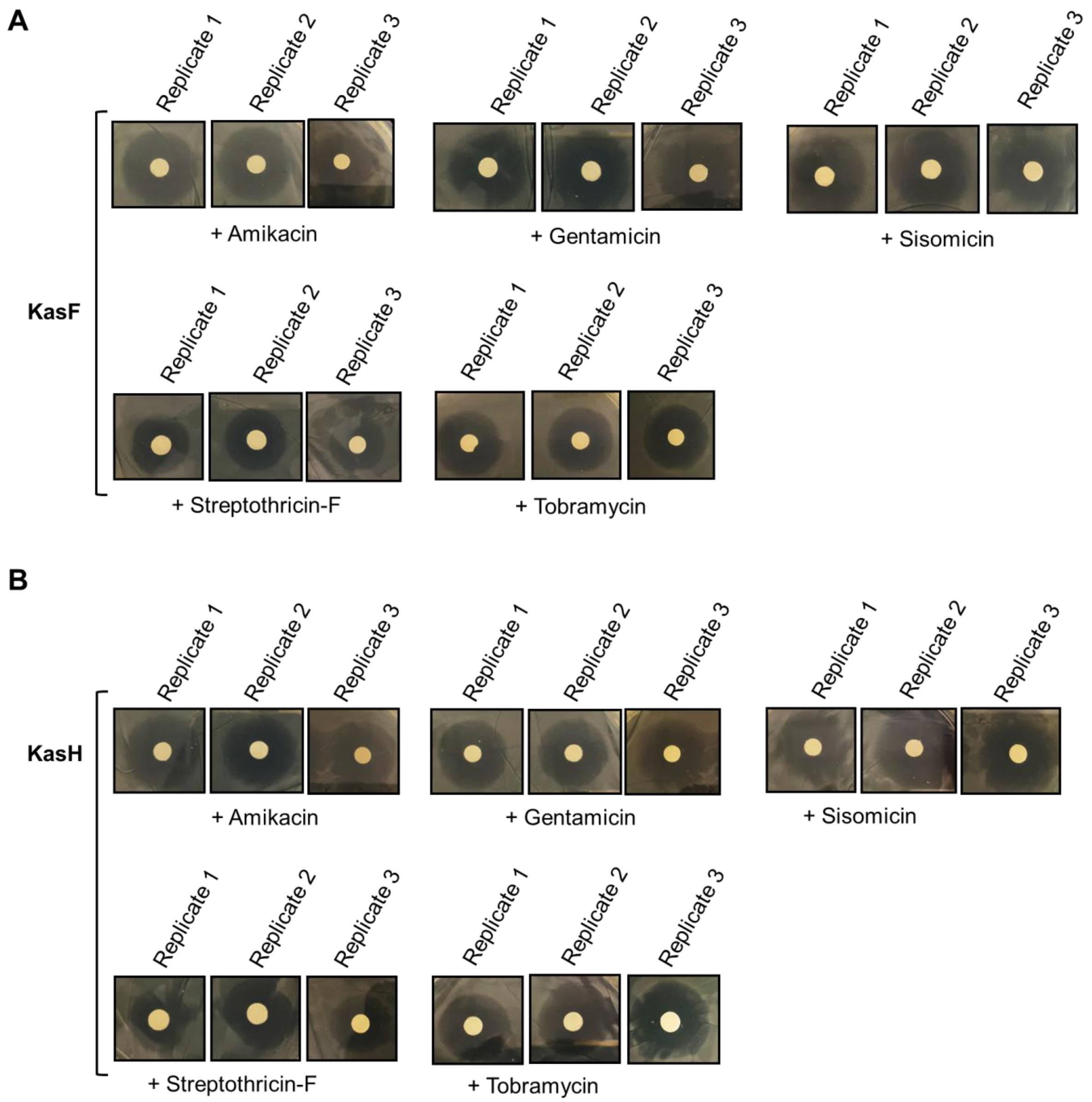

Figure S15. Disc diffusion assay of KasF and KasH with some commonly used aminoglycoside antibiotics.

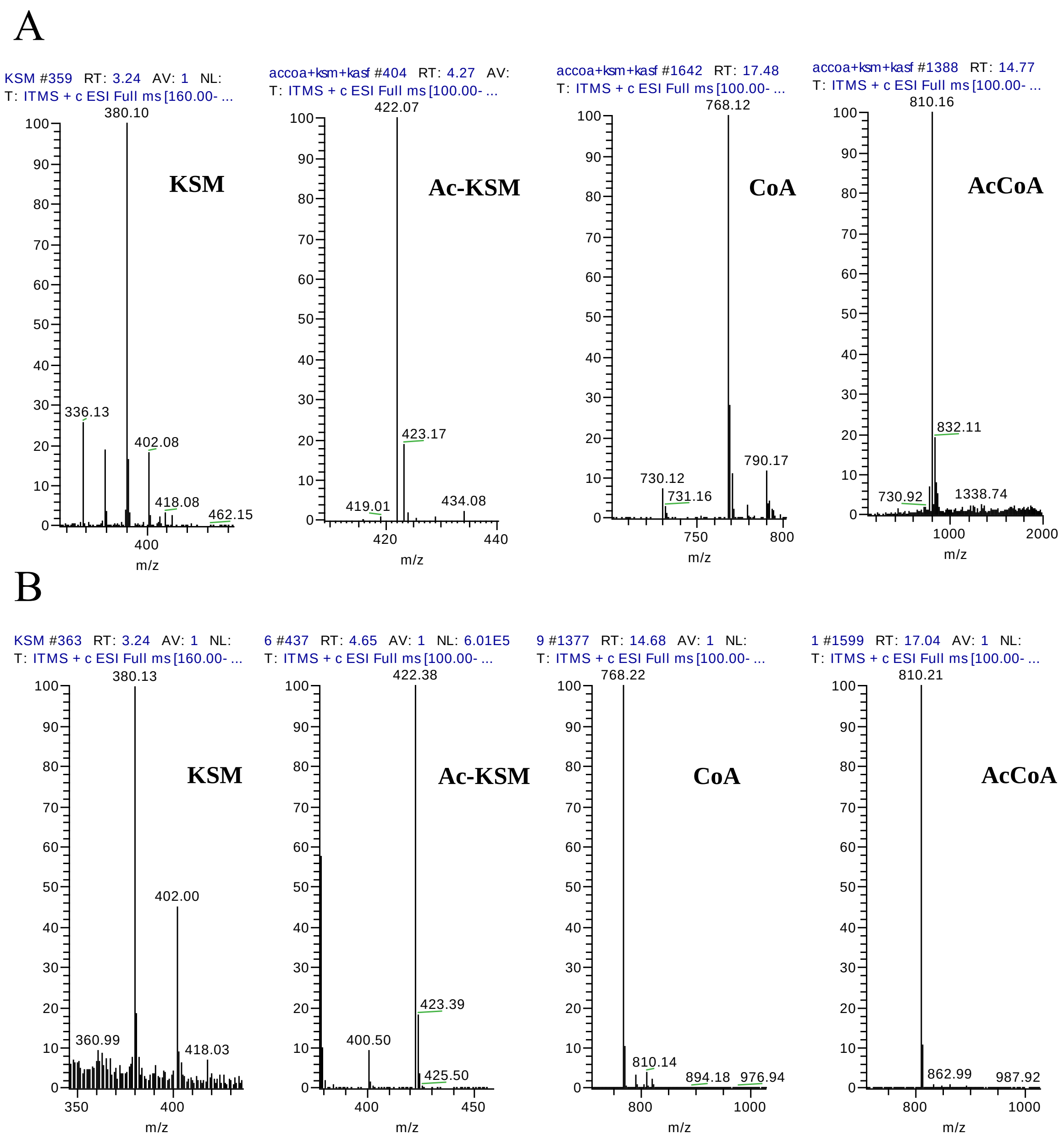

Figure S16. The mass spectra of the substrates and products (in m/z) of the KasF (A) or KasH (B) enzymatic reactions.

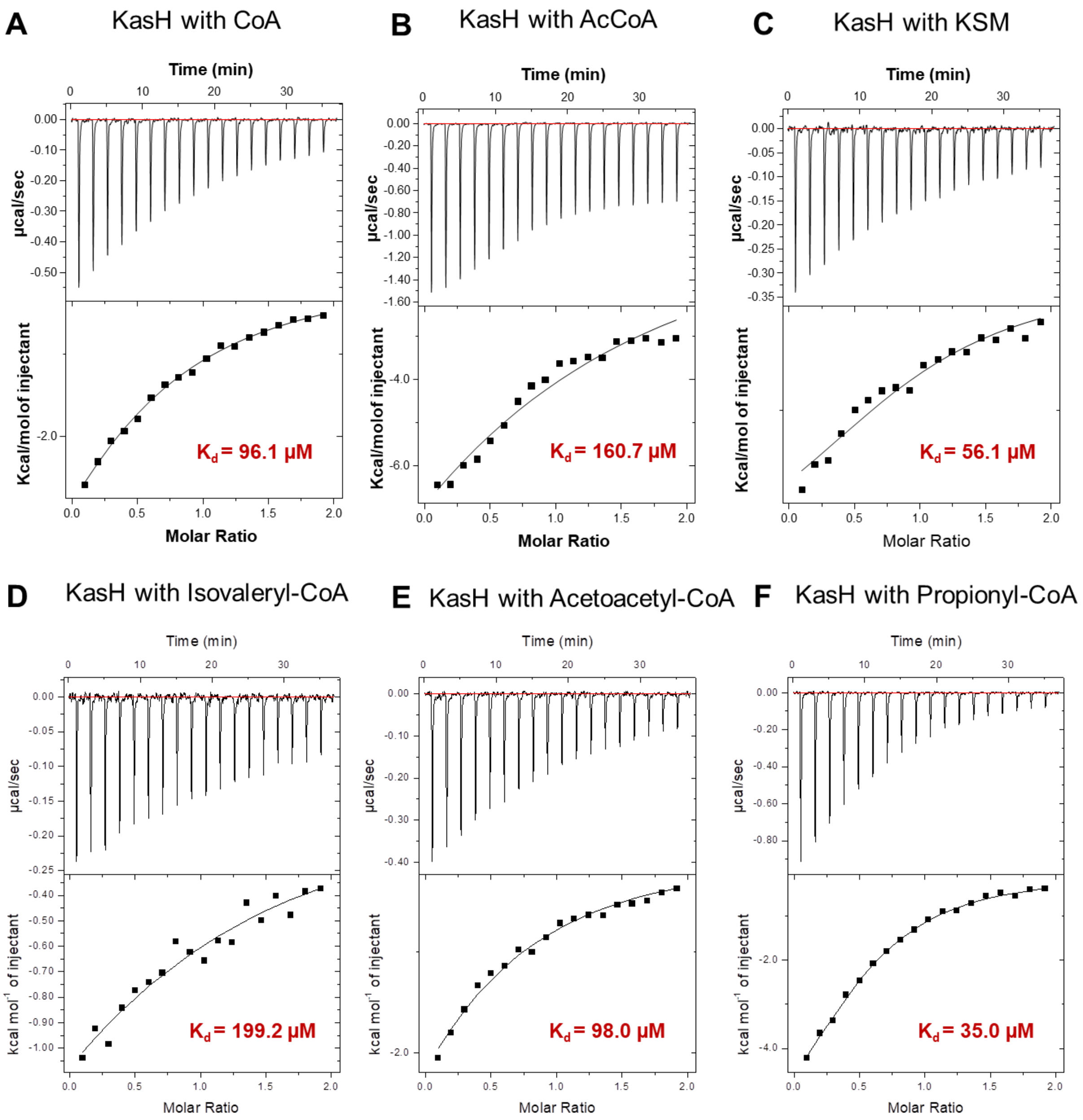

Figure S17. Isothermal Titration Calorimetry (ITC) analysis. (A-F) Titration of KasH with CoA, AcCoA, isovaleryl-CoA, acetoacetyl-CoA, propionyl-CoA and KSM. The baseline corrected titration data (top panel) and the integrated data (squares-bottom panel) are fitted with the "one binding site" model in ORIGIN. Fitting parameters are summarized in **Table S1**.

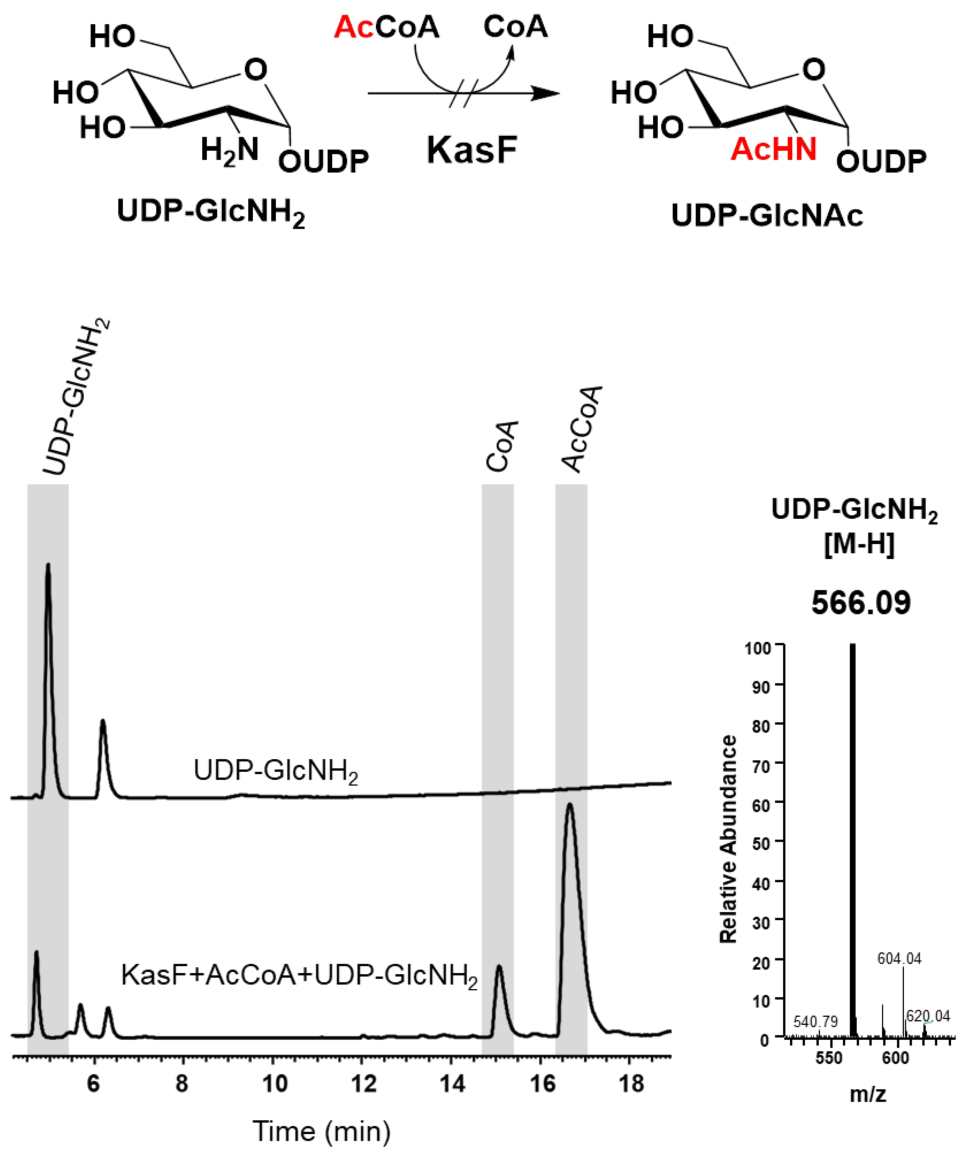

Figure S18. Acetylation assay of KasF with UDP-GlcNH$_2$ and AcCoA. The second peak is the degraded moiety from UDP-GlcNH$_2$ when we enzymatically synthesized UDP-GlcNH$_2$ (which is not commercially available). However, it does not impact on the KasF activity in the presence of UDP-GlcNH$_2$ and AcCoA. In this enzymatic reaction, we can clearly confirm that the substrate UDP-GlcNH$_2$ m/z 566.09 [M-H] is not subject to acetylation of KasF.

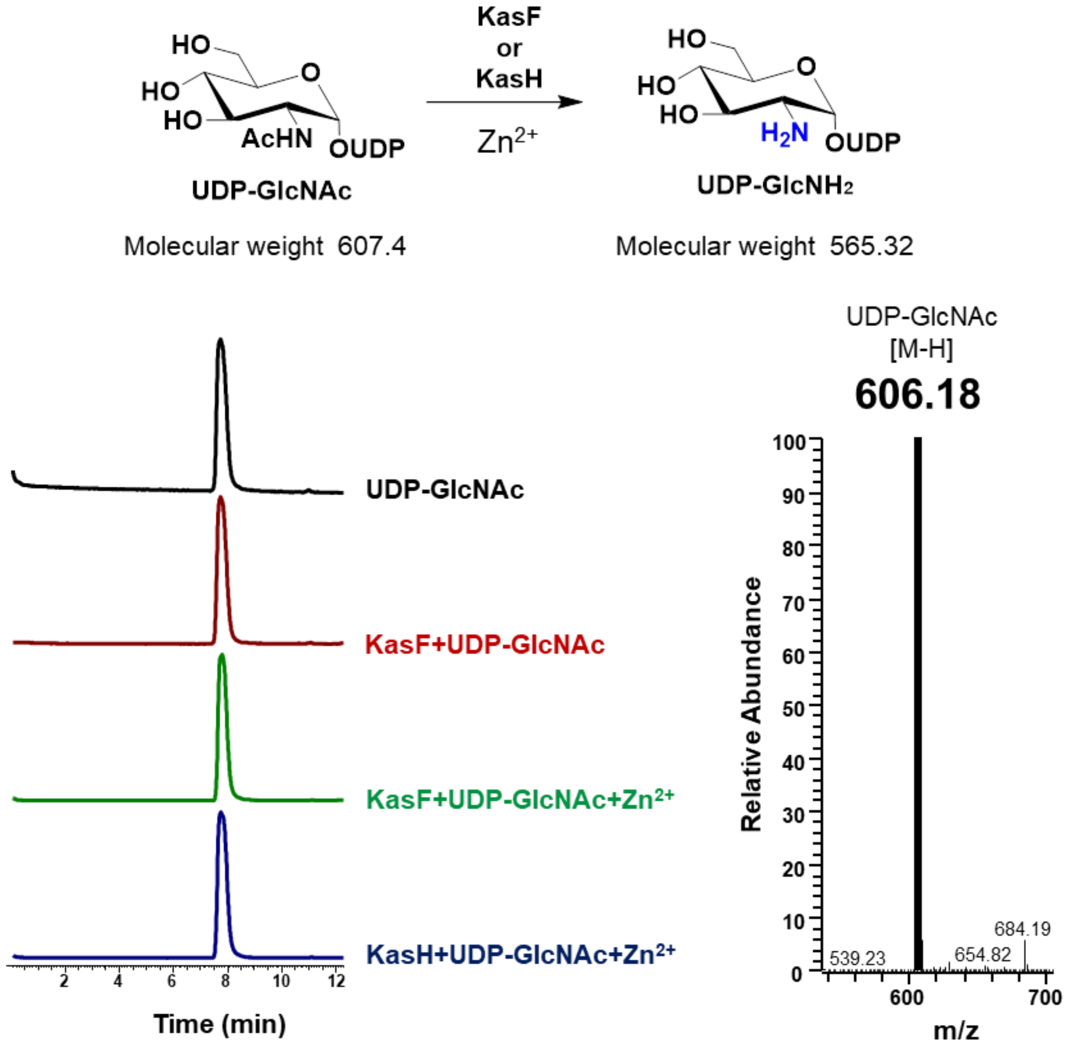

Figure S19. Deacetylation assay for KasH or KasF with UDP-GlcNAc and Zn^{2+}, confirming that both enzymes are lack of the deacetylation activity.

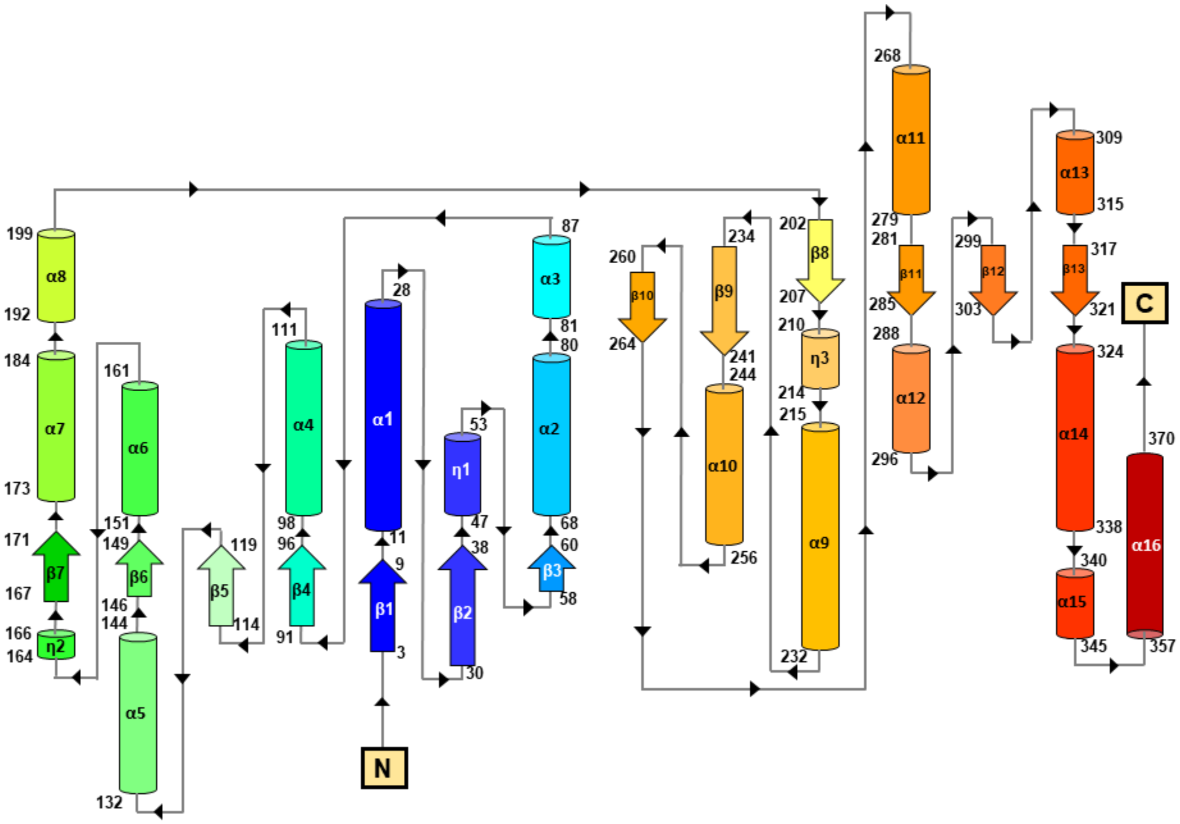

Figure S20. Topology diagram of the protein fold of the KasQ protomer (the N-terminal site is represented in blue and the C-terminal site is represented in red).

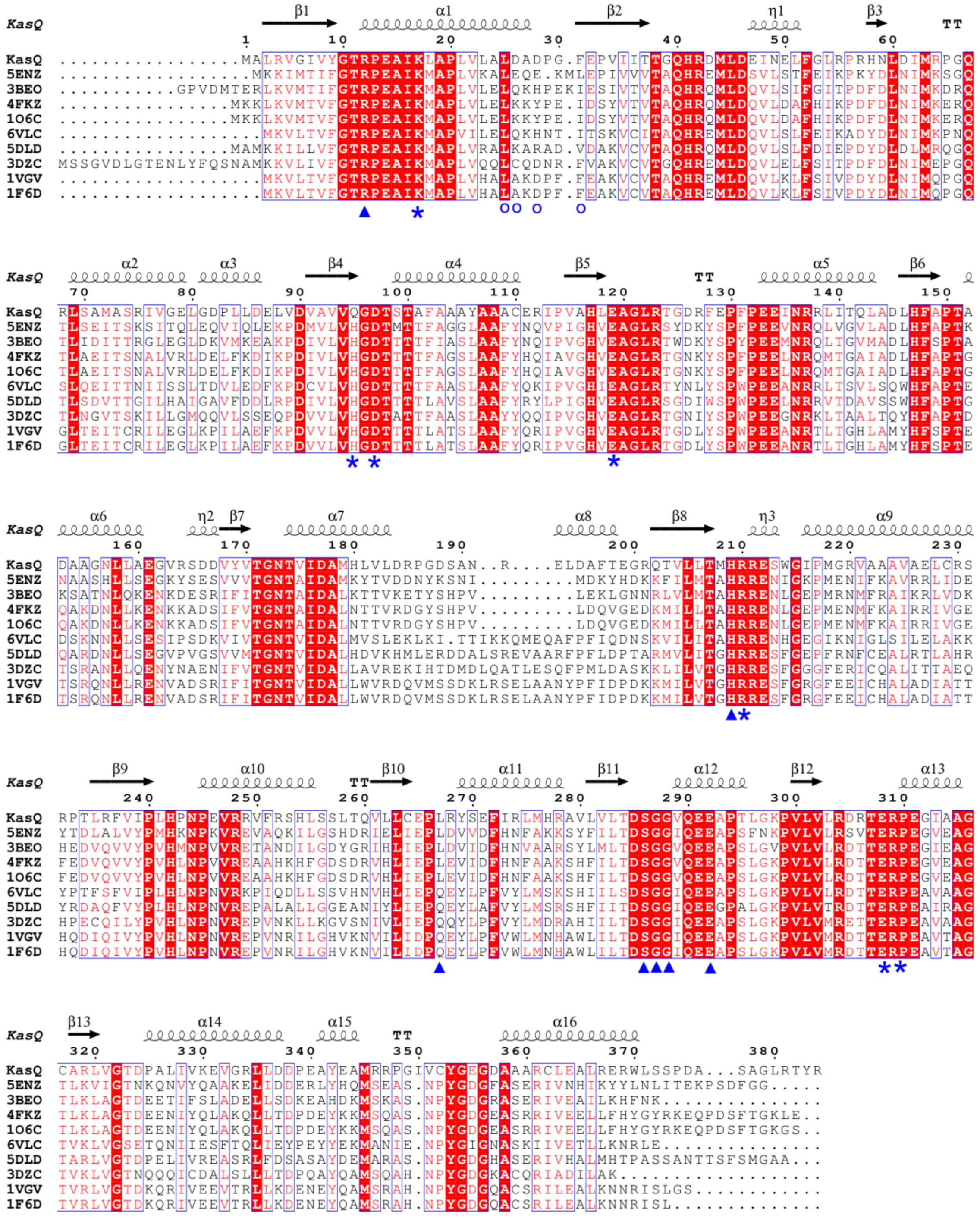

Figure 21. Sequence alignment of KasQ with different UDP-GlcNAc 2-epimerases. The identical residues are represented in red boxes, and the similar residues are represented in red letters. KasQ-UDP binding residues are labeled in triangle, the sugar binding residues are labeled in asterisks and the sodium-ion binding residues are labeled in round.

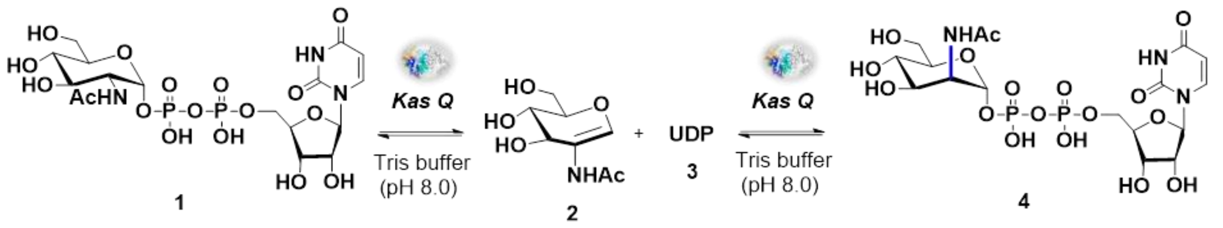

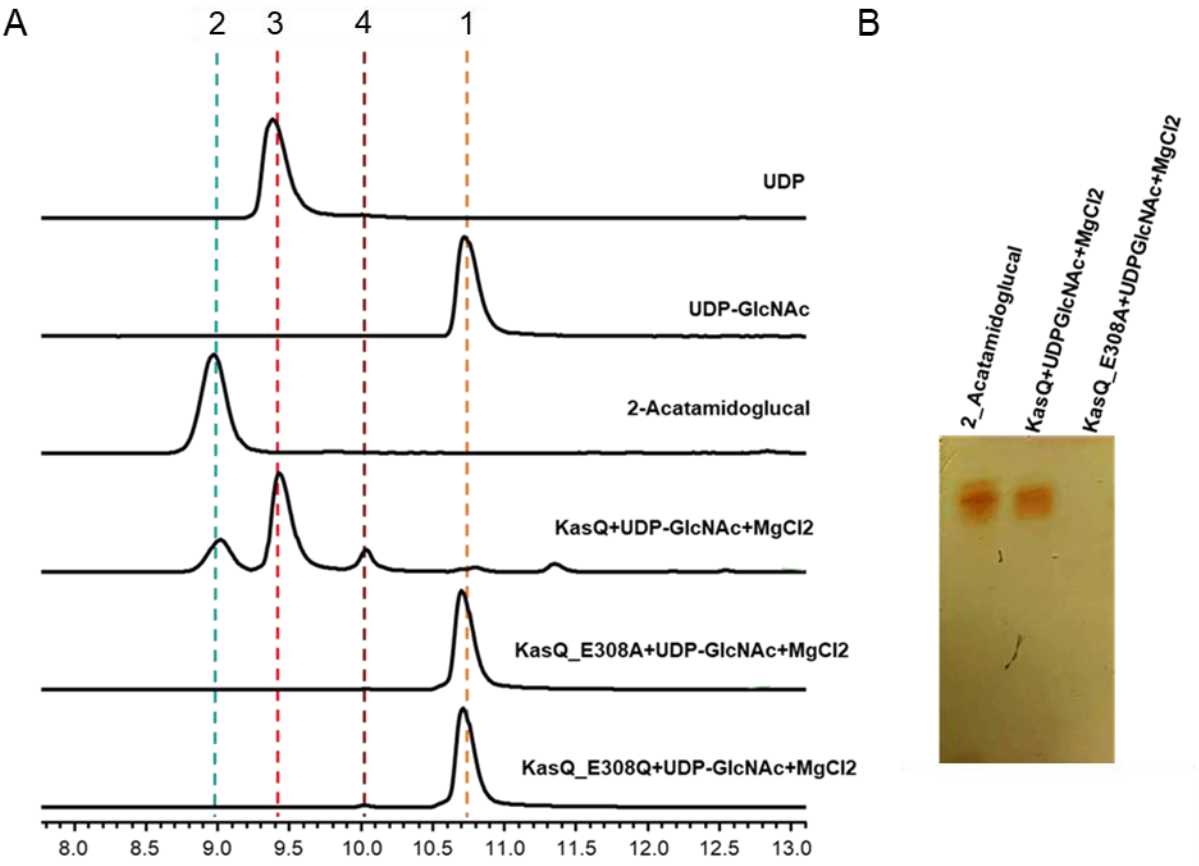

Figure S22. Biochemical assay of KasQ mutants E308A and E308Q. (A) HPLC traces of KasQ mutants E308A and E308Q in reaction with UDP-GlcNAc and $MgCl_2$. Wild type KasQ was used as a positive control. KasQ can generate UDP-ManNAc, UDP and 2-actamidoglucal, but KasQ mutants E308A and E308Q are inactive. (B) TLC analysis for the reaction mixtures of KasQ and its mutants.

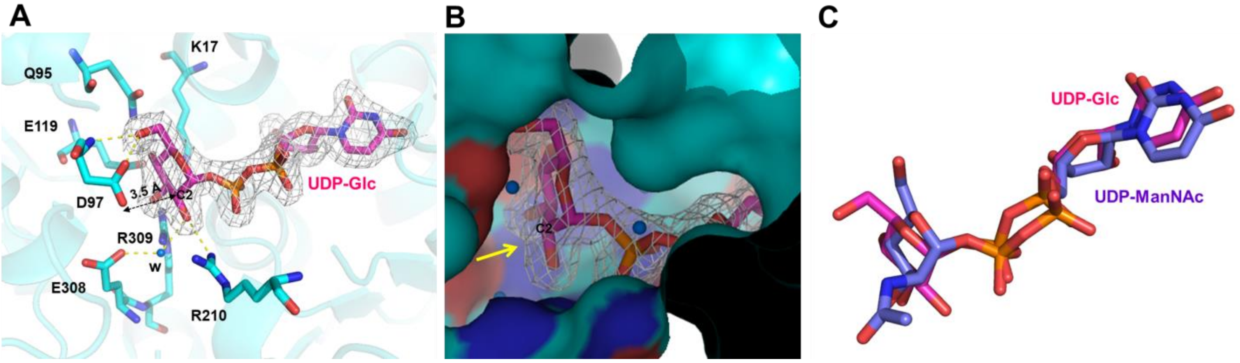

Figure S23. The active site and the binding pocket of KasQ. (A) UDP-Glc interacts with surrounding amino acid residues at the active site. The distance between Glu97 to glucose-C2 position is 3.5 Å. (B) The binding pocket of KasQ can accept the sugar moiety. Yellow arrow indicates the extra pocket for accommodation of the acetyl group of UDP-GlcNAc or UDP-ManNAc. (C) Superimposition of the ligand UDP-Glc (from the KasQ_UDP-Glc complex structure) and UDP-ManNAc (from PDB entry 1vgv).

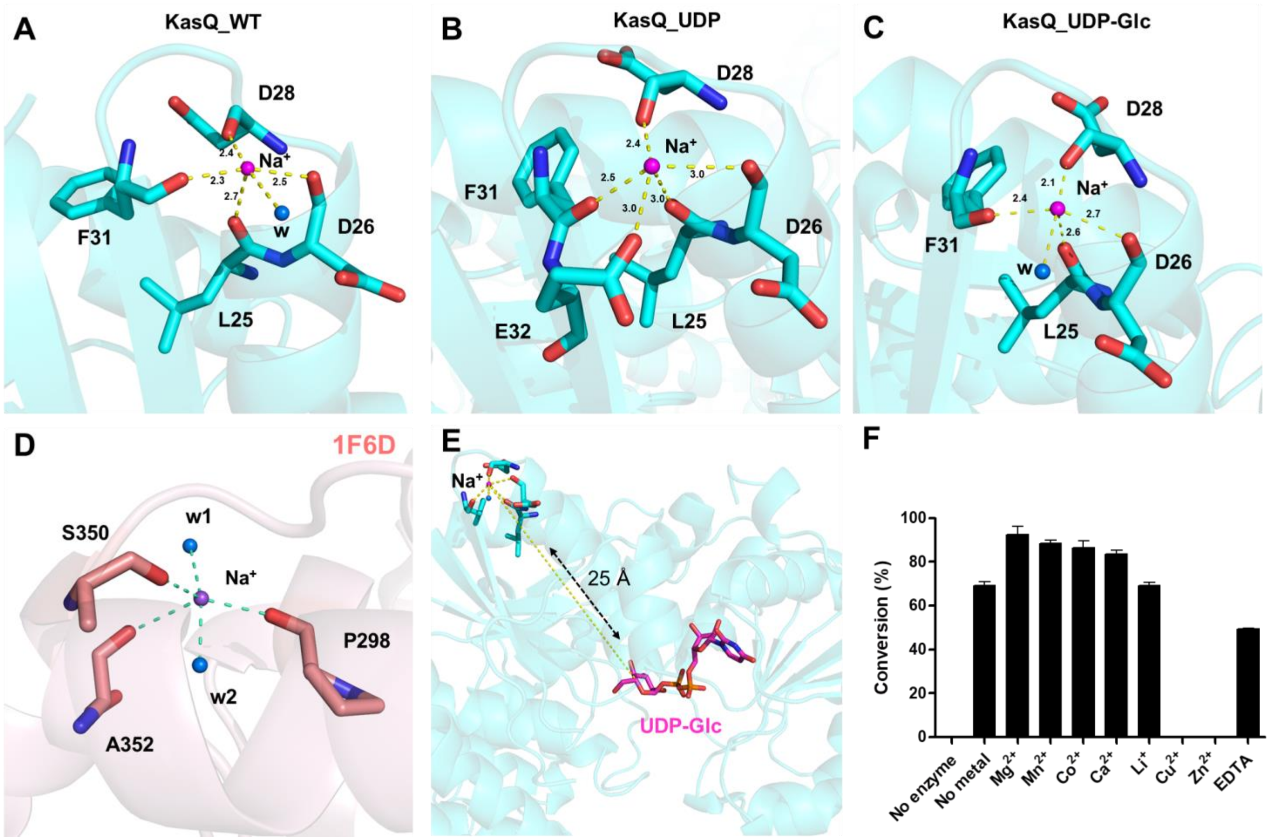

Figure S24. The metal-ion binding site of KasQ. (A-C) KasQ sodium binding residues in ligand-free and ligand-bound complex structures. (D) The sodium binding site of KasQ homolog (PDB entry 1F6D). (E) The distance between the substrate binding pocket (the UDP-Glc binding site) and the sodium-ion binding site. (F) Effects of metal ions on enzyme activity.

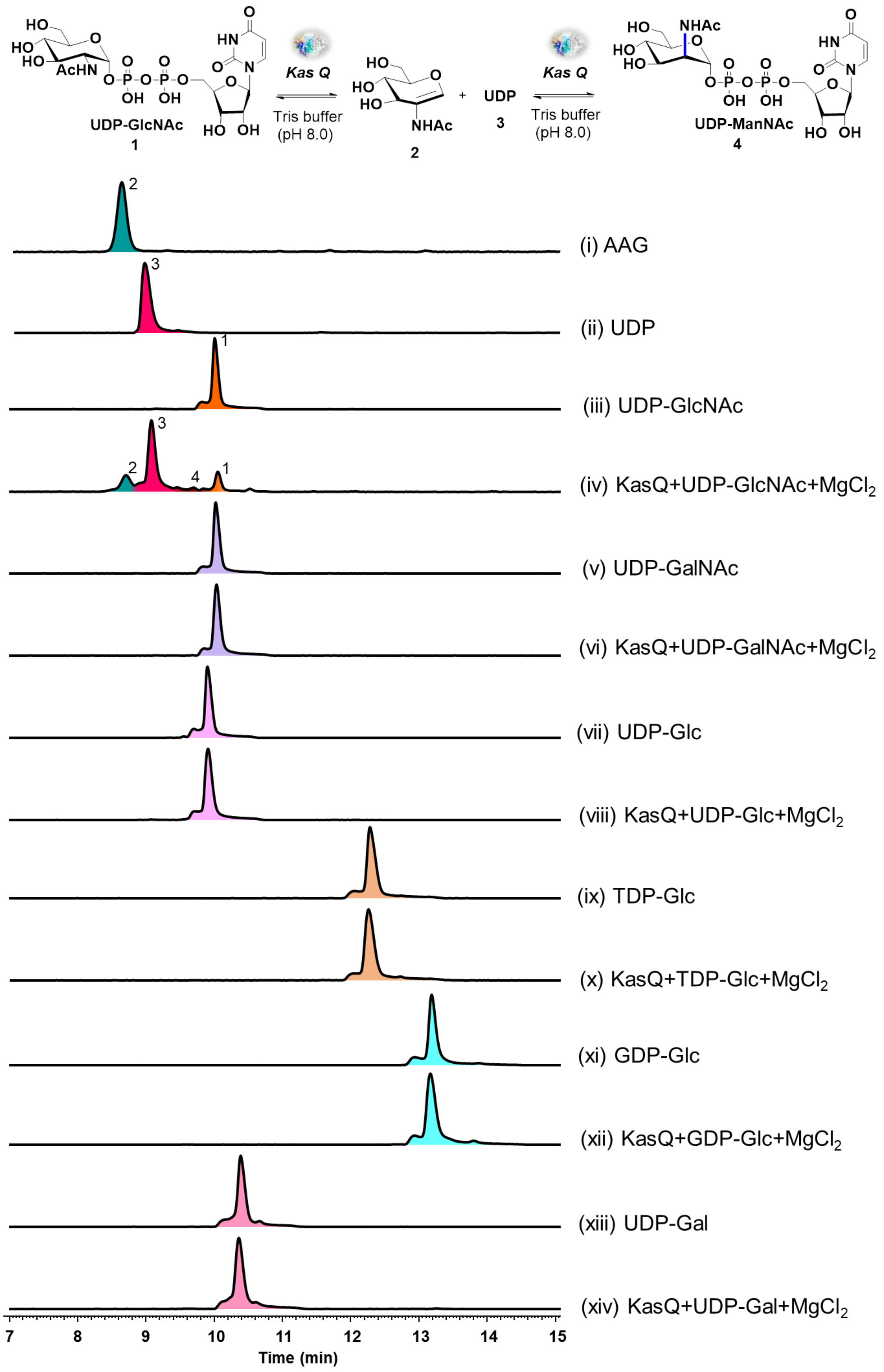

Figure S25. Enzymatic reactions of KasQ with different NDP sugars.

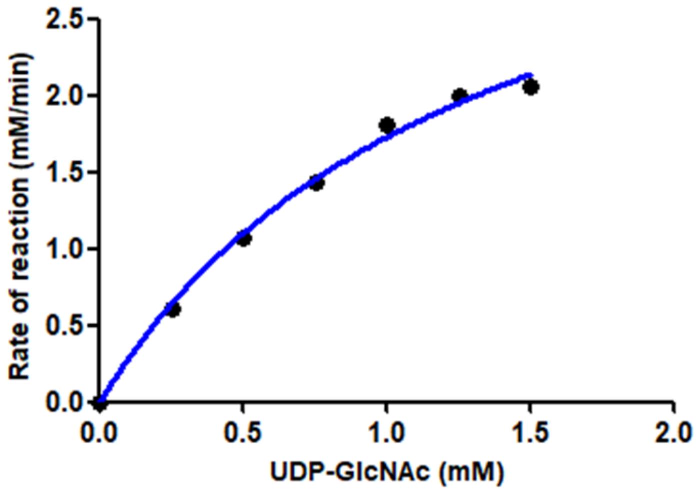

Figure S26. The kinetic curve of KasQ for the epimerization reactions.

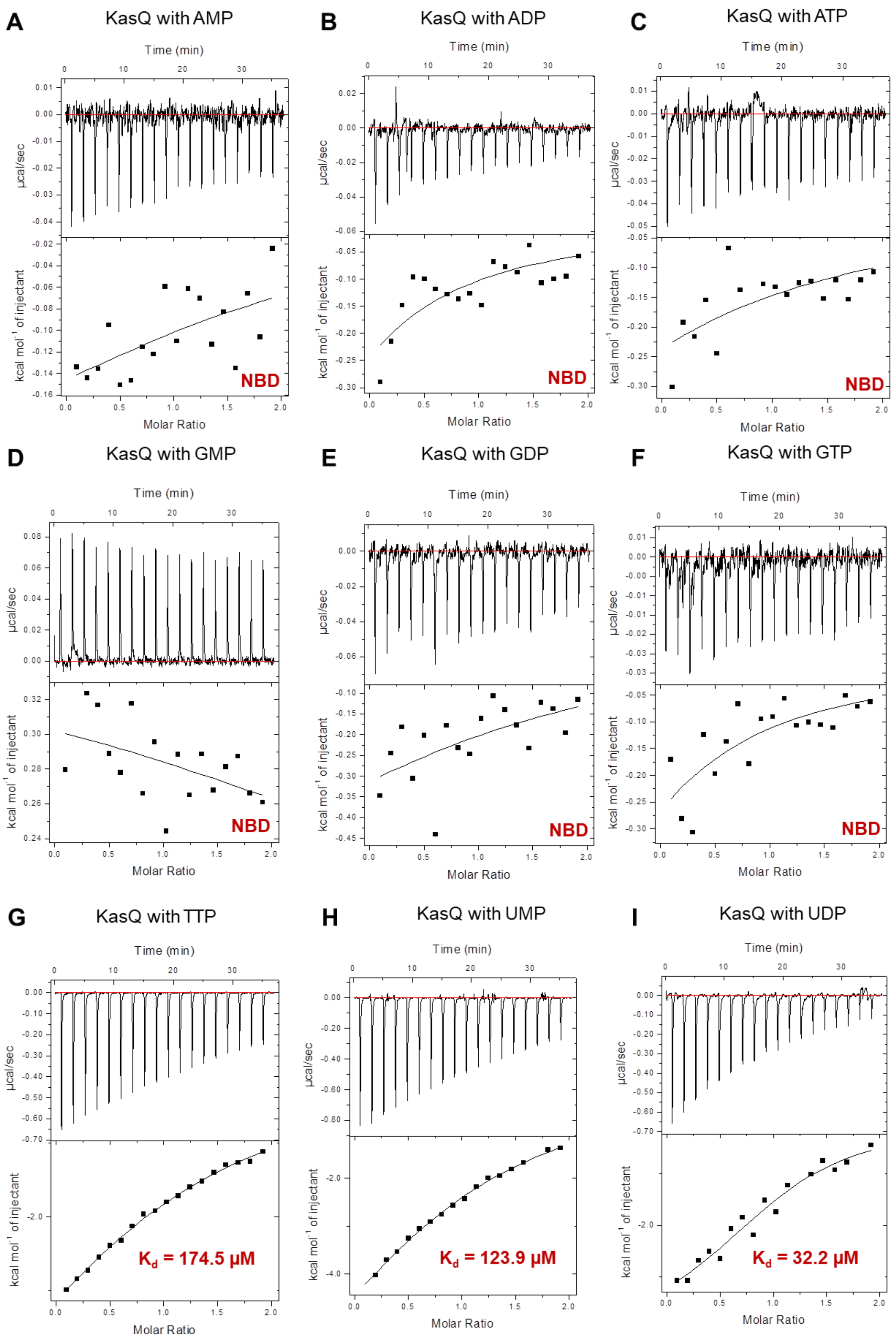

A
KasQ with AMP
NBD
B
KasQ with ADP
NBD
C
KasQ with ATP
NBD
D
KasQ with GMP
NBD
E
KasQ with GDP
NBD
F
KasQ with GTP
NBD
G
KasQ with TTP
K_d = 174.5 μM
H
KasQ with UMP
K_d = 123.9 μM
I
KasQ with UDP
K_d = 32.2 μM
Time (min)
μcal/sec
kcal mol⁻¹ of injectant
Molar Ratio

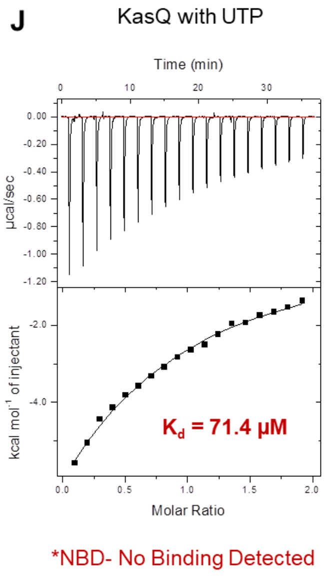

Figure S27. ITC analyses of KasQ with different nucleosides (mono/di/tri) phosphates. (A-J) Titration of KasQ with different nucleosides (mono/di/tri) phosphates. The baseline corrected titration data (top panel) and the integrated data (squares-bottom panel), together with the best fits (solid lines) according to the single-site binding model. Fitting parameters are summarized in **Table 4**.

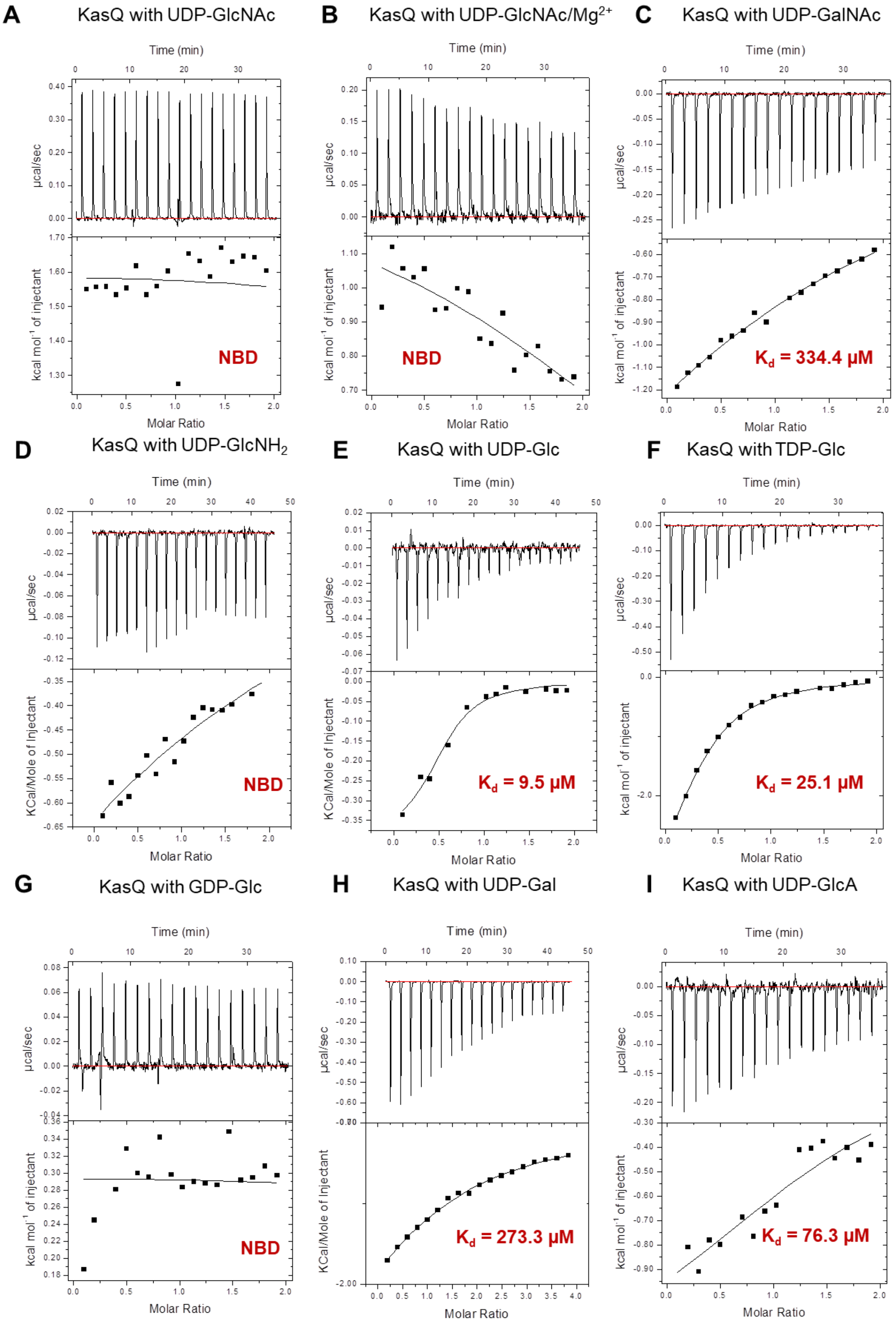
A
KasQ with UDP-GlcNAc
NBD
B
KasQ with UDP-GlcNAc/Mg2+
NBD
C
KasQ with UDP-GalNAc
Kd = 334.4 μM
D
KasQ with UDP-GlcNH2
NBD
E
KasQ with UDP-Glc
Kd = 9.5 μM
F
KasQ with TDP-Glc
Kd = 25.1 μM
G
KasQ with GDP-Glc
NBD
H
KasQ with UDP-Gal
Kd = 273.3 μM
I
KasQ with UDP-GlcA
Kd = 76.3 μM
Time (min)
μcal/sec
kcal mol-1 of injectant
KCal/Mole of Injectant
Molar Ratio

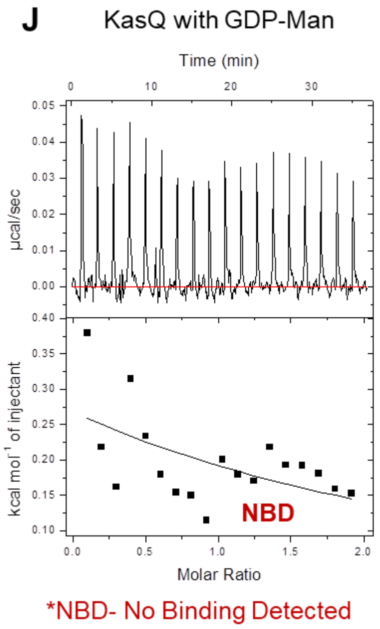

Figure S28. ITC analyses of KasQ with different NDP sugars. (A-J) Titration of KasQ with different NDP sugars. The baseline corrected titration data (top panel) and the integrated data (squares-bottom panel), together with the best fits (solid lines) according to the single-site binding model. Fitting parameters are summarized in **Table 4**.

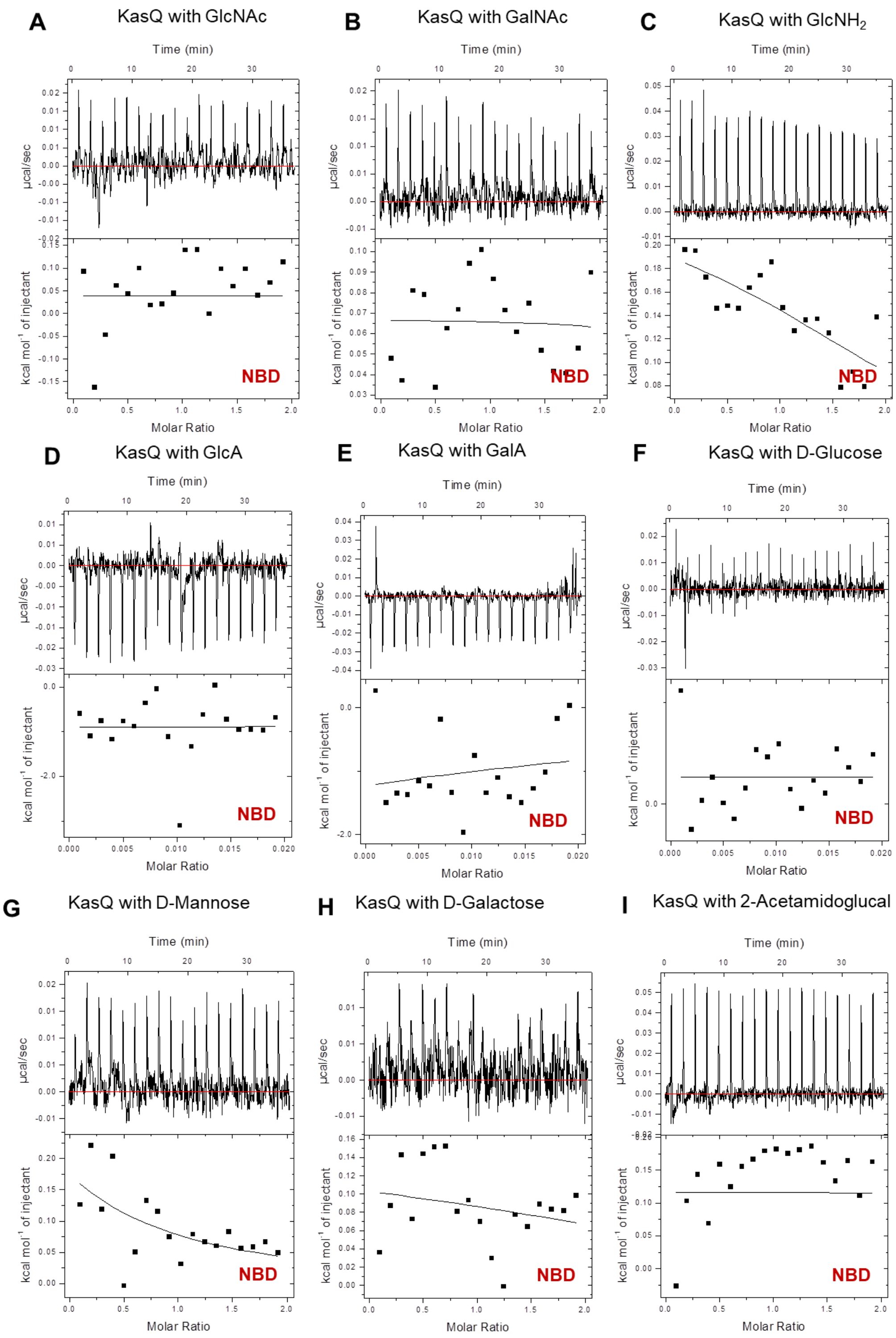

Figure S29. ITC analyses of KasQ with different monosaccharides. (A-J) Titration of KasQ with different monosaccharides. The baseline corrected titration data (top panel) and the integrated data (squares-bottom panel), together with the best fits (solid lines) according to the single-site binding model. Fitting parameters are summarized in **Table 4**.

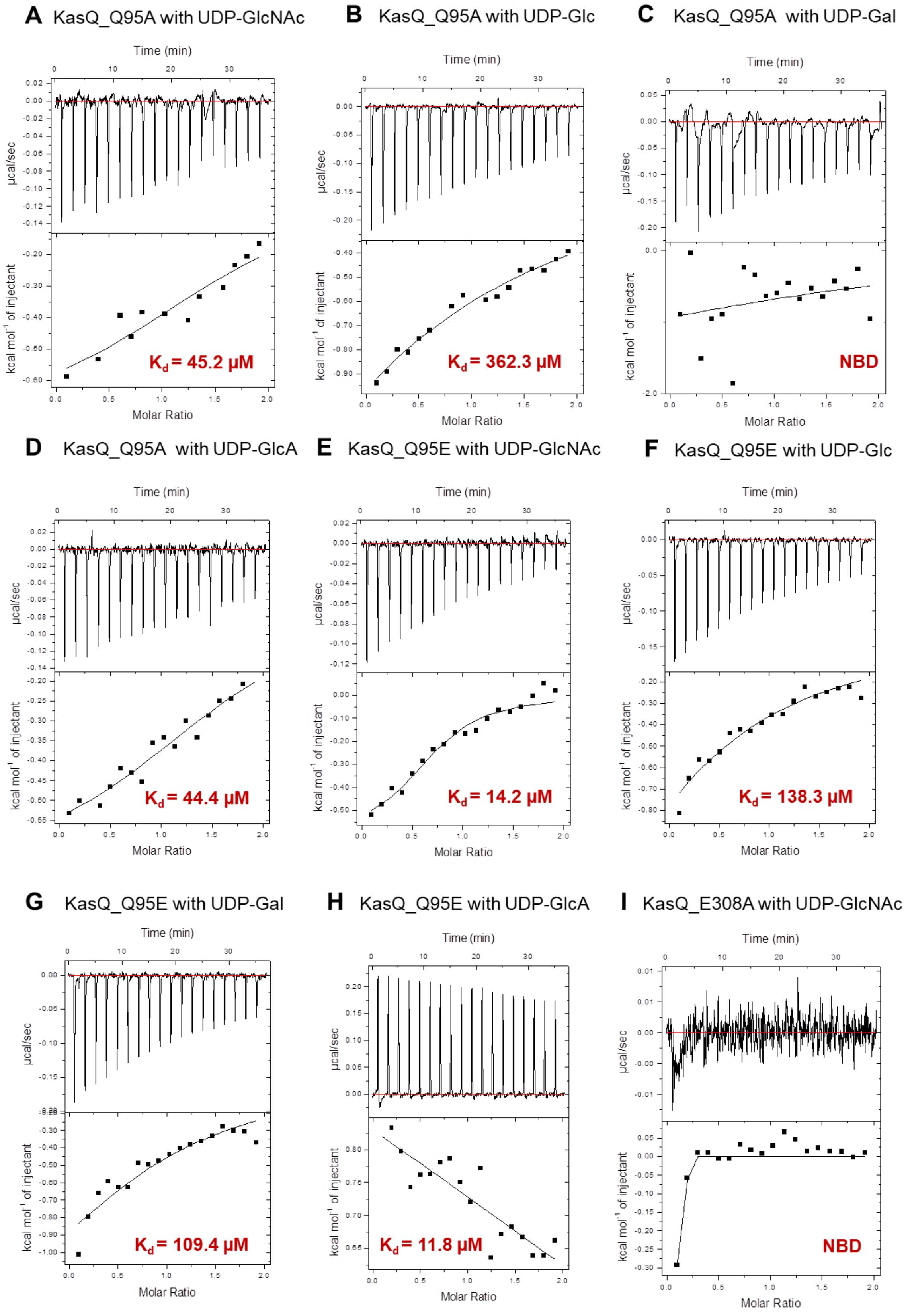
A KasQ_Q95A with UDP-GlcNAc
K_d = 45.2 μM
B KasQ_Q95A with UDP-Glc
K_d = 362.3 μM
C KasQ_Q95A with UDP-Gal
NBD
D KasQ_Q95A with UDP-GlcA
K_d = 44.4 μM
E KasQ_Q95E with UDP-GlcNAc
K_d = 14.2 μM
F KasQ_Q95E with UDP-Glc
K_d = 138.3 μM
G KasQ_Q95E with UDP-Gal
K_d = 109.4 μM
H KasQ_Q95E with UDP-GlcA
K_d = 11.8 μM
I KasQ_E308A with UDP-GlcNAc
NBD
Time (min)
µcal/sec
kcal mol^{-1} of injectant
Molar Ratio

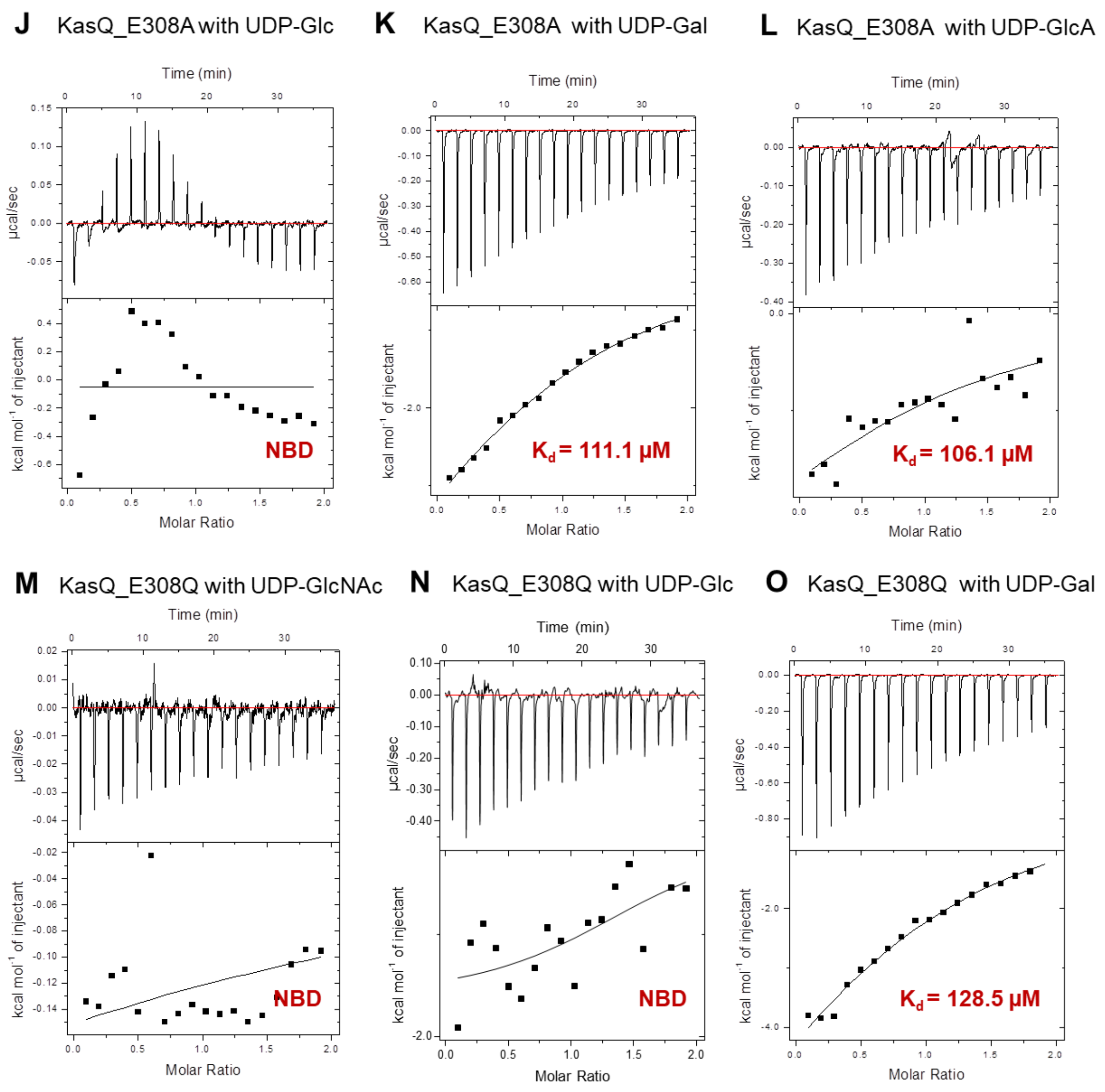

*NBD- No Binding Detected

Figure S30. ITC analyses of KasQ mutants with different NDP sugars. (A-O) Titration of KasQ mutants with different sugars. The baseline corrected titration data (top panel) and the integrated data (squares-bottom panel), together with the best fits (solid lines) according to the single-site binding model. Fitting parameters are summarized in **Table S3**.

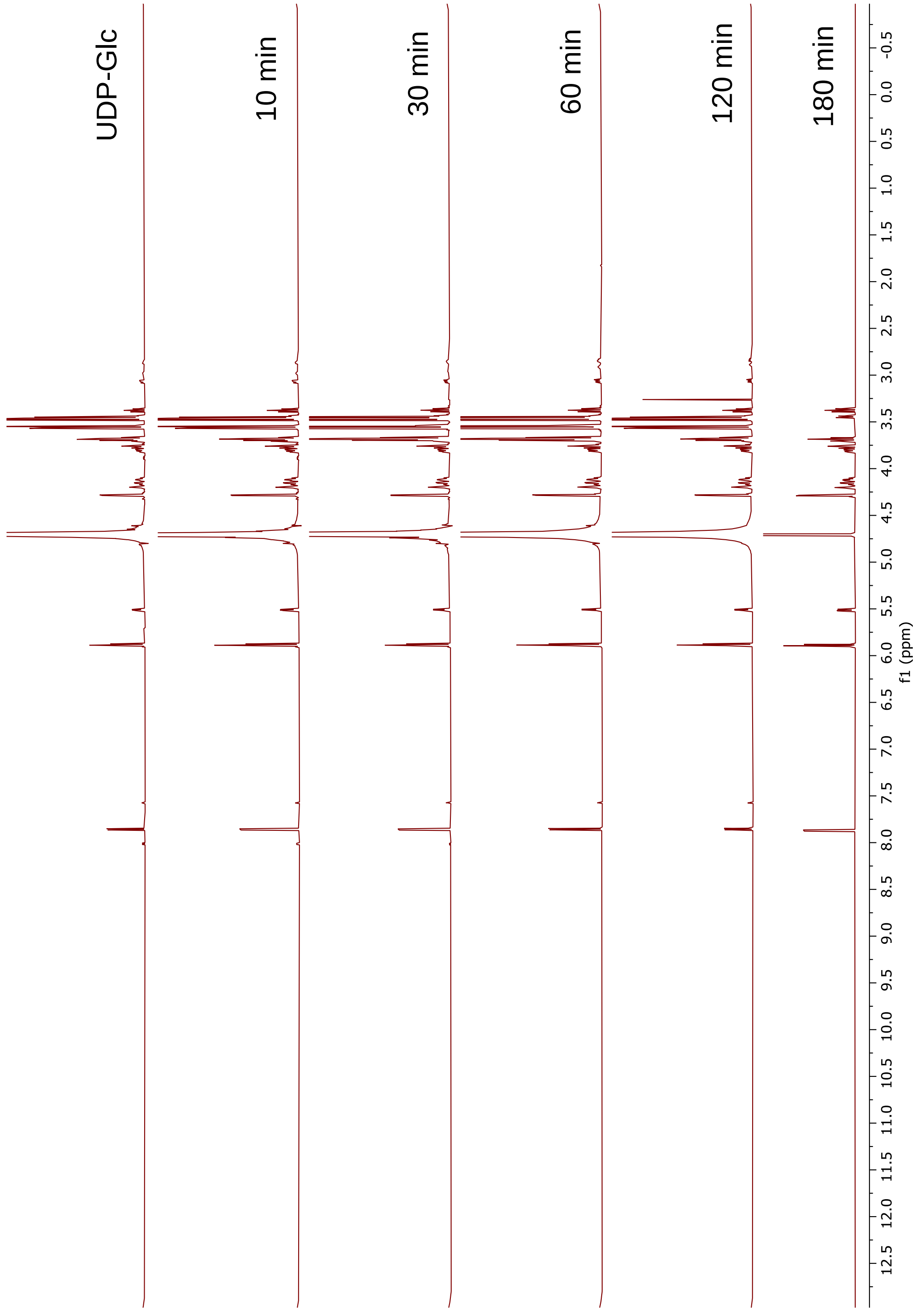

Figure S31. ^{1}H NMR spectra of KasQ in time course reactions.

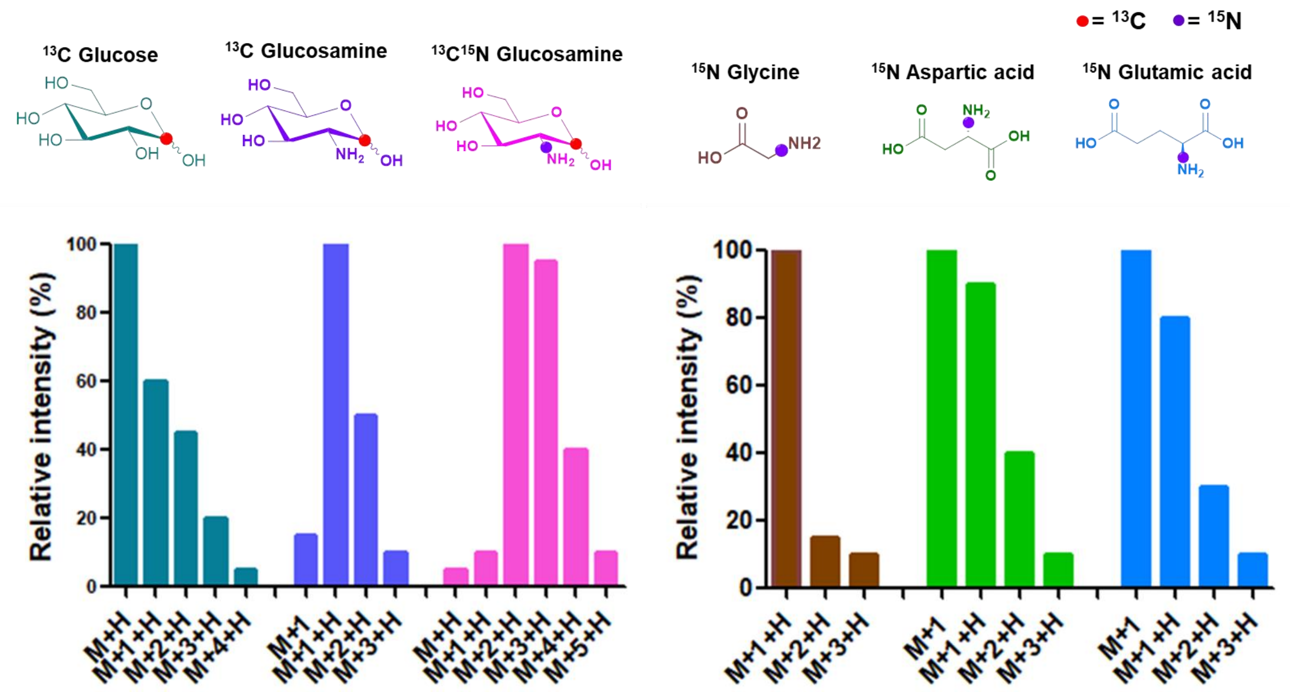

Figure S32. Relative intensity of isotopes in mass spectra from different labeled sugar donors in order to probe the correct kasugamycin biosynthetic pathway.

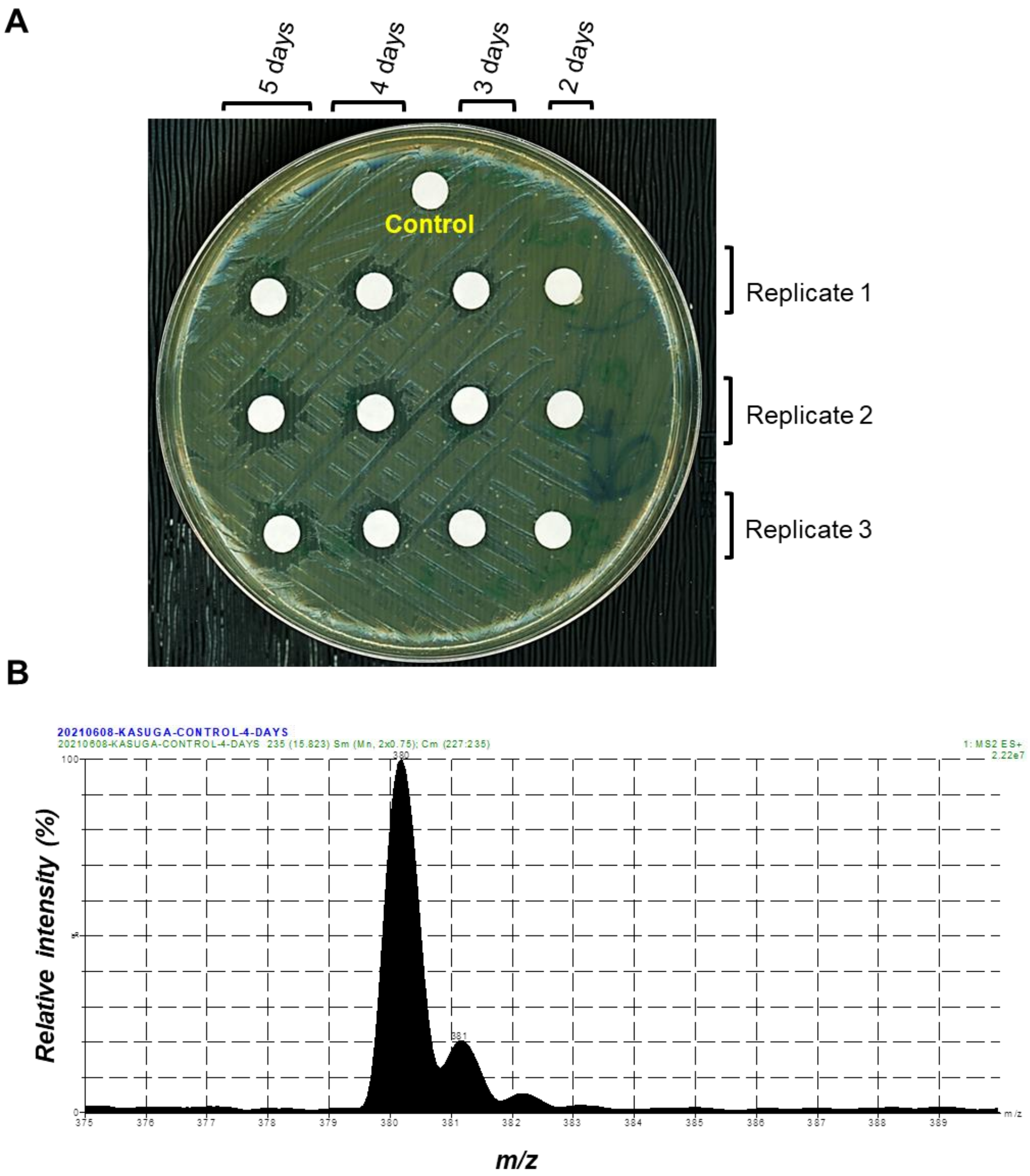

Figure S33. Disc diffusion test and mass spectrum of KSM. (A) Disc diffusion test of KSM produced by *Streptomyces lividans* TK64. The zone of inhibition (ZOI) can be determined in day 3; KSM shows antimicrobial activity against *E. coli*. No ZOI was observed in the control disc suggesting that the growth of *E. coli* was affected only by KSM. (B) Mass spectrum of KSM from the culture medium of the KSM producing strain, *Streptomyces lividans* TK64.

^{1}H NMR (600 MHz, $CDCl_3$)

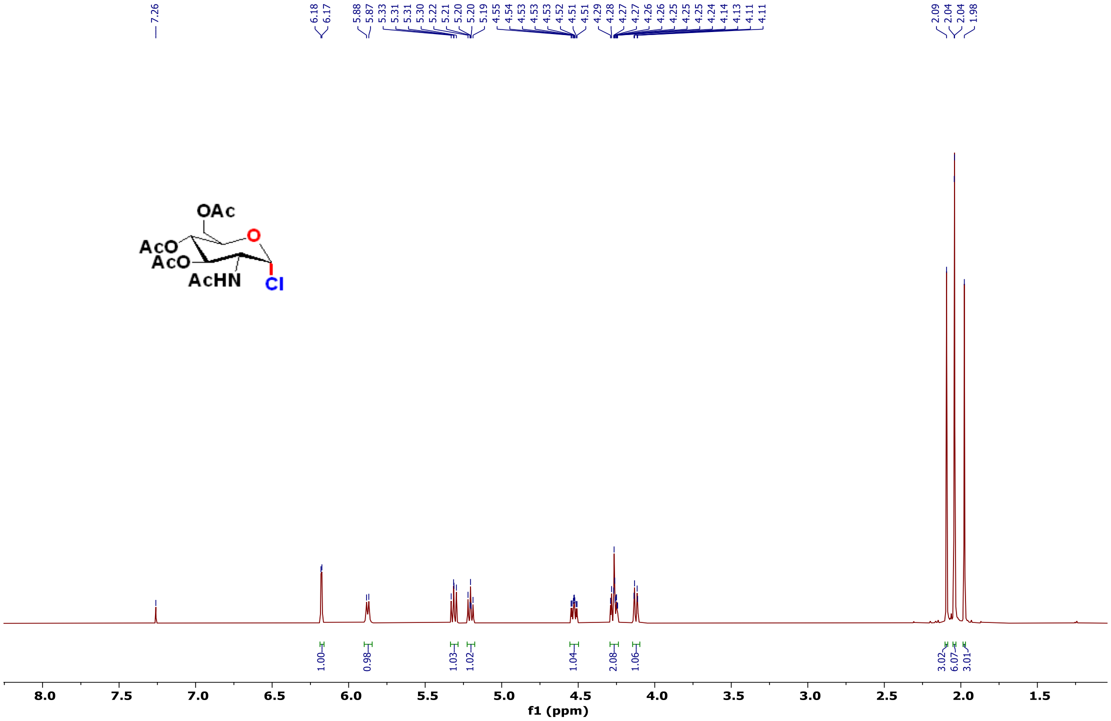

^{13}C NMR (150 MHz, $CDCl_3$)

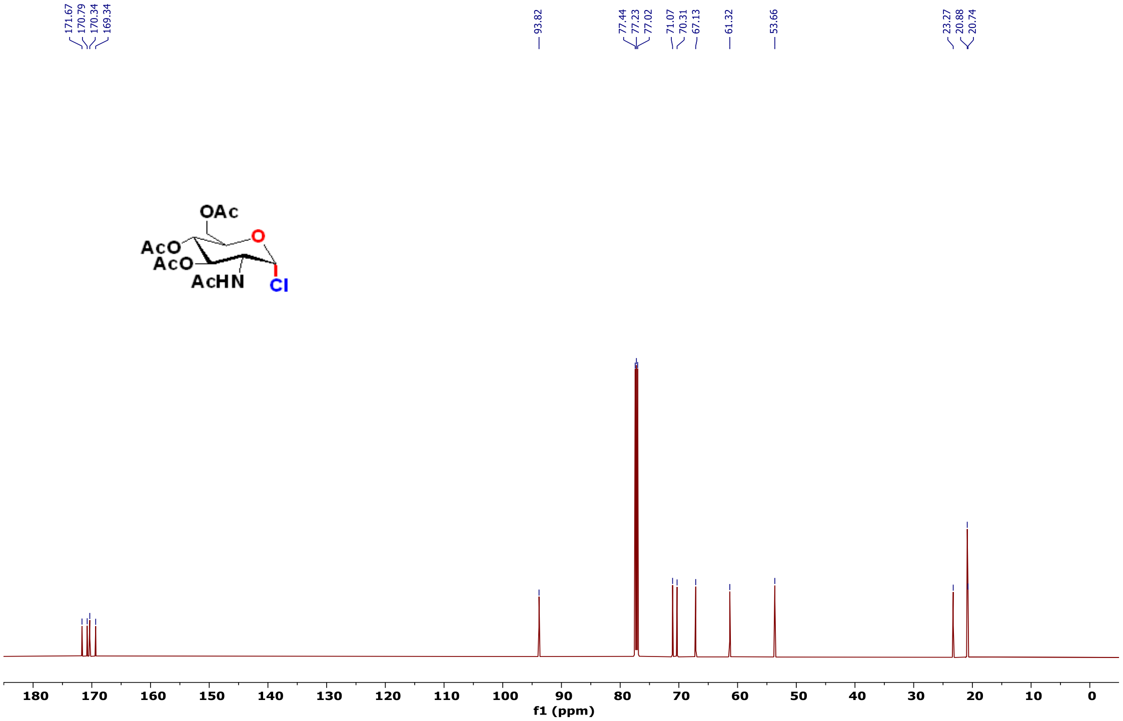

Figure S34. ^{1}H and ^{13}C NMR spectra.

^{1}**H NMR** (600 MHz, $CDCl_3$)

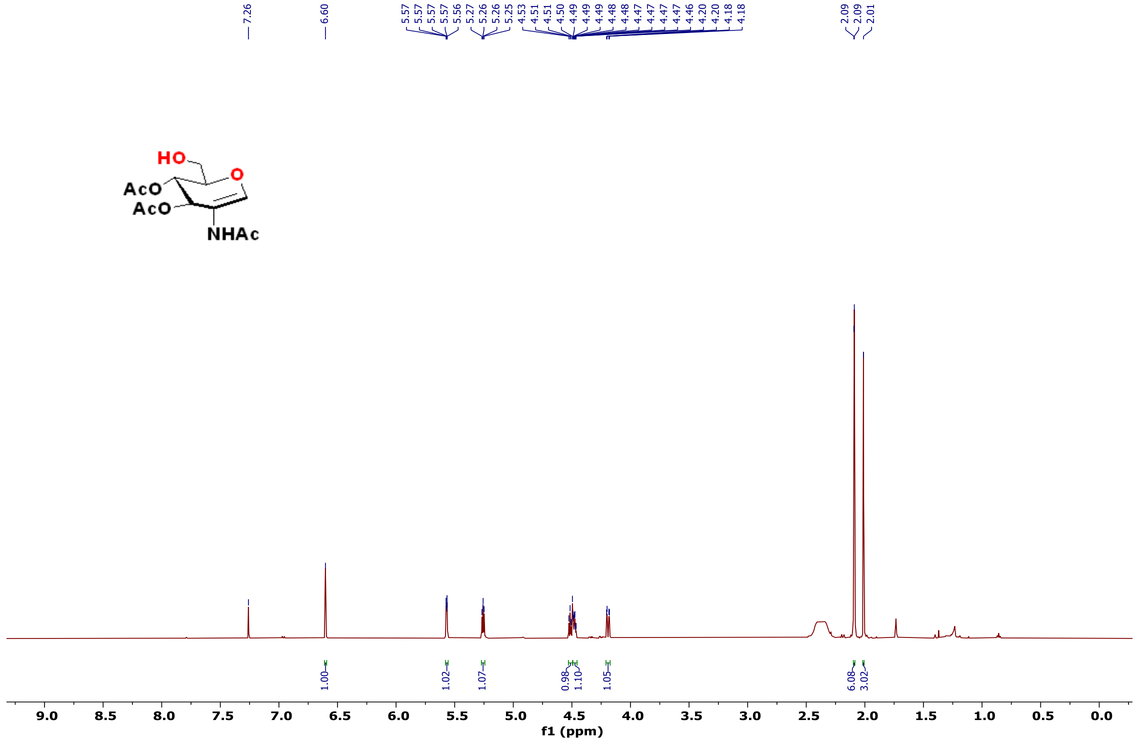

^{13}**C NMR** (150 MHz, $CDCl_3$)

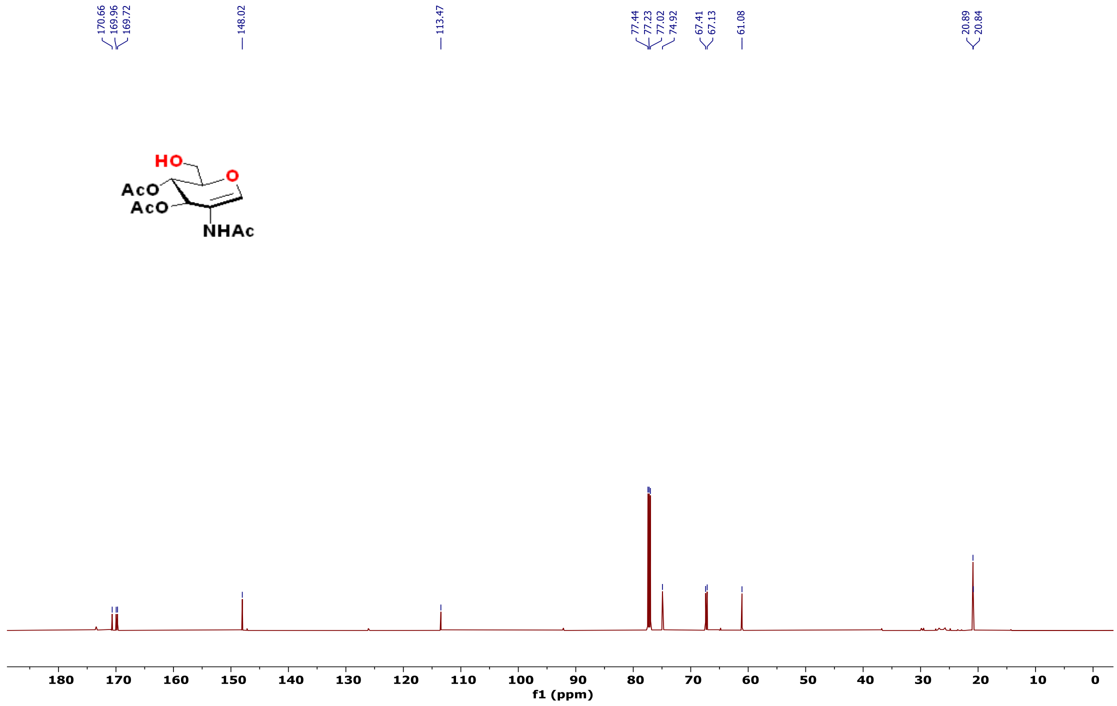

Figure S35. ^{1}H and ^{13}C NMR spectra.

^{1}H NMR (600 MHz, D_2O)

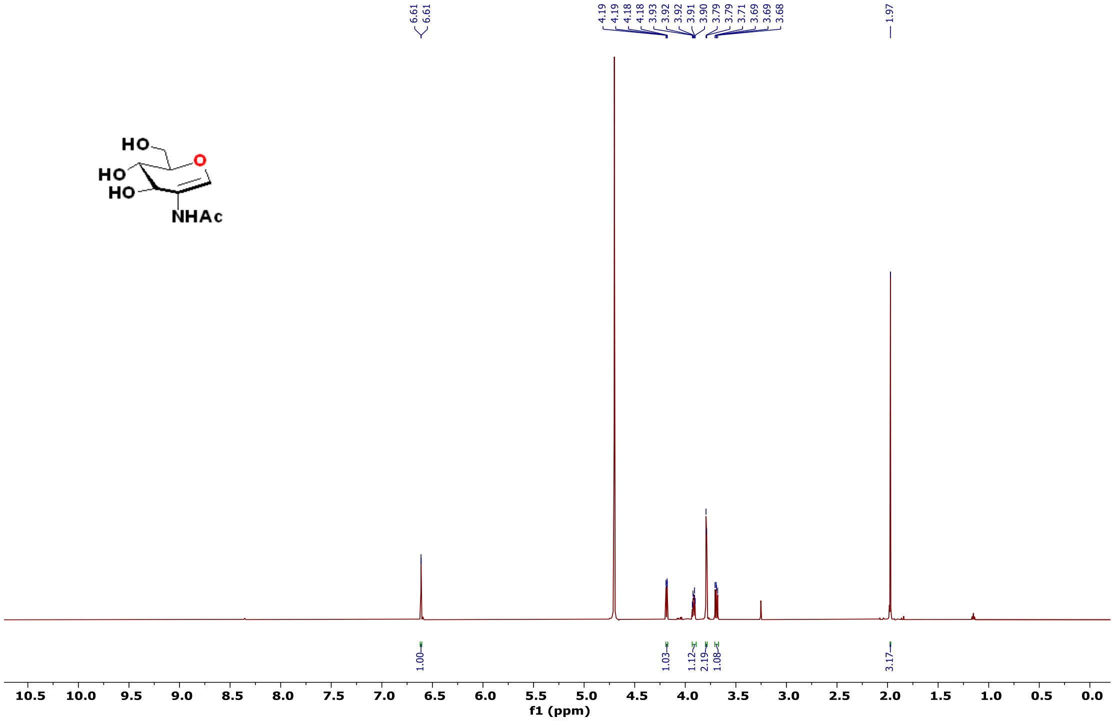

^{13}C NMR (150 MHz, D_2O)

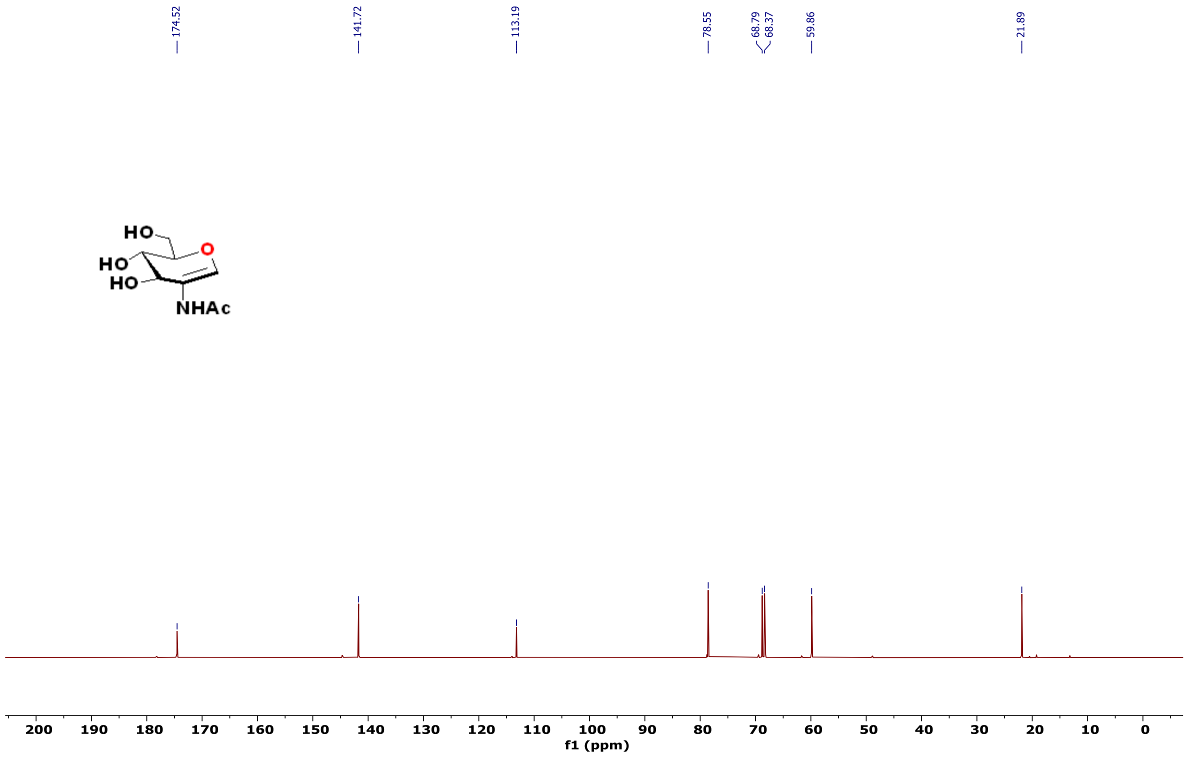

Figure S36. ^{1}H and ^{13}C NMR spectra.

Tables

Table S1. Thermodynamic parameters of KasH for its binding affinity with testing ligands

S.No	KasH	n	K_d (μM)	ΔG (kcal mol^{-1})	ΔH (kcal mol^{-1})	ΔS (cal mol^{-1} K^{-1})
1	CoA	0.6 ± 0.0	96.1	-5.478 ± 1.342	-7.350 ± 1.342	-6.28
2	AcCoA	1.3 ± 0.4	160.7	-5.240 ± 8.196	-14.19 ± 8.196	-30.02
3	Isovaleryl-CoA	1.0 ± 0.7	199.2	-5.048 ± 3.166	-3.116 ± 3.166	6.48
4	Acetoacetyl-CoA	0.5 ± 0.1	98.0	-5.469 ± 2.477	-6.149 ± 2.477	-2.28
5	Propionyl-CoA	0.5 ± 0.0	35.0	-6.081 ± 0.3918	-7.351 ± 0.3918	-4.26
6	KSM	1.0 ± 0.1	56.1	-5.804 ± 0.4705	-2.316 ± 0.4705	11.7

Table S2. Kinetics of KasQ_WT in reaction with UDP-GlcNAc and $MgCl_2$

Enzyme	Substrate	k_{cat} (s^{-1})	K_M(mM)	k_{cat}/K_M (s^{-1} mM^{-1})
1. KasQ_WT	UDP-GlcNAC	1.33 (± 0.24)	40.28 (± 4.0)	0.032

Table S3. Thermodynamic parameters of KasQ mutants in the binding affinity assay

S.No	KasQ Mutants/ NDP-Sugars	n	K_d (μM)	ΔG (kcal mol^{-1})	ΔH (kcal mol^{-1})	ΔS (cal mol^{-1} K^{-1})
1	Q95A/UDP-GlcNAc	1.6 ± 0.1	45.2	-5.939 ± 0.1764	-0.7212 ± 0.1764	17.5
2	Q95A/UDP-Glc	1.0 ± 1.4	362. 3	-4.697± 7.991	-4.321 ± 7.991	1.26
3	Q95A/UDP-Gal	NBD	NBD	NBD	NBD	NBD
4	Q95A/UDP-GlcA	1.7 ± 0.0	44.4	-5.922 ± 0.0847	-0.6748 ± 0.0847	17.6
5	Q95E/UDP-GlcNAc	0.7 ± 0.0	14.2	-6.607 ± 0.0679	-0.6141 ± 0.0679	20.1
6	Q95E/UDP-Glc	0.7 ± 0.5	138.3	-5.266 ± 2.137	-2.225 ± 2.137	10.2
7	Q95E/UDP-Gal	1.0 ± 0.4	109.4	-5.403 ± 1.310	-1.795 ± 1.310	12.1
8	Q95E/UDP-GlcA	2.1 ± 0.1	11.8	-7.15 ± 0.0195	-0.4413 ± 0.0195	22.5
9	E308A/UDP-GlcNAc	NBD	NBD	NBD	NBD	NBD
10	E308A/UDP-Glc	NBD	NBD	NBD	NBD	NBD
11	E308A/UDP-Gal	1.0 ± 0.0	111.1	-5.395 ± 1.031	-6.573 ± 1.031	-3.95
12	E308A/UDP-GlcA	1.1 ± 0.7	106.1	-5.421 ± 3.692	-3.194 ± 3.692	7.47
13	E308Q/UDP-GlcNAc	NBD	NBD	NBD	NBD	NBD
14	E308Q/UDP-Glc	NBD	NBD	NBD	NBD	NBD
15	E308Q/UDP-Gal	1.0 ± 0.2	128.5	-5.321 ± 3.037	-9.465 ± 3.037	-13.9

NBD* No Binding Detected
